# TBHP-mediated oxidative synthesis of substituted pyrimido[4,5-*d*]pyrimidines from *N*-uracil amidines and methylarenes under metal free conditions†

**DOI:** 10.1039/c9ra06625j

**Published:** 2019-09-20

**Authors:** Pradip Debnath

**Affiliations:** Department of Chemistry, Maharaja Bir Bikram College Agartala Tripura-799004 India pradipchem78@gmail.com pdn0099@yahoo.co.in +3812516728 +3812526607

## Abstract

An efficient and operationally simple protocol has been demonstrated for the synthesis of 1,3,5,7-tetrasubstituted pyrimido[4,5-*d*]pyrimidines *via* TBHP-mediated direct oxidative coupling of *N*-uracil amidines and methylarenes under metal-free conditions. Due to the inherent stability of methylarenes compared to aldehydes, the presented synthetic protocol is adaptable to a broad substrate scope, is operationally simple, has no need for stringent protection in the whole preparation process, and has the potential to prepare valuable products that are currently inaccessible or challenging to prepare using conventional methods. It is a significantly important complement to the conventional synthetic methods. The reaction possesses an efficient tandem oxidation–imination–cyclization process.

## Abstract 2

An efficient and operationally simple protocol has been described for the synthesis of 1,3,5,7-tetrasubstituted pyrimido[4,5-*d*]pyrimidines *via* TBHP-mediated direct oxidative coupling of *N*-uracil amidines and methylarenes under metal-free conditions.

## Graphical abstract

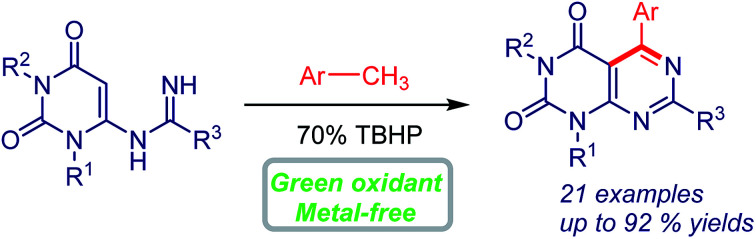

## Introduction

**Scheme 1:**
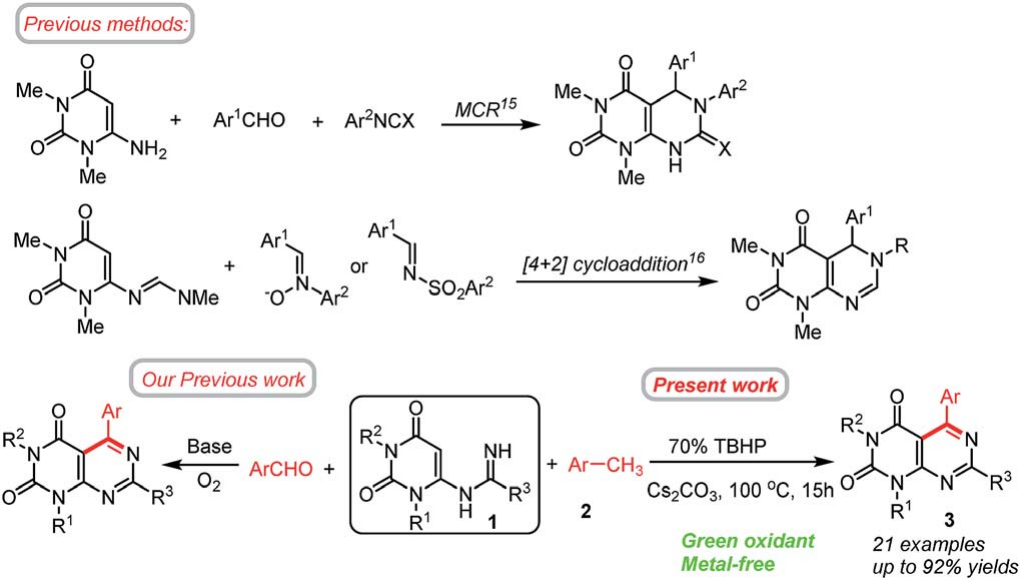
Selected methods for the synthesis of pyrimido[4,5-*d*]pyrimidines from 6-substituted uracil.

Nitrogen containing heterocycles play leading roles in the field of biological and medicinal chemistry.^1^ These molecules have also been extensively used in the fields of agrochemicals, pesticides and pharmaceuticals, and in industry.^2^ Over the past few decades, great efforts have been made to develop novel and efficient methods for the construction of nitrogen containing heterocycles.^3^ Pyrimidines are one of the most important nitrogen heterocycles, exhibiting remarkable pharmaceutical activities.^4^ Among them, pyrimido[4,5-*d*]pyrimidines are very important scaffolds found in many natural products and synthetic drugs or drug candidates exhibiting a wide range of biological activities.^5^ These compounds have attracted considerable attention in medicinal chemistry due to their significant and diverse biological activities, including anticancer,^6^ antiviral,^7^ antifungal,^8*a*^ antioxidant,^8*b*^ antitumor,^9^ and hepatoprotective.^10^ Some of their derivatives are also useful as bronchodilators,^6^ vasodilators,^11^ antiallergic,^12^ antihypertensive^13^ agents. Dipyridamole, a pyrimidopyrimidine based heterocycles, is a medicine that inhibits the phosphodiesterase enzyme, lowers pulmonary hypertension,^13*b*^ and is also used in electro-cardiogram and echocardiography. Recently, pyrimido[4,5-*d*]pyrimidine analogues of folic acid have also been screened for antitumor activity.^14^ Despite these enormous applications, the approaches for the preparation of this type of compounds still remain scarce.^15,16^ According to the literature survey, the previously reported methods for the preparation of pyrimido[4,5-*d*]pyrimidines are highlighted in Scheme 1. The most frequent routes to pyrimido[4,5-*d*]pyrimidines typically involve three-component reactions involving 6-aminouracils^15^ or by the cycloaddition reactions^16^ between 6-methylideneaminouracils and electron-deficient substrates. The latter method is restricted to electron deficient substrates and does not offer unrestricted scope for the synthesis of products. Other promising methods for the synthesis of pyrimido[4,5-*d*]pyrimidines involve multistep syntheses starting from 1,3-disubstituted cyanouracils,^17^ polymer bounded 2-(alkylsulfanyl)-4-aminopyrimidine-5-carbonitrile *etc.* A new approach to the synthesis of pyrimido[4,5-*d*]pyrimidines reported by Wamhoff *et al.*^18^ is the aza-Wittig type reaction of iminophosphoranes of 5-aminouracils leading to functionalised pyrimido[4,5-*d*]pyrimidines. Wang and co-workers reported a protocol for the synthesis of tetrasubstituted pyrimido[4,5-*d*]-pyrimidine derivatives by sequential reactions of 6-aminouracil with hydrazine involving 6 steps.^19^ Tummaluru and co-workers also developed a one-pot protocol for the synthesis of 5,6-dihydropyrimido[4,5-*d*]pyrimidines involving 6-amino-*N*,*N*-dimethyluracil, phenylisocyanate, and aromatic aldehydes in water solvent.^20^ In another approach, Ghorbani-Vaghei and Sarmast employed a magnetic nanoparticle Fe_3_O_4_@SiO_2_ as catalyst in water solvent for the synthesis of substituted pyrimido[4,5-*d*]pyrimidines.^21^ Nevertheless, these methods are associated with few shortcomings such as – harsh reaction conditions (high temperature, microwave),^16*a*,*b*^ use of toxic solvents, use of metal catalysts, or use of complex synthetic pathways,^19^ and less-environmentally benign, or not readily available substrates.^22*a*,23^ So, there is a necessity to develop more effective and sustainable chemical procedures for the synthesis of pyrimido[4,5-*d*]pyrimidines. More viable and efficient approaches are being tried to alleviate the shortcomings. Recently, Deb and co-workers have developed a new strategy for the synthesis of 5,6-dihydropyrimido[4,5-*d*]pyrimidines based on a cascade 3-component reaction involving 6-aminouracil, aldehydes and tetrahydroisoquinolines under solvent free reaction conditions.^24^ Very recently, we have shown that pyrimido[4,5-*d*]pyrimidines could be achieved by the direct annulation of *N*-uracil amidines with benzaldehydes under transition metal free conditions.^25^ Although, this method is highly efficient but the use of aldehydes as the coupling partners could frequently meet some problems such as – (i) the active aldehyde groups may suffer from an oxidation reaction leading to the formation of undesirable by-products, and hence necessitating the requirement for inert conditions;^26^ (ii) aldehydes could undergo a decarbonylation reaction under harsh reaction conditions resulting in lower yields of the products;^27^ (iii) cost of some aldehydes are high or not readily available. On the basis of these facts, the search for readily available, in expensive and stable alternatives of aldehydes would provide a new avenue for the synthesis of pyrimido[4,5-*d*]pyrimidines and is of high importance.

In recent years, the use of abundant and sustainable methylarenes for carbon–carbon (C–C) and carbon–nitrogen (C–N) bond formations have received considerable attentions.^28,29^ Methylarenes are cheap, stable, less toxic, commercially available, and easy to handle, thus making it advantageous to be used as ideal starting materials. By exploring suitable oxidant systems, the oxidation of methylarenes leading to *in situ* formation of aldehydes is considered to be the key step for the formation of desired products.^30,31^ Based on these elegant contributions, we believed that methylarenes could be applied as latent aldehydes^32^ for the synthesis of aryl substituted pyrimido[4,5-*d*]pyrimidines. Compared to aldehydes, methylarenes have a variety of advantages such as cost-effectiveness, thermodynamic stability, abundance and sustainability. On the other hand, burgeoning metal-free organo-catalyst C–H functionalization strategies have attracted much attention in recent years in many oxidation processes with TBHP as an oxidant, which have outstanding performances and advantages such as inexpensive, safe, environmentally benign, mild conditions, and ease of purification.^32,33^

Herein, we wish to develop a new method for the synthesis of N1, N3, C5 and C7 tetrasubstituted pyrimido[4,5-*d*]pyrimidines directly from *N*-uracil amidine and methylarenes under meta-free conditions. S_N_AE of the C6 halogen of 6-chlorouracil by an amidine leads to an *N*-uracil amidine (1),^34^ which can be converted to the corresponding pyrimido[4,5-*d*]pyrimidines (3) by the oxidative coupling with methylarenes (2) using TBHP as a green oxidant (Scheme 1). The reaction possesses an efficient oxidation–imination–cyclization tandem process. The requisite amidine substrates can be easily prepared using a Pinner approach,^35^ starting from the corresponding nitriles and ammonia, and allowing installation of R at C7 position. To the best of our knowledge, such a synthetic protocol has not been reported.

## Results and discussion

At the outset, we began our study by investigating the TBHP mediated oxidative coupling of *N*-uracil amidine 1a and toluene 2a under basic medium. We are pleased to obtain the desired product of pyrimido[4,5-*d*]pyrimidine 3a in 52% isolated yield when the reaction was performed using K_2_CO_3_ as base in the presence of 70% TBHP (3.0 equiv.) in toluene (2a) at 100 °C (Table 1, entry 1). For optimizing the reaction conditions, the effect of oxidants was investigated, and we found that oxidant plays an important role in this oxidation process. 70% TBHP was found to be superior to TBHP and PhI(OAc)_2_. Other oxidant such as H_2_O_2_, O_2_ were proved completely ineffective (Table 1, entries 4 and 5). Various bases such as K_3_PO_4_, Cs_2_CO_3,_ KOAc, Et_3_N, and DBU were also screened (Table 1, entries 6–10). Cs_2_CO_3_ was found to be the optimum choice and provided 82% yield of the desired product (Table 1, entry 7). The reaction did not take place in the presence of organic bases such as Et_3_N and DBU (entries 8 and 9). No products were obtained in the absence of base or oxidant (Table 1, entries 11 and 12). When the amount of oxidant or base were decreased, dramatic lowering of yields was observed (Table 1, entries 13 and 14). Next, the effect of temperature was studied, and it was observed that 100 °C is the optimum reaction temperature. Increase in the reaction temperature did not have any noticeable effect on this transformation, however, the yield of 3a decreased considerably when the reaction was performed at 90 °C (entries 15 and 16). Moreover, the yield of 3a dropped to 57% when the reaction was performed with a mixture 0.5 mL of toluene and 0.5 mL chlorobenzene (entry 17). Thus, the optimized reaction conditions are summarized as follows: *N*-uracil benzamidine (1a, 0.5 mmol), toluene (2a, 1 mL), Cs_2_CO_3_ (1.0 mmol, 2.0 equiv.), 70% TBHP (3.0 equiv.) at 100 °C for 15 h under air (Table 1, entry 7).

**Table 1:** Optimization of the reaction conditions for the synthesis of pyrimido[4,5-*d*]pyrimidine (3a)^a^

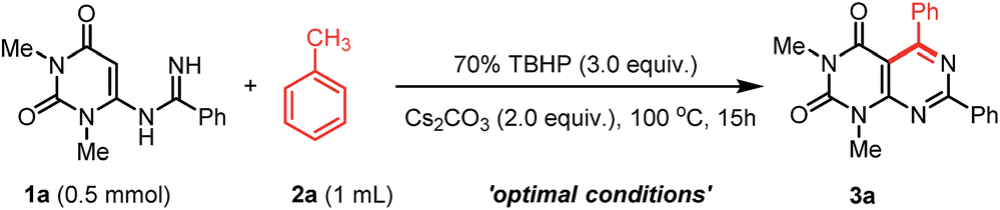			
Entry	Oxidant	Base	Yield^b^ (%)
1	70% TBHP	K_2_CO_3_	52
2	TBHP	K_2_CO_3_	37
3	PhI(OAc)_2_	K_2_CO_3_	21
4	H_2_O_2_	K_2_CO_3_	Trace^c^
5	O_2_	K_2_CO_3_	0
6	70% TBHP	K_3_PO_4_	45
7	70% TBHP	Cs_2_CO_3_	82
8	70% TBHP	Et_3_N	0
9	70% TBHP	DBU	0
10	70% TBHP	KOAc	25
11	70% TBHP	—	0
12	—	Cs_2_CO_3_	0
13^d^	70% TBHP	Cs_2_CO_3_	28
14^e^	70% TBHP	Cs_2_CO_3_	40
15^f^	70% TBHP	Cs_2_CO_3_	79
16^f^	70% TBHP	Cs_2_CO_3_	64
17^g^	70% TBHP	Cs_2_CO_3_	57

a Reaction conditions: all reactions were performed with 1a (0.5 mmol, 1.0 equiv.), oxidant (3.0 equiv.) and base (1.0 mmol, 2.0 equiv.) in 1 mL toluene (2a) at 100 °C, 15 h.

b Isolated yield of 3a.

c 1a was recovered.

d 1.5 equiv. of 70% TBHP was used.

e 1.5 equiv. of Cs_2_CO_3_ was used.

f Reaction was carried out at 110 °C (entry 15) and at 90 °C (entry 16).

g Reaction was performed with a mixture of 0.5 mL toluene and 0.5 mL chlorobenzene (entry 17).

**Scheme 2:**
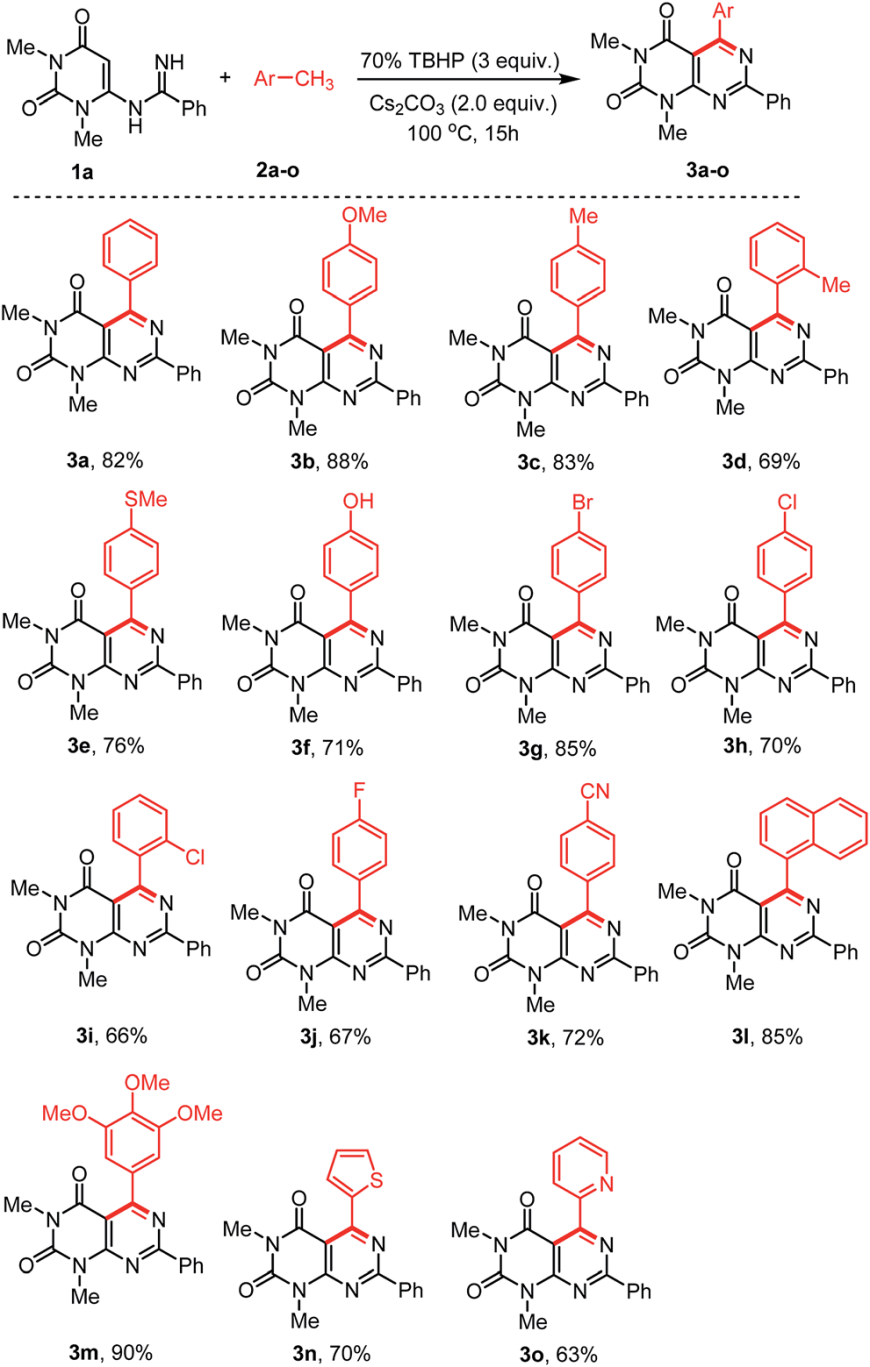
Scope of the arylmethanes (2) in the reaction with *N*-uracil benzamidine (1a).^a,b a^Reaction conditions: substrate 1a (0.5 mmol), 2 (1 mL), 70% TBHP (3.0 equiv.), and Cs_2_CO_3_ (1.0 mmol, 2.0 equiv.), were stirred at 100 °C for 15 h under air. ^b^Yield of the isolated product.

With the optimized conditions in hand, we then tested the generality and limitations of this synthetic protocol. Firstly, the effect of substitution on methylarene was investigated using *N*-uracil benzamidine 1a as reaction partner and the results are listed in Scheme 2. As shown in Scheme 2, all the reactions using methylarenes proceeded smoothly and furnished desired products in good to excellent isolated yields. It was observed that methylarenes with electron-donating substituents such as OMe, Me, SMe, OH produced respective pyrimido[4,5-*d*]-pyrimidines 3b–f and 3m in very good yields (69–90%) while methylarenes having strong electron-withdrawing substituents such as F, CN furnished slightly lower yield of the products 3j and 3k. It was also observed that methylarenes having *para*-substituents afforded the products in higher yields than those with *ortho*-substituents. We reasoned that steric hindrance by a group at the *ortho*-position of methylarenes might be playing a major role in lowering the yield (3d, 3i). It is pertinent to mention that when the toluene derivatives were substituted with more than one methyl group, the reaction took place on one methyl group only and the other methyl group remained unreacted (3c,d). This result might be attributed to that, once one of the methyl groups on the aromatic ring was involved in the reaction and its electron-withdrawing property was unfavourable to the subsequent oxidation of other methyl group.^31,32^ α-Methylnaphthalene could also be employed to react with *N*-uracil amidine under the optimal reaction conditions giving 3l in 85% yield. Even oxidation-sensitive functional groups such as a thiomethyl group (3e) was compatible with the reaction conditions. Upon a thorough literature investigation, it was found that the synthesis of heteroaryl substituted pyrimido[4,5-*d*]pyrimidines has not been explored with the conventional methods. Herein, we wish to prepare this type of compounds by employing our synthetic protocol. Representative heterocyclic methylarenes such as 2-methylthiophene and 2-methylpyridine were selected as coupling patterns for the oxidative coupling with *N*-uracil amidine (1a). Delightfully, both the reactions proceeded smoothly producing the corresponding pyrimido[4,5-*d*]pyrimidines 3n and 3o in 70% and 63% yields, respectively.

**Scheme 3:**
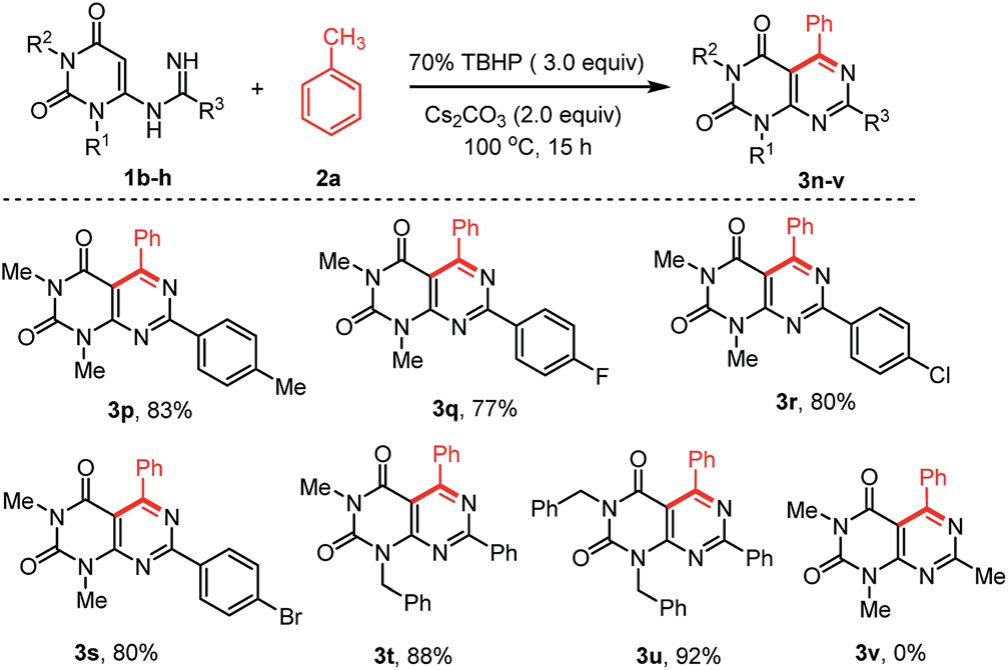
Scope of various *N*-uracil amidine substrates in the reaction with toluene 2a.^a,b a^Reaction conditions: substrate 1 (0.5 mmol), 2a (1 mL), 70% TBHP (3.0 equiv.), and Cs_2_CO_3_ (1.0 mmol, 2.0 equiv.), were stirred at 100 °C for 15 h under air. ^b^Yield of the isolated product.

To extend the scope of the reaction, we also explored the reactions of different *N*-uracil benzimidamides (1b–h) with toluene (1a) under the standard reaction conditions (Scheme 3). The protocol tolerated a variety of substituents on the aryl ring of the *N*-uracil benzimidamide, with both electron-donating and electron-withdrawing groups (3p–u, Scheme 3). Only an exception was observed in case of less stable alkyl amidines such as acetamidine bearing a methyl at the R^3^ position (1h). No 1h was recovered and its decomposition mechanism is not clear. Notably, the *para*-bromo substituted pyrimido[4,5-*d*]pyrimidines 3g and 3s, which can be used for further cross coupling reactions, were obtained in very good yields. As fluorine can have a major influence on the pharmacological properties of a drug,^36^ we were delighted to see that this protocol is also compatible with this halogen (3j and 3q). Finally, it is also possible to work with *N*-protected uracil substrates such as *N*-(1-benzyl-3-methyl-2,6-dioxo-1,2,3,6-tetrahydro-pyrimidin-4-yl)benzimidamide (1f) and *N*-(1,3-dibenzyl-2,6-dioxo-1,2,3,6-tetrahydropyrimidin-4-yl)benz-imidamide (1g) under the optimized reaction conditions. Delightfully, both the reactions proceeded smoothly producing the corresponding pyrimido[4,5-*d*]pyrimidines 3t and 3u in 88% and 92% yields, respectively. Deprotection of these substrates^37^ may allowed post-decoration of the uracil moiety, *via N*-alkylation/arylation or dehydroxychlorination and subsequent consecutive S_N_Ar which is particularly interesting for medicinal chemistry purposes.

**Scheme 4:**
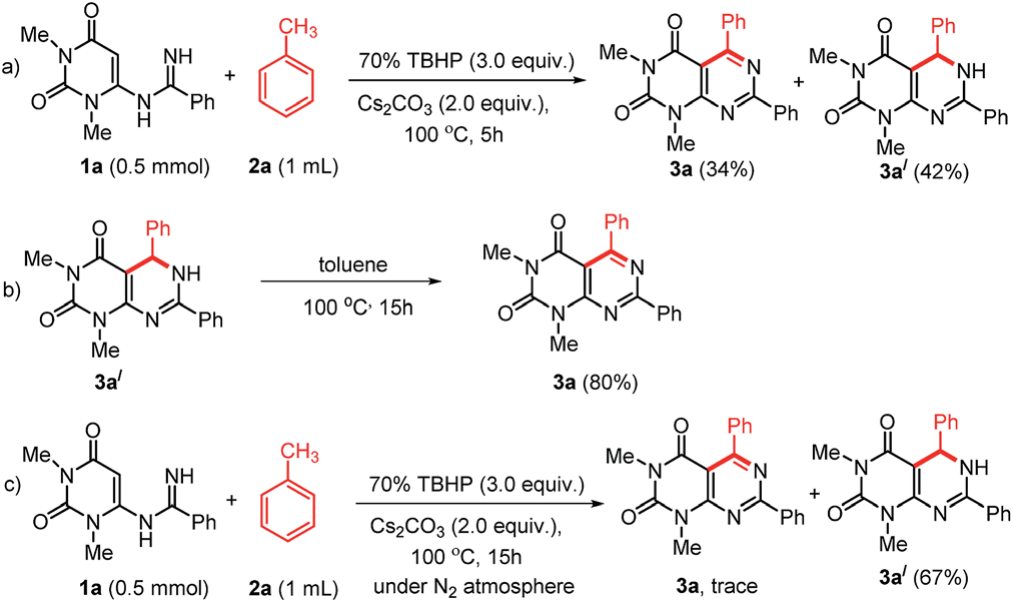
Some control experiments to establish the reaction mechanism.

To study the mechanism of the formation of 3, we performed some control experiments (Scheme 4). We isolated compound 3a′ along with 3a when the reaction was stopped after 5 h (Scheme 4a). When compound 3a′ was heated at 100 °C, it produced 3a in excellent yield (Scheme 4b). Therefore, we believed that the reaction proceeded through the formation of 3a′. As our reactions were performed in open air, we thought aerial oxygen might play some crucial role in the cyclization. In order to confirm the possibility of O_2_ being involved in the oxidation step, a control reaction under N_2_ atmosphere was performed and trace amount of 3a was observed along with 67% of 3a′ (Scheme 4c). These reactions confirm that oxygen is essential for the oxidation of 3a′ into 3a, however, an external supply of oxygen is not required.

**Scheme 5:**
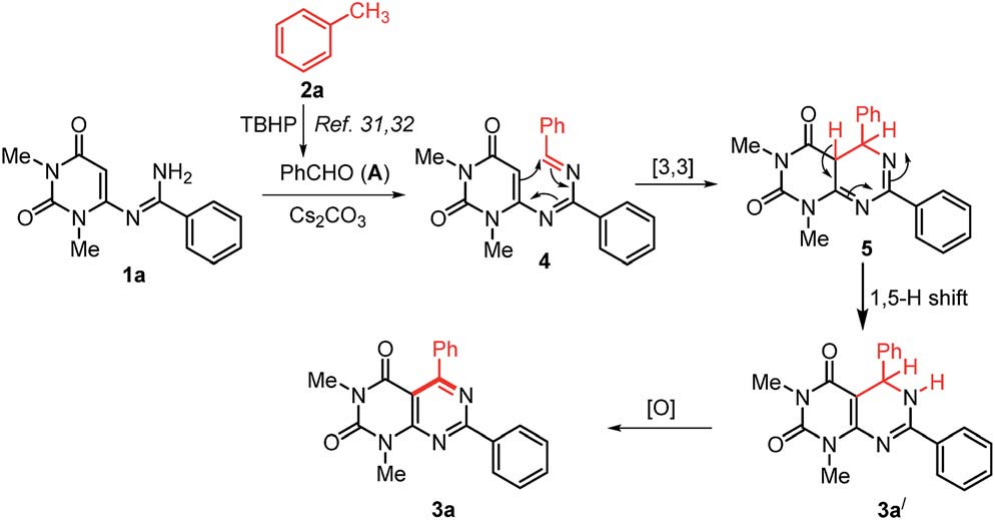
Plausible mechanism for the formation of pyrimido[4,5-*d*]pyrimidines.

On the basis of the previous reports on the progresses about TBHP mediated methylarenes oxidation^30–32^ and based on our control experiments a plausible mechanism for the formation of 3 is proposed in Scheme 5. Initially, the reaction may proceeds with the formation of an aldehyde (A) by TBHP mediated oxidation of toluene.^31,32^ Then, *in situ* generated aldehyde A condensed with *N*-uracil amidine 1a to give azadiene 4.^38^ The intramolecular [4+2] cycloaddition reaction of 4 followed by a [1,5]-hydrogen shift to form the intermediate 5,6-dihydropyrimido[4,5-*d*]pyrimidine 3a′, which on oxidation by aerial oxygen affords the desired product 3a.

## Conclusions

In summary, we have developed an efficient and straightforward method for the synthesis of 1,3,5,7-tetrasubstituted pyrimido[4,5-*d*]pyrimidines from inexpensive and easily available methylarenes and *N*-uracil amidines employing a green oxidant under metal-free conditions. Methylarenes are performed as effective aldehyde precursors in the oxidation–imination–cyclization transformation. Compared to other methods, the present protocol has a number of advantages such as – inherent stability of methylarenes compared to aldehydes, operational simplicity, use of green oxidant, avoidance of metal catalysts, and easy accessibility of the starting materials, making it a highly practical approach to access various pyrimido[4,5-*d*]pyrimidines of biological interest. Hence, it is a significantly superior to the existing methodologies. On the basis of importance of pyrimido[4,5-*d*]pyrimidines in pharmacological science, this protocol has the potential for applications in both drug discovery and chemical development projects.

## Experimental

### General information

All the chemicals and reagents were purchased from commercial suppliers (Sigma-Aldrich, Alfa-Aesar, Spectrochem, TCI Chemicals) and were used without further purification. Silica gel [(60–120, 230–400 mesh), Spectrochem, India] was used for chromatographic separation. Thin-layer chromatography (TLC) was performed on TLC plates purchase from Merck. Solvents used in extraction and purification were distilled prior to use. Melting points were determined by silicon oil bath in open capillaries and are uncorrected. ^1^H (^13^C) NMR spectra were recorded at 400 (100) MHz on a Brucker (Ascend 400 MHz) spectrometer using CDCl_3_ or DMSO-d_6_ solvent with tetramethylsilane as internal standard. Chemical shifts were reported in parts per million (ppm, *δ*) and coupling constants are given in Hertz. Proton coupling patterns are described as singlet (s), doublet (d), triplet (t), multiplet (m). Due to the existence of tautomers, in some cases the amidine NH proton signals in the ^1^H spectrum and amidine carbon in ^13^C spectrum was not detected. Only distinct signals are reported. High resolution mass spectra (HRMS) [Make: Waters; Model: Xevo XS QTof mass spectrometer] were obtained by using positive electrospray ionization (ESI) by Time of Flight (TOF) method.

### General procedure for the TBHP-mediated oxidative synthesis of pyrimido[4,5-*d*]pyrimidines from *N*-uracil amidines and methylarenes

An oven-dried microwave vial (10 mL) equipped with a magnetic stirring bar was charged with *N*-uracil amidine (0.5 mmol, 1.0 equiv.), toluene (1 mL), 70% TBHP (192 mg, 3 equiv.), Cs_2_CO_3_ (325 mg, 1.0 mmol, 2.0 equiv.). The reaction mixture was placed in a pre-heated oil bath at 100 °C and stirred for 15 hours with magnetic stirrer. After completion of 15 h reaction time, the mixture was allowed to reach room temperature and extracted with ethyl acetate (2 × 10 mL). The combined organic layers were washed with water and then brine. The organic layer was dried over anhydrous Na_2_SO_4_, filtered, and concentrated under reduced pressure. The resulting residue was purified by column chromatography on silica gel (60–120 mesh) using hexane–ethyl acetate mixture as eluent to give the title compounds.

### 1,3-Dimethyl-5,7-diphenylpyrimido[4,5-*d*]pyrimidine-2,4(1*H*,3*H*)-dione (3a)^22*a*,25^

White solid; *R*_f_ = 0.4 (SiO_2_, hexane/ethyl acetate = 10 : 90); yield: 82% (141 mg, 0.41 mmol), mp 210–211 °C. ^1^H NMR (400 MHz, CDCl_3_): *δ* = 3.41 (s, 3H), 3.85 (s, 3H), 7.48–7.57 (m, 6H), 7.64 (dd, *J* = 2.0 Hz, 8.4 Hz, 2H), 8.57 (d, *J* = 8.4 Hz, 2H). ^13^C NMR (100 MHz, CDCl_3_): *δ* = 28.6, 29.9, 103.7, 127.7, 128.6, 129.1, 129.3, 130.0, 132.3, 136.1, 138.4, 151.3, 157.9, 159.6, 165.2, 170.4. HRMS (ESI): *m*/*z* calcd for C_20_H_16_N_4_O_2_Na [M^+^ + Na]: 367.1165; found: 367.1158.

### 5-(4-Methoxyphenyl)-1,3-dimethyl-7-phenylpyrimido[4,5-*d*]pyrimidine-2,4(1*H*,3*H*)-dione (3b)^25^

White solid; *R*_f_ = 0.4 (SiO_2_, hexane/ethyl acetate = 10 : 90); yield: 88% (164 mg, 0.44 mmol), mp 235–236 °C. ^1^H NMR (400 MHz, CDCl_3_): *δ* = 3.43 (s, 3H), 3.85 (s, 3H), 3.89 (s, 3H), 7.01 (dd, *J* = 2.0 Hz, 6.8 Hz, 2H), 7.49–7.58 (m, 3H), 7.69–7.72 (m, 2H), 8.58 (dd, *J* = 1.6 Hz, 8.4 Hz, 2H). ^13^C NMR (100 MHz, CDCl_3_): *δ* = 28.7, 29.9, 55.4, 103.2, 113.1, 128.6, 129.2, 130.4, 131.6, 132.2, 136.2, 151.4, 158.1, 159.9, 161.5, 164.9, 169.7. HRMS (ESI): *m*/*z* calcd for C_21_H_18_N_4_O_3_Na [M^+^ + Na]: 397.1271; found: 397.1263.

### 1,3-Dimethyl-5-(4-methylphenyl)-7-phenylpyrimido[4,5-*d*]pyrimidine-2,4(1*H*,3*H*)-dione (3c)^25^

White solid; *R*_f_ = 0.4 (SiO_2_, hexane/ethyl acetate = 5 : 95); yield: 83% (149 mg, 0.415 mmol), mp 226–228 °C. ^1^H NMR (400 MHz, CDCl_3_): *δ* = 2.45 (s, 3H), 3.41 (s, 3H), 3.85 (s, 3H), 7.30 (d, *J* = 8.0 Hz, 2H) 7.48–7.58 (m, 5H), 8.57 (dd, *J* = 1.6 Hz, 8.4 Hz, 2H). ^13^C NMR (100 MHz, CDCl_3_): *δ* = 21.6, 28.6, 29.9, 103.5, 128.5, 128.6, 129.3, 132.2, 135.5, 136.1, 140.3, 151.3, 157.9, 159.7, 165.1, 170.4. HRMS (ESI): *m*/*z* calcd for C_21_H_18_N_4_O_2_Na [M^+^ + Na]: 381.1322; found: 381.1316.

### 1,3-Dimethyl-5-(2-methylphenyl)-7-phenylpyrimido[4,5-*d*]pyrimidine-2,4(1*H*,3*H*)-dione (3d)

White solid; *R*_f_ = 0.45 (SiO_2_, hexane/ethyl acetate = 5 : 95); yield: 69% (123 mg, 0.345 mmol), mp 246 °C. ^1^H NMR (400 MHz, CDCl_3_): *δ* = 2.19 (s, 3H), 3.38 (s, 3H), 3.87 (s, 3H), 7.20 (d, *J* = 7.6 Hz, 1H), 7.32 (t, *J* = 7.2 Hz, 2H), 7.39–7.42 (m, 1H) 7.48–7.58 (m, 3H), 8.56 (t, *J* = 1.6 Hz, 2H). ^13^C NMR (100 MHz, CDCl_3_): *δ* = 19.7, 28.5, 29.8, 104.7, 125.5, 127.4, 128.7, 129.0, 129.4, 130.0, 132.3, 134.8, 136.2, 138.9, 151.4, 157.5, 159.3, 165.8, 171.1. HRMS (ESI): *m*/*z* calcd for C_21_H_19_N_4_O_2_ [M^+^ + H]: 359.1508; found: 359.1492.

### 1,3-Dimethyl-5-(4-thiomethylphenyl)-7-phenylpyrimido[4,5-*d*]pyrimidine-2,4(1*H*,3*H*)-dione (3e)

Off-white solid; *R*_f_ = 0.4 (SiO_2_, hexane/ethyl acetate = 20 : 80); yield: 76% (148 mg, 0.38 mmol), mp 270 °C. ^1^H NMR (400 MHz, CDCl_3_): *δ* = 2.55 (s, 3H), 3.42 (s, 3H), 3.85 (s, 3H), 7.34 (d, *J* = 8.4 Hz, 2H), 7.49–7.56 (m, 3H) 7.63 (d, *J* = 8.4 Hz, 2H), 8.56 (t, *J* = 1.2 Hz, 2H). ^13^C NMR (100 MHz, CDCl_3_): *δ* = 15.2, 28.6, 29.9, 103.4, 124.9, 128.6, 129.3, 130.0, 132.3, 134.5, 136.1, 141.9, 151.3, 158.0, 159.8, 165.1, 169.6. HRMS (ESI): *m*/*z* calcd for C_21_H_18_N_4_O_2_SNa [M^+^ + Na]: 413.1043; found: 413.1036.

### 5-(4-Hydroxyphenyl)-1,3-dimethyl-7-phenylpyrimido[4,5-*d*]pyrimidine-2,4(1*H*,3*H*)-dione (3f)^25^

White solid; *R*_f_ = 0.4 (SiO_2_, hexane/ethyl acetate = 20 : 80); yield: 71% (128 mg, 0.355 mmol), mp 246–248 °C. ^1^H NMR (400 MHz, DMSO): *δ* = 3.22 (s, 3H), 3.66 (s, 3H), 6.84 (d, *J* = 8.8 Hz, 2H), 7.54–7.62 (m, 5H), 8.47 (d, *J* = 6.8 Hz, 2H), 10.0 (br s, 1H, OH). ^13^C NMR (100 MHz, DMSO): *δ* = 28.7, 30.0, 103.8, 114.7, 129.0, 129.2, 129.3, 132.3, 132.6, 136.4, 151.4, 158.3, 160.0, 163.6, 168.8. HRMS (ESI): *m*/*z* calcd for C_20_H_17_N_4_O_3_ [M^+^ + H]: 361.1301; found: 361.1294.

### 5-(4-Bromophenyl)-1,3-dimethyl-7-phenylpyrimido[4,5-*d*]pyrimidine-2,4(1*H*,3*H*)-dione (3g)^25^

White solid; *R*_f_ = 0.45 (SiO_2_, hexane/ethyl acetate = 5 : 95); yield: 85% (179 mg, 0.425 mmol), mp 258 °C. ^1^H NMR (400 MHz, CDCl_3_): *δ* = 3.41 (s, 3H), 3.86 (s, 3H), 7.49–7.59 (m, 6H), 7.63 (d, *J* = 8.4 Hz, 1H), 8.58 (d, *J* = 7.2 Hz, 2H). ^13^C NMR (100 MHz, CDCl_3_): *δ* = 28.6, 30.0, 103.6, 124.7, 128.7, 129.3, 130.9, 131.0, 132.5, 135.9, 137.2, 151.2, 157.9, 159.6, 165.4, 169.3. HRMS (ESI): *m*/*z* calcd for C_20_H_16_BrN_4_O_2_ [M^+^ + H]: 423.0457; found: 423.0477.

### 5-(4-Chlorophenyl)-1,3-dimethyl-7-phenylpyrimido[4,5-*d*]pyrimidine-2,4(1*H*,3*H*)-dione (3h)^25^

White solid; *R*_f_ = 0.4 (SiO_2_, hexane/ethyl acetate = 10 : 90); yield: 70% (132 mg, 0.35 mmol), mp 242 °C. ^1^H NMR (400 MHz, CDCl_3_): *δ* = 3.41 (s, 3H), 3.86 (s, 3H), 7.46–7.61 (m, 7H), 8.55 (t, *J* = 1.6 Hz, 2H). ^13^C NMR (100 MHz, CDCl_3_): *δ* = 28.6, 30.0, 103.6, 128.0, 128.7, 129.3, 130.7, 132.5, 136.0, 136.3, 136.7, 151.2, 157.9, 159.6, 165.4, 169.2. HRMS (ESI): *m*/*z* calcd for C_20_H_15_ClN_4_O_2_Na [M^+^ + Na]: 401.0776; found: 401.0766.

### 1,3-Dimethyl-5-(2-chlorophenyl)-7-phenylpyrimido[4,5-*d*]pyrimidine-2,4(1*H*,3*H*)-dione (3i)

White solid; *R*_f_ = 0.4 (SiO_2_, hexane/ethyl acetate = 10 : 90); yield: 66% (125 mg, 0.33 mmol), mp 258–259 °C. ^1^H NMR (400 MHz, CDCl_3_): *δ* = 3.39 (s, 3H), 3.87 (s, 3H), 7.37–7.58 (m, 7H), 8.56 (t, *J* = 1.2 Hz, 2H). ^13^C NMR (100 MHz, CDCl_3_): *δ* = 28.5, 29.8, 105.0, 126.8, 128.7, 128.9, 129.2, 129.4, 130.2, 132.0, 132.4, 136.1, 138.2, 151.3, 157.3, 159.3, 166.2, 167.9. HRMS (ESI): *m*/*z* calcd for C_20_H_16_ClN_4_O_2_ [M^+^ + H]: 379.0962; found: 379.0971.

### 5-(4-Fluorophenyl)-1,3-dimethyl-7-phenylpyrimido[4,5-*d*]pyrimidine-2,4(1*H*,3*H*)-dione (3j)^25^

White solid; *R*_f_ = 0.45 (SiO_2_, hexane/ethyl acetate = 10 : 90); yield: 67% (121 mg, 0.335 mmol), mp 233 °C. ^1^H NMR (400 MHz, CDCl_3_): *δ* = 3.41 (s, 3H), 3.85 (s, 3H), 7.18 (t, *J* = 8.8 Hz, 2H), 7.50–7.56 (m, 3H), 7.66–7.69 (m, 2H), 8.55 (t, *J* = 1.6 Hz, 2H). ^13^C NMR (100 MHz, CDCl_3_): *δ* = 28.6, 29.5, 103.6, 114.8 (d, *J* = 22.0 Hz), 128.7, 129.3, 131.6 (d, *J* = 8.0 Hz), 132.4, 134.22, 134.25, 136.0, 151.2, 158.0, 159.7, 164.0 (d, *J* = 250 Hz), 169.2. HRMS (ESI): *m*/*z* calcd for C_20_H_16_FN_4_O_2_ [M^+^ + H]: 363.1257; found: 363.1268.

### 1,3-Dimethyl-5-(4-cyanophenyl)-7-phenylpyrimido[4,5-*d*]pyrimidine-2,4(1*H*,3*H*)-dione (3k)

Off-white solid; *R*_f_ = 0.4 (SiO_2_, hexane/ethyl acetate = 10 : 90); yield: 72% (133 mg, 0.36 mmol), mp 252 °C. ^1^H NMR (400 MHz, CDCl_3_): *δ* = 3.41 (s, 3H), 3.87 (s, 3H), 7.52 (t, *J* = 6.8 Hz, 2H), 7.65 (d, *J* = 8.4 Hz, 1H), 7.71 (d, *J* = 8.4 Hz, 2H), 7.79 (d, *J* = 8.0 Hz, 2H), 8.55 (d, *J* = 6.8 Hz, 2H). ^13^C NMR (100 MHz, CDCl_3_): *δ* = 28.6, 30.0, 103.7, 118.7, 127.0, 128.8, 129.4, 129.8, 131.5, 132.3, 132.7, 135.7, 142.9, 151.1, 157.9, 159.5, 165.8. HRMS (ESI): *m*/*z* calcd for C_21_H_15_N_5_O_2_Na [M^+^ + Na]: 392.1118; found: 392.1111.

### 1,3-Dimethyl-5-(naphthalen-1-yl)-7-phenylpyrimido[4,5-*d*]pyrimidine-2,4(1*H*,3*H*)-dione (3l)

Pale yellow solid; *R*_f_ = 0.35 (SiO_2_, hexane/ethyl acetate = 10 : 90); yield: 85% (167 mg, 0.425 mmol), mp 264 °C. ^1^H NMR (400 MHz, CDCl_3_): *δ* = 3.29 (s, 3H), 3.91 (s, 3H), 7.36–7.40 (m, 2H) 7.44–7.62 (m, 7H), 7.94 (d, *J* = 8.4 Hz, 1H), 8.0 (d, *J* = 8.4 Hz, 1H), 8.56 (t, *J* = 1.2 Hz, 1H). ^13^C NMR (100 MHz, CDCl_3_): *δ* = 28.5, 29.8, 105.5, 124.6, 125.1, 125.4, 126.0, 126.5, 128.7, 129.1, 129.3, 129.5, 130.8, 132.4, 133.3, 136.1, 136.8, 151.3, 157.6, 159.0, 165.9, 170.2. HRMS (ESI): *m*/*z* calcd for C_24_H_18_N_4_O_2_Na [M^+^ + Na]: 417.1322; found: 417.1315.

### 1,3-Dimethyl-7-phenyl-5-(3,4,5-trimethoxyphenyl)pyrimido[4,5-*d*]pyrimidine-2,4(1*H*,3*H*)-dione (3m)

White solid; *R*_f_ = 0.35 (SiO_2_, hexane/ethyl acetate = 20 : 80); yield: 90% (195 mg, 0.45 mmol), mp 208–209 °C. ^1^H NMR (400 MHz, CDCl_3_): *δ* = 3.45 (s, 3H), 3.89 (s, 3H), 3.93 (s, 6H), 3.97 (s, 3H), 6.92 (s, 2H), 7.52–7.61 (m, 3H), 8.60 (t, *J* = 1.6 Hz, 2H). ^13^C NMR (100 MHz, CDCl_3_): *δ* = 28.7, 30.0, 56.3, 61.0, 103.6, 106.9, 128.7, 129.3, 132.4, 133.6, 136.0, 139.7, 151.3, 152.6, 158.0, 159.5, 165.1, 170.0. HRMS (ESI): *m*/*z* calcd for C_23_H_23_N_4_O_5_ [M^+^ + H]: 435.1663; found: 435.1658.

### 1,3-Dimethyl-7-phenyl-5-(thiophen-2-yl)pyrimido[4,5-*d*]pyrimidine-2,4(1*H*,3*H*)-dione (3n)

Pale yellow solid; *R*_f_ = 0.4 (SiO_2_, hexane/ethyl acetate = 10 : 90); yield: 70% (122 mg, 0.35 mmol), mp 260–261 °C. ^1^H NMR (400 MHz, CDCl_3_): *δ* = 3.48 (s, 3H), 3.79 (s, 3H), 7.21 (t, *J* = 4.0 Hz, 1H), 7.53–7.58 (m, 3H), 7.66 (d, *J* = 4.4 Hz, 1H), 8.54 (d, *J* = 6.8 Hz, 3H). ^13^C NMR (100 MHz, CDCl_3_): *δ* = 28.8, 30.2, 101.8, 128.2, 128.6, 129.0, 132.3, 132.9, 135.0, 135.9, 141.8, 151.0, 158.4, 160.0, 161.3, 164.3. HRMS (ESI): *m*/*z* calcd for C_18_H_14_N_4_O_2_SNa [M^+^ + Na]: 373.0730; found: 373.0723.

### 1,3-Dimethyl-7-phenyl-5-(pyridin-2-yl)pyrimido[4,5-*d*]pyrimidine-2,4(1*H*,3*H*)-dione (3o)

Pale yellow solid; *R*_f_ = 0.45 (SiO_2_, hexane/ethyl acetate = 50 : 50); yield: 63% (109 mg, 0.315 mmol), mp 254 °C. ^1^H NMR (400 MHz, CDCl_3_): *δ* = 3.38 (s, 3H), 3.86 (s, 3H), 7.48–7.56 (m, 4H), 7.63 (d, *J* = 8.0 Hz, 1H), 7.89 (d, *J* = 7.6 Hz, 1H), 8.56 (d, *J* = 7.2 Hz, 2H), 8.73 (d, *J* = 4.4 Hz, 1H). ^13^C NMR (100 MHz, CDCl_3_): *δ* = 28.5, 29.8, 104.3, 122.8, 124.0, 128.7, 129.4, 132.4, 136.0, 136.5, 149.0, 151.3, 156.7, 157.6, 159.3, 165.9, 168.5. HRMS (ESI): *m*/*z* calcd for C_19_H_16_N_5_O_2_ [M^+^ + H]: 346.1298; found: 346.1293.

### 1,3-Dimethyl-5-phenyl-7-(*p*-tolyl)pyrimido[4,5-*d*]pyrimidine-2,4(1*H*,3*H*)-dione (3p)^25^

White solid; *R*_f_ = 0.4 (SiO_2_, hexane/ethyl acetate = 10 : 90); yield: 83% (148 mg, 0.415 mmol), mp 247 °C. ^1^H NMR (400 MHz, CDCl_3_): *δ* = 2.44 (s, 3H), 3.40 (s, 3H), 3.84 (s, 3H), 7.30 (d, *J* = 8.4 Hz, 2H), 7.47–7.53 (m, 3H), 7.64 (dd, *J* = 2.0 Hz, 8.0 Hz, 2H), 8.46 (t, *J* = 8.0 Hz, 2H). ^13^C NMR (100 MHz, CDCl_3_): *δ* = 21.7, 28.6, 29.9, 103.4, 127.7, 128.6, 129.1, 129.3, 129.4, 129.9, 133.5, 138.5, 143.0, 157.8, 159.7, 165.3, 170.3. HRMS (ESI): *m*/*z* calcd for C_21_H_19_N_4_O_2_ [M^+^ + H]: 359.1508; found: 359.1492.

### 7-(4-Fluorophenyl)-1,3-dimethyl-5-phenylpyrimido[4,5-*d*]pyrimidine-2,4(1*H*,3*H*)-dione (3q)^25^

White solid; *R*_f_ = 0.4 (SiO_2_, hexane/ethyl acetate = 10 : 90); yield: 77% (139 mg, 0.385 mmol), mp 243–245 °C. ^1^H NMR (400 MHz, CDCl_3_): *δ* = 3.41 (s, 3H), 3.85 (s, 3H), 7.18 (t, *J* = 8.8 Hz, 2H), 7.48–7.54 (m, 3H), 7.63 (dd, *J* = 1.6 Hz, 8.0 Hz, 2H), 8.60 (dd, *J* = 5.6 Hz, 8.4 Hz, 2H). ^13^C NMR (100 MHz, CDCl_3_): *δ* = 28.6, 29.9, 103.6, 115.7 (d, *J* = 21.0 Hz), 127.7, 129.1, 130.0, 131.7 (d, *J* = 10.0 Hz), 132.4, 138.3, 151.3, 157.9, 159.6, 164.4, 165.6 (d, *J* = 161.0 Hz), 170.5. HRMS (ESI): *m*/*z* calcd for C_20_H_15_FN_4_O_2_Na [M^+^ + Na]: 385.1071; found: 385.1063.

### 7-(4-Chlorophenyl)-1,3-dimethyl-5-phenylpyrimido[4,5-*d*]pyrimidine-2,4(1*H*,3*H*)-dione (3r)^25^

White solid; *R*_f_ = 0.4 (SiO_2_, hexane/ethyl acetate = 10 : 90); yield: 80% (151 mg, 0.40 mmol), mp 259–260 °C. ^1^H NMR (400 MHz, CDCl_3_): *δ* = 3.41 (s, 3H), 3.85 (s, 3H), 7.46–7.54 (m, 5H), 7.62 (t, *J* = 1.6 Hz, 2H), 8.52 (d, *J* = 8.8 Hz, 2H). ^13^C NMR (100 MHz, CDCl_3_): *δ* = 28.6, 29.9, 103.8, 127.8, 128.9, 129.1, 130.1, 130.6, 134.6, 138.3, 138.7, 151.3, 157.9, 159.5, 164.3, 170.5. HRMS (ESI): *m*/*z* calcd for C_20_H_15_ClN_4_O_2_Na [M^+^ + Na]: 401.0776; found: 401.0766.

### 7-(4-Bromophenyl)-1,3-dimethyl-5-phenylpyrimido[4,5-*d*]pyrimidine-2,4(1*H*,3*H*)-dione (3s)^25^

Off-white solid; *R*_f_ = 0.45 (SiO_2_, hexane/ethyl acetate = 10 : 90); yield: 80% (169 mg, 0.40 mmol), mp 255–256 °C. ^1^H NMR (400 MHz, CDCl_3_): *δ* = 3.41 (s, 3H), 3.85 (s, 3H), 7.48–7.54 (m, 3H), 7.61–7.64 (m, 4H), 8.44 (dd, *J* = 1.6 Hz, *J* = 6.8 Hz, 2H). ^13^C NMR (100 MHz, CDCl_3_): *δ* = 28.7, 30.0, 103.9, 127.4, 127.8, 129.1, 130.1, 130.8, 131.9, 135.1, 138.2, 151.3, 157.9, 159.5, 164.4, 170.6. HRMS (ESI): *m*/*z* calcd for C_20_H_16_BrN_4_O_2_ [M^+^ + H]: 423.0451; found: 423.0441.

### 1-Benzyl-3-methyl-5,7-diphenylpyrimido[4,5-*d*]pyrimidine-2,4(1*H*,3*H*)-dione (3t)^25^

White solid; *R*_f_ = 0.4 (SiO_2_, hexane/ethyl acetate = 5 : 95); yield: 88% (185 mg, 0.44 mmol), mp 211–212 °C. ^1^H NMR (400 MHz, CDCl_3_): *δ* = 3.41 (s, 3H), 5.71 (s, 2H), 7.28–7.36 (m, 3H), 7.48–7.57 (m, 6H), 7.60–7.65 (m, 4H), 8.58 (t, *J* = 6.8 Hz, 2H). ^13^C NMR (100 MHz, CDCl_3_): *δ* = 28.7, 45.9, 103.7, 127.7, 128.0, 128.70, 128.74, 128.8, 129.1, 129.4, 130.0, 132.4, 136.2, 136.6, 138.4, 151.2, 157.6, 159.5, 165.3, 170.7. HRMS (ESI): *m*/*z* calcd for C_26_H_20_N_4_O_2_Na [M^+^ + Na]: 443.1478; found: 443.1471.

### 1,3-Dibenzyl-5,7-diphenylpyrimido[4,5-*d*]pyrimidine-2,4(1*H*,3*H*)-dione (3u)^25^

White solid; *R*_f_ = 0.5 (SiO_2_, hexane/ethyl acetate = 5 : 95); yield: 92% (228 mg, 0.46 mmol), mp 215 °C. ^1^H NMR (400 MHz, CDCl_3_): *δ* = 4.70 (s, 2H), 5.21 (s, 2H), 7.26–7.38 (m, 6H), 7.46–7.60 (m, 10H), 7.65 (t, *J* = 1.6 Hz, 2H), 8.56 (t, *J* = 2.0 Hz, 2H). ^13^C NMR (100 MHz, CDCl_3_): *δ* = 45.0, 46.0, 103.9, 127.8, 127.9, 128.5, 128.68, 128.7, 129.1, 129.4, 130.2, 132.3, 136.2, 136.5, 136.6, 138.3, 151.1, 157.7, 159.4, 165.2, 170.7. HRMS (ESI): *m*/*z* calcd for C_32_H_24_N_4_O_2_Na [M^+^ + Na]: 519.1791; found: 519.1782.

### 1,3-Dimethyl-5,7-diphenyl-5,6-dihydropyrimido[4,5-*d*]pyrimidine-2,4(1*H*,3*H*)-dione (3a′)

Yellow solid; *R*_f_ = 0.4 (SiO_2_, hexane/ethyl acetate = 50 : 50); yield: 42% (73 mg, 0.21 mmol), ^1^H NMR (400 MHz, CDCl_3_): *δ* = 3.28 (s, 3H), 3.68 (s, 3H), 5.80 (d, *J* = 2.8 Hz, 1H), 6.81 (br s, 1H, NH), 7.30–7.37 (m, 3H), 7.46–7.53 (m, 4H), 7.61 (t, *J* = 7.6 Hz, 1H), 7.89 (t, *J* = 1.2 Hz, 2H). ^13^C NMR (100 MHz, CDCl_3_): *δ* = 27.8, 29.4, 53.4, 89.8, 126.7, 127.2, 128.5, 128.96, 129.0, 132.6, 133.0, 144.2, 150.6, 152.3, 159.4, 161.4.

## Conflicts of interest

The author declares no conflicts of financial interest.

## Supplementary Material

RA-009-C9RA06625J-s001
