# Supplementary material for: TBHP-mediated oxidative synthesis of substituted pyrimido[4,5-d]pyrimidines from N-uracil amidines and methylarenes under metal free conditions
Source: RSC Adv. 2019 Sep 20;9(51):29831–9. doi: 10.1039/c9ra06625j (PMC9092612; doi:10.1039/c9ra06625j)
Supplement: RA-009-C9RA06625J-s001 [file RA-009-C9RA06625J-s001.pdf]

Electronic Supplementary Material (ESI) for RSC Advances.
This journal is © The Royal Society of Chemistry 2019

Supporting Information

TBHP-Mediated Oxidative Synthesis of Substituted Pyrimido[4,5-*d*]pyrimidines from *N*-Uracil Amidines and Methylarenes Under Metal Free Conditions

Pradip Debnath*[a]
[a]Department of Chemistry, Maharaja Bir Bikram College, Agartala, Tripura-799004, INDIA

Table of Content

1. Instrumentation and chemicals **2**

2. Experimental procedures and characterization **3-7**

3. References **7**

4. NMR-spectra **8-71**

1. Instrumentation and chemicals

*Corresponding author. Tel: 03812526607; Fax: 03812516728; e-mail: pradipchem78@gmail.com; pdn0099@yahoo.co.in

General information

All the chemicals and reagents were purchased from commercial suppliers (Sigma-Aldrich, Alfa-Aesar, Spectrochem, TCI Chemicals) and were used without further purification. Silica gel [(60-120, 230-400 mesh), Spectrochem, India] was used for chromatographic separation. Thin-layer chromatography (TLC) was performed on TLC plates purchase from Merck. Solvents used in extraction and purification were distilled prior to use. Melting points were determined by silicon oil bath in open capillaries and are uncorrected. ^{1}H (^{13}C) NMR spectra were recorded at 400 (100) MHz on a Brucker (Ascend 400 MHz) spectrometer using $CDCl_3$ or DMSO-d_6 solvent with tetramethylsilane as internal standard. Chemical shifts were reported in parts per million (ppm, δ) and coupling constants are given in Hertz. Proton coupling patterns are described as singlet (s), doublet (*d*), triplet (*t*), multiplet (*m*). Due to the existence of tautomers, in some cases the amidine NH proton signals in the ^{1}H spectrum and amidine carbon in ^{13}C spectrum was not detected. Only distinct signals are reported. High resolution mass spectra (HRMS) [Make: Waters; Model: Xevo XS Q-Tof mass spectrometer] were obtained by using positive electrospray ionization (ESI) by Time of Flight (TOF) method.

2. Experimental procedures and characterization:

a) General procedure for the preparation of 1,3-dibenzyl-6-chloropyrimidine-2,4(1*H*,3H)-dione (A) and 1-benzyl-6-chloro-3-methylpyrimidine-2,4(1*H*,3*H*)-dione (B).[1,2]

Chemicals used: (i) 6-chlorouracil (Sigma Aldrich, ≥ 98% pure), (ii) 6-chloro-3-methyluracil (Sigma Aldrich, ≥ 98% pure), (iii) Benzyl bromide (Spectrochem, 97% pure).

An oven-dried 100 mL round-bottomed flask was loaded with 6-chlorouracil (25 mmol, 1 eq.), potassium carbonate (10.37 g, 75 mmol, 3.0 eq.), and DMF (50 mL). Subsequently, benzyl bromide (2.5 eq. for compound **A** and 1.5 eq. for compound **B**) was added by a syringe. The resulting mixture was stirred at 100°C in an oil bath for 4 hours under N_2 atmosphere. The reaction mixture was then filtered through Celite® and rinsed with 30 mL of dichloromethane. The filtrate was concentrated under reduced pressure. The resulting crude product was purified through column chromatography using ethyl acetate/ petroleum ether mixture (4:1) as eluent providing the title compounds.

1,3-Dibenzyl-6-chloropyrimidine-2,4(1*H*,3*H*)-dione (A)[1,2]:

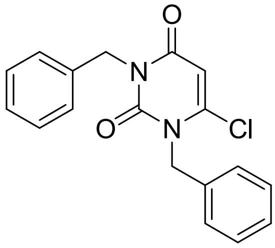

The reaction was carried out with 6-chlorouracil (3.66 g, 25 mmol, 1 eq.), potassium carbonate (10.37 g, 75 mmol, 3.0 eq.) and benzyl bromide (7.4 mL, 62.5 mmol, 2.5 eq.) in DMF (50 mL) solvent during 4 hours of reaction time.
Yield: 84% (6.86 gm, 21.0 mmol), white solid, m.p. 85 ^{0}C. ^{1}H NMR (400 MHz, $CDCl_3$): δ= 5.15 (s, 2H), 5.28 (s, 2H), 5.99 (s, 1H), 7.28-7.38 (m, 8H), 7.49 (d, *J* = 6.4 Hz, 2H). ^{13}C NMR (100 MHz, $CDCl_3$): δ= 45.0, 49.9, 102.6, 127.4, 127.8, 128.2, 128.5, 128.9, 129.0, 135.3, 136.3, 145.9, 151.2, 160.5.

1-Benzyl-6-chloro-3-methylpyrimidine-2,4(1*H*,3*H*)-dione (B):[2]

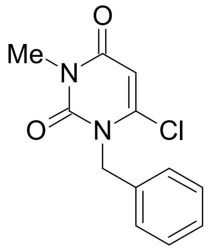

The reaction was carried out with 6-chloro-3-methyluracil (4.014 g, 25 mmol, 1 eq.), potassium carbonate (10.37 g, 75 mmol, 3.0 eq.) and benzyl bromide (4.5 mL, 37.5 mmol, 1.5 eq.) in DMF (50 mL) solvent during 4 hours of reaction time.
Yield: 88% (5.51 g, 22.0 mmol), white solid, m.p. 88 ^{0}C. ^{1}H NMR (400 MHz, $CDCl_3$): δ= 3.38 (s, 3H), 5.30 (s, 2H), 5.98 (s, 1H), 7.32-7.40 (m, 5H). ^{13}C NMR (100 MHz, $CDCl_3$): δ= 28.5, 49.9, 102.3, 127.4, 128.2, 128.9, 135.3, 145.7, 151.3, 160.8.

b) General procedure for the synthesis of *N*-uracil amidine derivatives.[2]

Chemical used: (i) 1,3-Dimethyl-6-chlorouracil (TCI chemicals, 98% purity), (ii) benzamidine hydrochloride (TCI chemicals, 96% purity), (iii) 1,8-diazabicyclo[5.4.0]undec-7-ene (Sigma Aldrich, 98% pure).

An oven-dried 10 mL pressure vial was loaded with 1,3-dimethyl-6-chlorouracil (349 mg, 2 mmol, 1.0 eq.), benzamidine hydrochloride (3.0 mmol, 1.5 equiv.), 1,8-diazabicyclo[5.4.0]undec-7-ene (660 µL, 4.4 mmol, 2.2 equiv.) and anhydrous *tert*-butanol (0.5 mL). The vessel was flushed with N_2 and then sealed with a septum. The resulting mixture was stirred at 80°C under N_2 atmosphere for 24 hours. The reaction mixture was extracted with ethyl acetate (3 x 15 mL), and the combined ethyl acetate was washed with water (20 mL). The organic layer was dried over Na_2SO_4, filtered, concentrated under reduced pressure. The resulting residue was purified by column chromatography using ethyl acetate/petroleum ether mixture as eluent.

c) General procedure for the TBHP-mediated oxidative synthesis of pyrimido[4,5-*d*]pyrimidines from *N*-uracil amidines and methyarenes.

Chemical used: (i) 70% TBHP (Alfa-Aesar), (ii) Methylarenes are purchased from either Sigma-Aldrich, Alfa-Aesar, Spectrochem, TCI Chemicals or Avra Synthesis.

An oven-dried microwave vial (10 mL) equipped with a magnetic stirring bar was charged with *N*-uracil amidine (0.5 mmol, 1.0 equiv.), toluene (1 mL), 70% TBHP (192 mg, 3 equiv.,), Cs_2CO_3 (325 mg, 1.0 mmol, 2.0 equiv). The reaction mixture was placed in a pre-heated oil bath at 100 °C and stirred for 15 hours. After completion of 15 h reaction time, the mixture was allowed to reach room temperature and extracted with ethyl acetate (2 x 10 mL). The combined organic layers were washed with water and then brine. The organic layer was dried over anhydrous Na_2SO_4, filtered, and concentrated under reduced pressure. The resulting residue was purified by column chromatography on silica gel (60-120 mesh) using hexane-ethyl acetate mixture as eluent to give the title compounds.

***N*-(1,3-Dimethyl-2,6-dioxo-1,2,3,6-tetrahydropyrimidin-4-yl)benzimidamide (1a)[1,2]:**
The reaction was carried out with 1,3-dimethyl-6-chloropyrimidine-2,4(1*H*,3*H*)-dione (349 mg, 2.0 mmol, 1.0 eq.) and benzamidine hydrochloride (470 mg, 3.0 mmol, 1.5 eq.) during 24 hours of reaction time. The crude product was purified by column chromatography on silica gel (60-120 mesh) using ethyl acetate-petroleum ether mixture (7:3) as eluent.

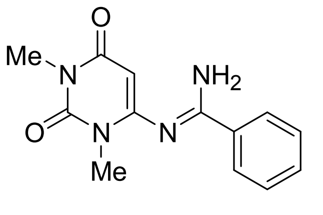

Yield: 70% (361 mg, 1.4 mmol), white solid, m.p. 214-216 ^{0}C. ^{1}H NMR (400 MHz, DMSO): δ= 3.16 (s, 3H), 3.21 (s, 3H), 4.95 (s, 1H), 7.48 (t, *J* = 6.8 Hz, 2H), 7.56 (t, *J* = 7.2 Hz, 1H), 7.90 (t, *J* = 6.8 Hz, 2H). ^{13}C NMR (100 MHz, DMSO): δ= 27.7, 30.2, 87.3, 127.9, 128.8, 131.7, 134.2, 152.8, 158.2, 158.6, 163.0. HRMS (ESI): *m/z* calcd for $C_{13}H_{15}N_4O_2$ [M^++H]: 259.1195; found: 259.1189.

***N*-(1,3-Dimethyl-2,6-dioxo-1,2,3,6-tetrahydropyrimidin-4-yl)-4-methylbenzimidamide (1b)[2]:** The reaction was carried out with 1,3-dimethyl-6-chloropyrimidine-2,4(1*H*,3*H*)-

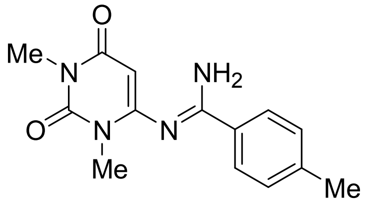

dione (349 mg, 2.0 mmol, 1.0 eq.) and *p*-methylbenzimidamide hydrochloride (512 mg, 3.0 mmol, 1.5 eq.) during 24 hours of reaction time. The crude product was purified by column chromatography on silica gel (60-120 mesh) using ethyl acetate-petroleum ether mixture (7:3) as eluent.

Yield: 60% (326 mg, 1.2 mmol), white solid, m.p: 177-178 ^{0}C. ^{1}H NMR (400 MHz, DMSO): δ= 2.36 (s, 3H), 3.16 (s, 3H), 3.20 (s, 3H), 4.93 (s, 1H), 7.28 (d, *J* = 8.0 Hz, 2H), 7.80 (d, *J* = 8.4 Hz, 2H). ^{13}C NMR (100 MHz, DMSO): δ= 21.4, 27.7, 30.1, 87.2, 127.9, 129.3, 131.3, 141.7, 152.8, 158.2, 158.6, 163.0. HRMS (ESI): *m/z* calcd for $C_{14}H_{17}N_4O_2$ [M^++H]: 273.1352; found: 273.1363.

4-Fluro-*N*-(1,3-dimethyl-2,6-dioxo-1,2,3,6-tetrahydropyrimidin-4-yl)benzimidamide (1c)[2]: The reaction was carried out with 1,3-dimethyl-6-chloropyrimidine-2,4(1*H*,3*H*)-dione (349 mg, 2.0 mmol, 1.0 eq.) and *p*-fluorobenzimidamide hydrochloride (524 mg, 3.0 mmol, 1.5 eq.) during 24 hours of reaction time. The crude product was purified by chromatography on silica gel (60-120 mesh) using ethyl acetate-petroleum ether mixture (7:3) as eluent.

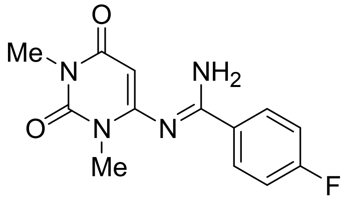

Yield: 67% (370 mg 1.34 mmol), white solid, m.p: 139-141 ^{0}C. ^{1}H NMR (400 MHz, DMSO): δ= 3.17 (s, 3H), 3.20 (s, 3H), 4.98 (s, 1H), 7.33 (t, *J* = 8.8 Hz, 2H), 7.99 (dd, *J* = 5.6 Hz, 7.2Hz, 2H). ^{13}C NMR (100 MHz, DMSO): δ= 27.7, 30.2, 87.4, 115.7 (d, *J* = 21 Hz), 130.5 (d, *J* = 9 Hz), 152.8, 157.1, 158.4, 163.0, 164.4 (d, *J* = 248 Hz). HRMS (ESI): *m/z* calcd for $C_{13}H_{14}FN_4O_2$ [M^++H]: 277.1101; found: 277.1103.

4-Chloro-*N*-(1,3-dimethyl-2,6-dioxo-1,2,3,6-tetrahydropyrimidin-4-yl)benzimidamide (1d)[2]: The reaction was carried out with 1,3-dimethyl-6-chloropyrimidine-2,4(1*H*,3*H*)-dione (349 mg, 2.0 mmol, 1.0 eq.) and *p*-chlorobenzimidamide hydrochloride (573 mg, 3.0 mmol, 1.5 eq.) during 24 hours of reaction time. The crude product was purified by coumn chromatography on silica gel (60-120 mesh) using ethyl acetate-petroleum ether mixture (7:3) as eluent.

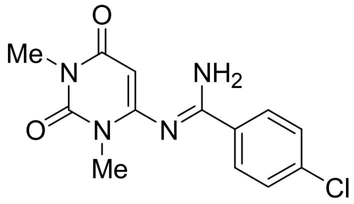

Yield: 68% (397 mg, 1.36 mmol), white solid, m.p: 229-230 ^{0}C. ^{1}H NMR (400 MHz, DMSO): δ= 3.16 (s, 3H), 3.20 (s, 3H), 4.98 (s, 1H), 7.56 (d, *J* = 8.8 Hz, 2H), 7.94 (d, *J* = 8.8 Hz, 2H). ^{13}C NMR (100 MHz, DMSO): δ= 27.7, 30.2, 87.4, 128.9, 129.9, 133.1, 136.5, 152.7, 157.1, 158.3, 163.0. HRMS (ESI): *m/z* calcd for $C_{13}H_{14}ClN_4O_2$ [M^++H]: 293.0805; found: 293.0796.

4-Bromo-*N*-(1,3-dimethyl-2,6-dioxo-1,2,3,6-tetrahydropyrimidin-4-yl)benzimidamide (1e)[2]: The reaction was carried out with 1,3-dimethyl-6-chloropyrimidine-2,4(1*H*,3*H*)-dione (349 mg, 2.0 mmol, 1.0 eq.) and *p*-bromobenzimidamide hydrochloride (706 mg, 3.0 mmol, 1.5 eq.) during 24 hours of reaction time.

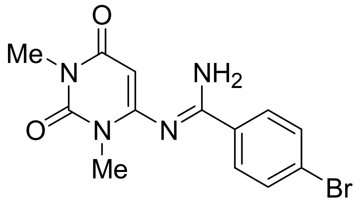

The crude product was purified by column chromatography on silica gel (60-120 mesh) using ethyl acetate-petroleum ether mixture (7:3) as eluent.

Yield: 64% (430 mg, 1.28 mmol), white solid, m.p: 247 ^{0}C. ^{1}H NMR (400 MHz, DMSO): δ= 3.16 (s, 3H), 3.19 (s, 3H), 4.97 (s, 1H), 7.70 (d, J = 8.4 Hz, 2H), 7.85 (d, J = 8.4 Hz, 2H). ^{13}C NMR (100 MHz, DMSO): δ= 27.7, 30.2, 87.4, 125.4, 130.1, 131.8, 133.4, 152.7, 157.3, 158.3, 163.0. HRMS (ESI): *m/z* calcd for $C_{13}H_{14}BrN_4O_2$ [M^++H]: 337.0300; found: 337.0284.

***N*-(3-Benzyl-1-methyl-2,6-dioxo-1,2,3,6-tetrahydropyrimidin-4-yl)benzimidamide (1f):[2]** The reaction was carried out with 1-benzyl-6-chloro-3-methylpyrimidine-2,4(1*H*,3*H*)-dione (501 mg, 2.0 mmol, 1.0 eq.) and benzamidine hydrochloride (470 mg, 3.0 mmol, 1.5 eq.) during 24 hours of reaction time. The crude product was purified by column chromatography on silica gel (60-120 mesh) using ethyl acetate-petroleum ether mixture (1:1) as eluent.

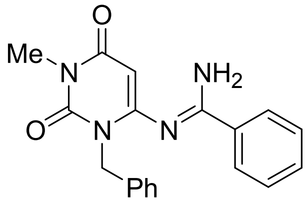

Yield: 55% (367 mg, 1.1 mmol), white solid, m.p. 122 ^{0}C. ^{1}H NMR (400 MHz, $CDCl_3$): δ= 3.35 (s, 3H), 5.21 (s, 2H), 5.27 (s, 1H), 5.64 (s, 2H, NH_2) 7.21-730 (m, 5H), 7.45 (t, J = 7.2 Hz, 2H), 7.55 (t, J = 7.2 Hz, 1H), 7.71 (d, J = 7.6 Hz, 2H). ^{13}C NMR (100 MHz, $CDCl_3$): δ= 27.9, 46.7, 87.7, 127.0, 127.5, 127.9, 128.5, 128.8, 132.0, 133.3, 137.4, 152.7, 157.0, 157.8, 163.8. HRMS (ESI): *m/z* calcd for $C_{19}H_{19}N_4O_2$ [M^++H]: 335.1508; found: 335.1491.

***N*-(1,3-Dibenzyl-2,6-dioxo-1,2,3,6-tetrahydropyrimidin-4-yl)benzimidamide (1g)[1,2]:** The reaction was carried out with 1,3-dibenzyl-6-chloropyrimidine-2,4(1*H*,3*H*)-dione (654 mg, 2.0 mmol, 1.0 eq.) and benzamidine hydrochloride (470 mg, 3.0 mmol, 1.5 eq.) during 24 hours of reaction time. The crude product was purified by column chromatography on silica gel (60-120 mesh) using ethyl acetate-petroleum ether mixture (3:7) as eluent.

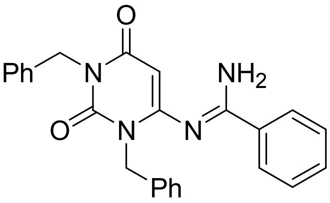

Yield: 53% (435 mg, 1.06 mmol), white solid, m.p. 166-167 ^{0}C. ^{1}H NMR (400 MHz, DMSO): δ= 5.01 (s, 2H), 5.08 (s, 2H), 5.09 (s, 1H), 7.28-7.32 (m, 7H), 7.42-7.47 (m, 3H), 7.50-7.54 (m, 2H), 7.79 (d, J = 7.2 Hz, 2H), 7.88 (d, J = 7.2 Hz, 1H), 8.01 (brs, 1H, NH). ^{13}C NMR (100 MHz, DMSO): δ= 43.8, 46.3, 87.3, 127.4, 127.9, 128.0, 128.7, 128.73, 128.8, 131.7, 131.8, 134.2, 134.7, 138.2, 152.6, 157.9, 158.8, 162.7, 186.4. HRMS (ESI): *m/z* calcd for $C_{25}H_{23}N_4O_2$ [M^++H]: 411.1821; found: 411.1804.

***N*-(1,3-dimethyl-2,6-dioxo-1,2,3,6-tetrahydropyrimidin-4-yl)ethanimidamide (1h):** The reaction was carried out with using 1,3-dimethyl-6-chlorouracil (349 mg, 2.0 mmol, 1.0 eq.) and acetamidine hydrochloride (284 mg, 3.0 mmol, 1.5 eq.) during 24 hours of reaction

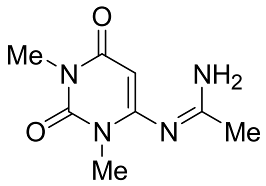

time. The crude product was purified by chromatography on silica gel (60-120 mesh) using 100% ethyl acetate as eluent.

Yield: 40% (157 mg, 0.8 mmol), white solid, m.p. 153-155 ^{0}C. ^{1}H NMR (400 MHz, DMSO): δ= 2.0 (s, 3H), 3.13 (s, 3H), 3.14 (s, 3H), 4.87 (s, 1H), 7.04 (br s, 1H, NH), 7.35 (br s, 1H, NH). ^{13}C NMR (100 MHz, DMSO): δ= 19.8, 27.7, 30.1, 87.1, 152.7, 158.3, 161.3, 163.0. HRMS (ESI): *m/z* calcd for $C_8H_{13}N_4O_2$ [M^{+}+H]: 197.1033; found: 197.1042.

3. References:

1 B. Morel, P. Franck, J. Bidange, S. Sergeyev, D. Smith, J. Moseley and B. U. W. Maes, *ChemSusChem.*, 2017, **10**, 624.

2 P. Debnath, Gouranga Sahu and U. C. De, *ChemistrySelect*, 2019, **4**, 2327.

4. NMR Spectra

1,3-Dibenzyl-6-chloropyrimidine-2,4(1*H*,3*H*)-dione (A):

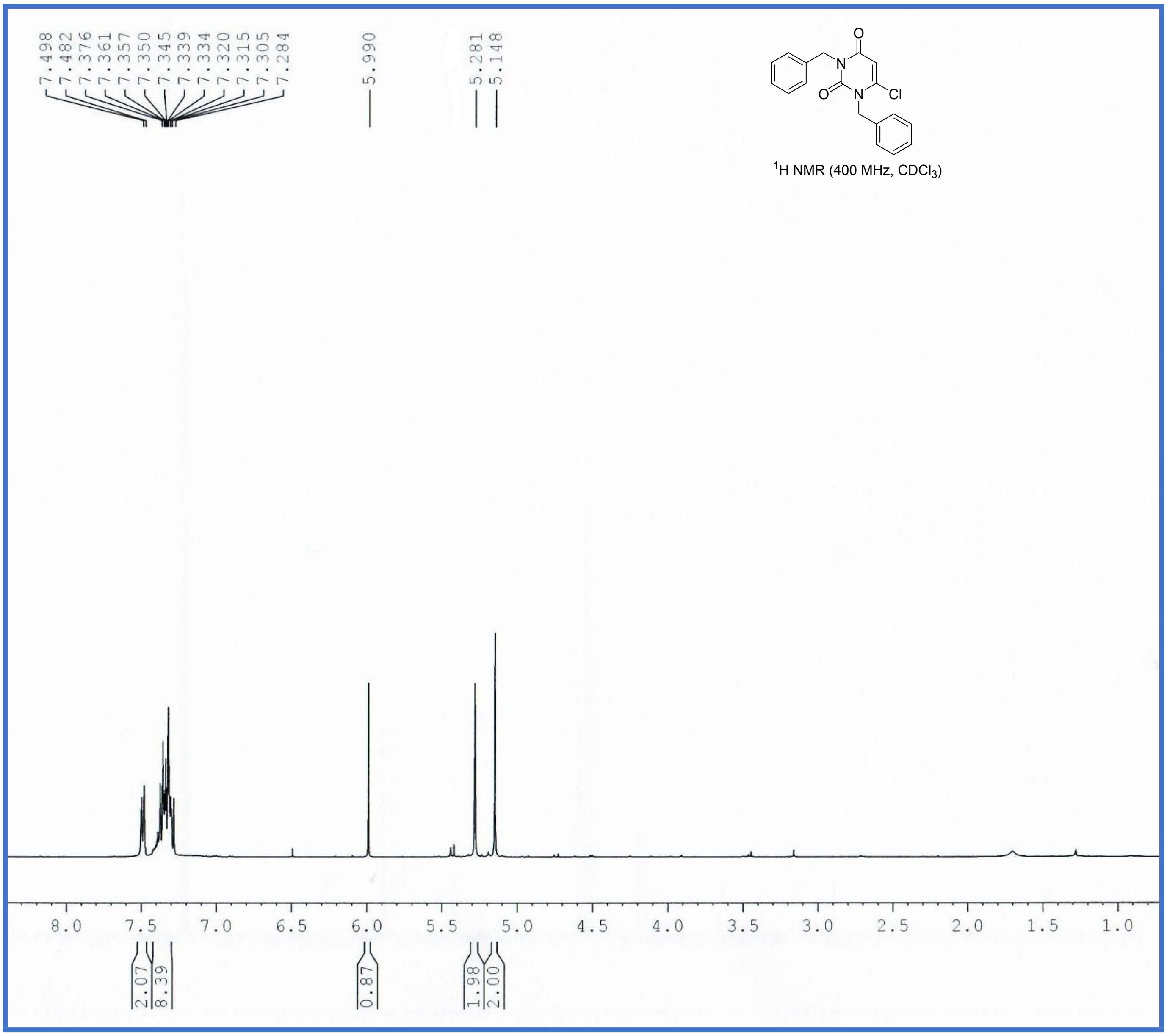
7.498
7.482
7.376
7.361
7.357
7.350
7.345
7.339
7.334
7.320
7.315
7.305
7.284
5.990
5.281
5.148
O
N
O
N
Cl
^{1}H NMR (400 MHz, $CDCl_3$)
8.0
7.5
7.0
6.5
6.0
5.5
5.0
4.5
4.0
3.5
3.0
2.5
2.0
1.5
1.0
2.07
8.39
0.87
1.98
2.00

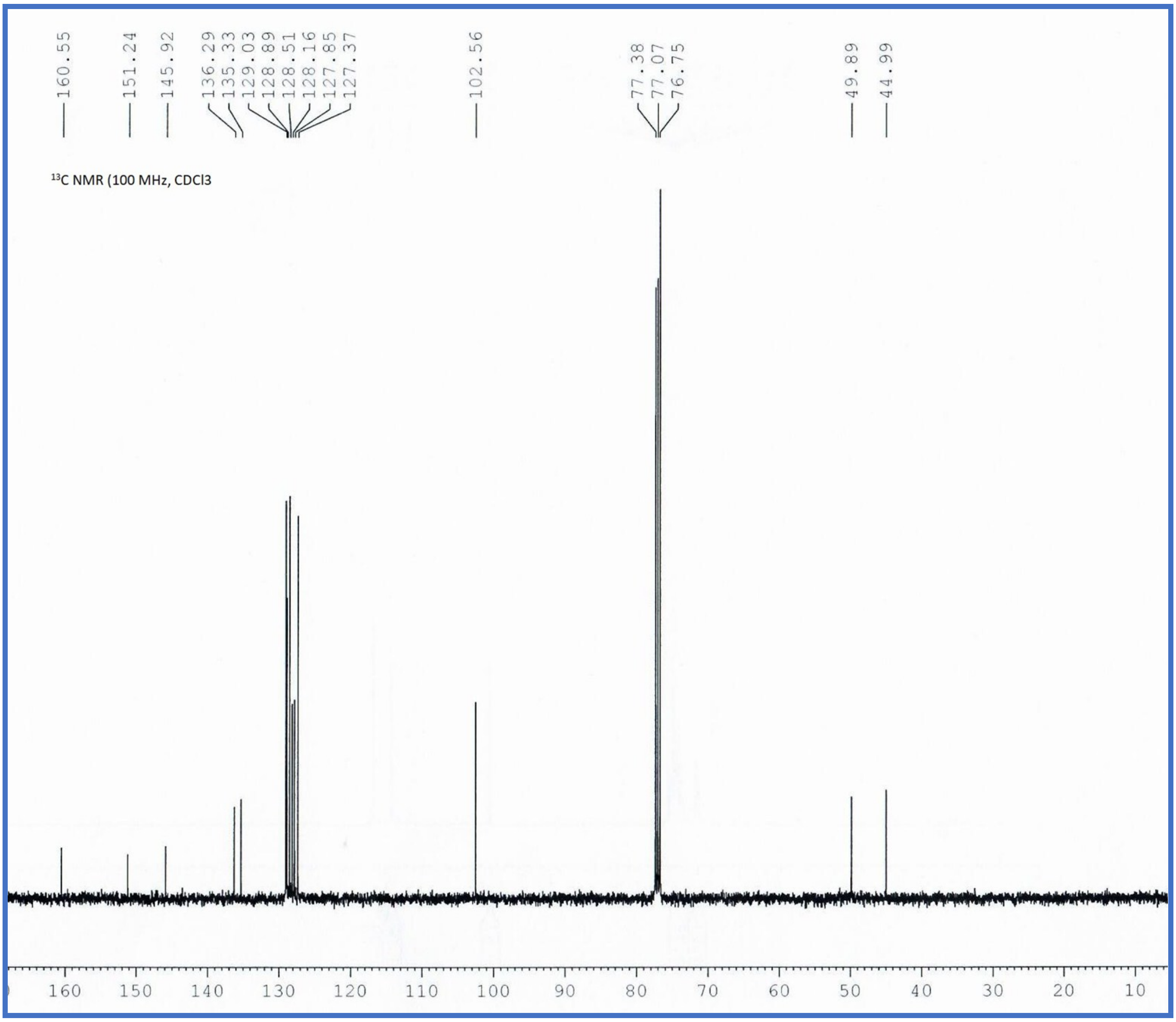
160.55
151.24
145.92
136.29
135.33
129.03
128.89
128.51
128.16
127.85
127.37
102.56
77.38
77.07
76.75
49.89
44.99
13C NMR (100 MHz, CDCl3
160 150 140 130 120 110 100 90 80 70 60 50 40 30 20 10

1-Benzyl-6-chloro-3-methylpyrimidine-2,4(1*H*,3*H*)-dione (B):

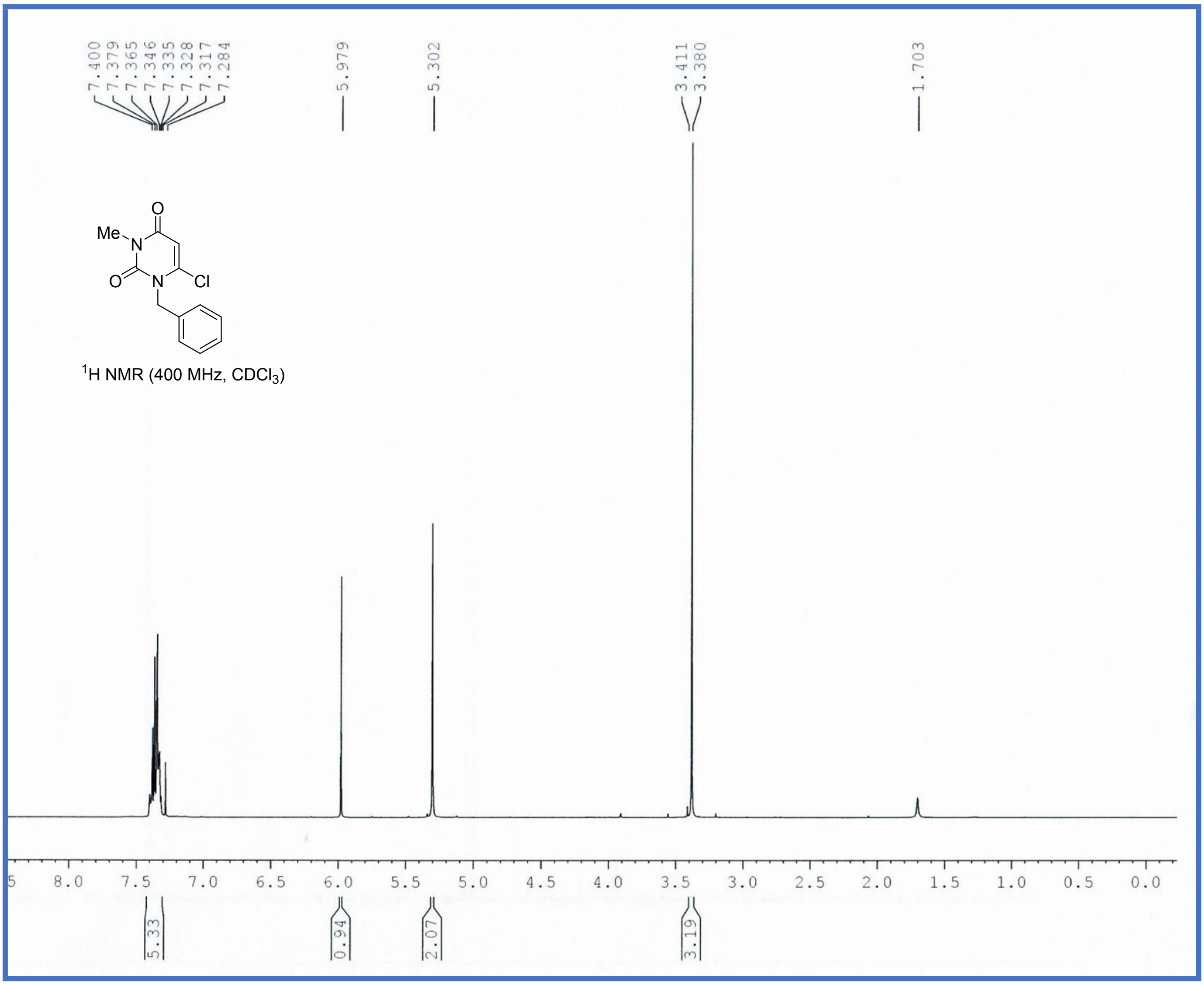

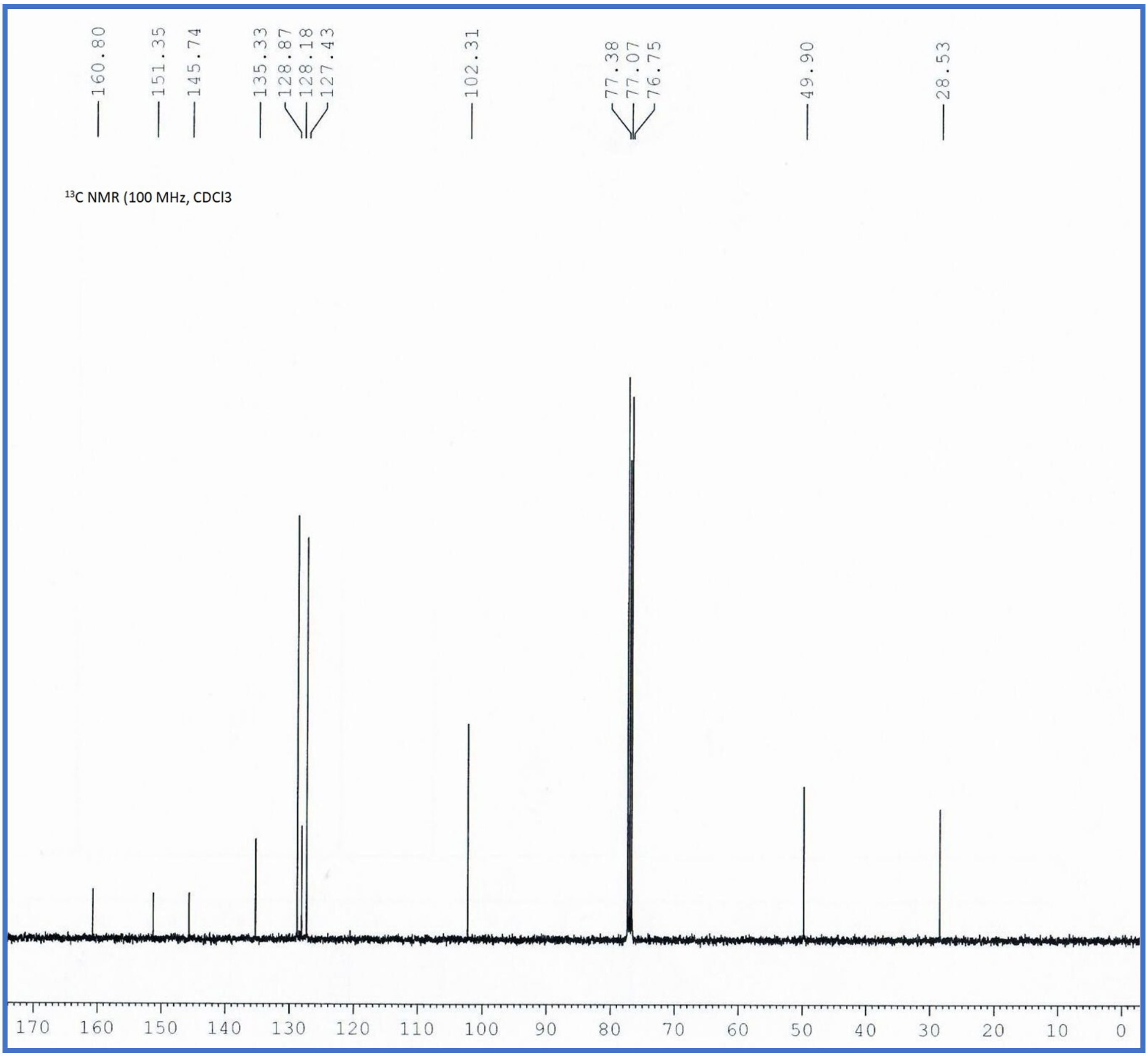
160.80
151.35
145.74
135.33
128.87
128.18
127.43
102.31
77.38
77.07
76.75
49.90
28.53
13C NMR (100 MHz, CDCl3
170 160 150 140 130 120 110 100 90 80 70 60 50 40 30 20 10 0

***N*-(1,3-Dimethyl-2,6-dioxo-1,2,3,6-tetrahydropyrimidin-4-yl)benzimidamide (1a):**

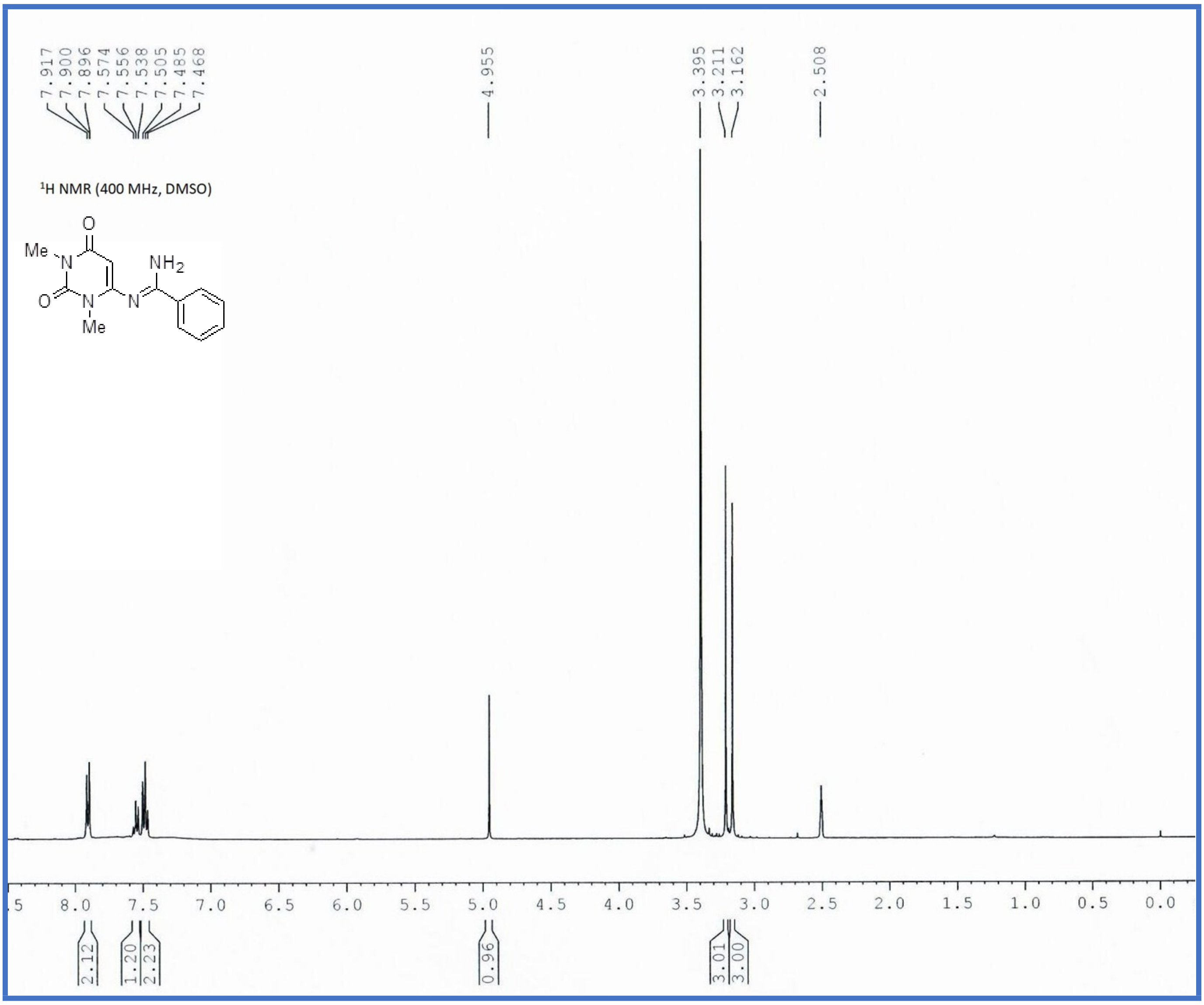

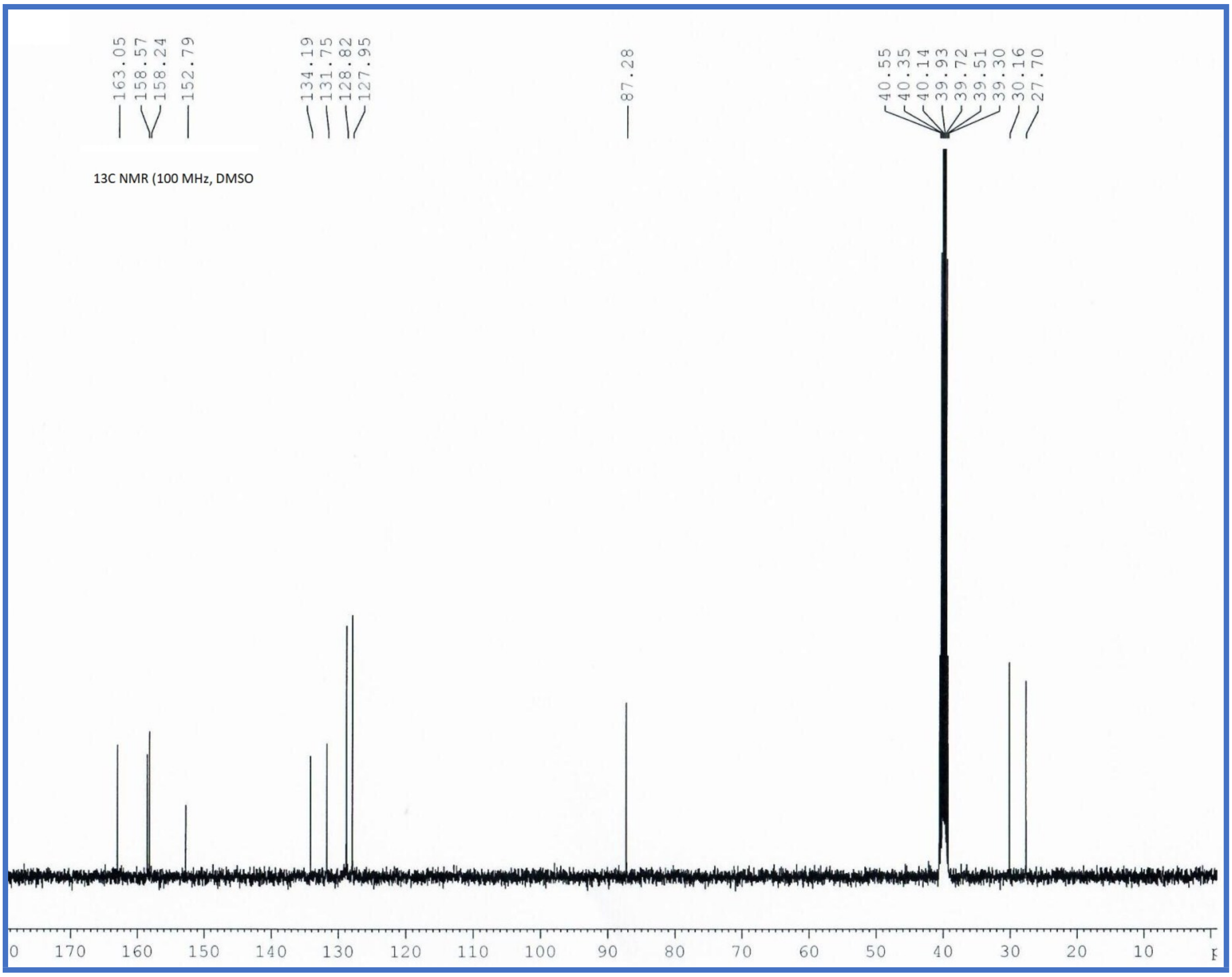
163.05
158.57
158.24
152.79
134.19
131.75
128.82
127.95
87.28
40.55
40.35
40.14
39.93
39.72
39.51
39.30
30.16
27.70
13C NMR (100 MHz, DMSO
170
160
150
140
130
120
110
100
90
80
70
60
50
40
30
20
10

***N*-(1,3-dimethyl-2,6-dioxo-1,2,3,6-tetrahydropyrimidin-4-yl)-4-methylbenzimidamide (1b):**

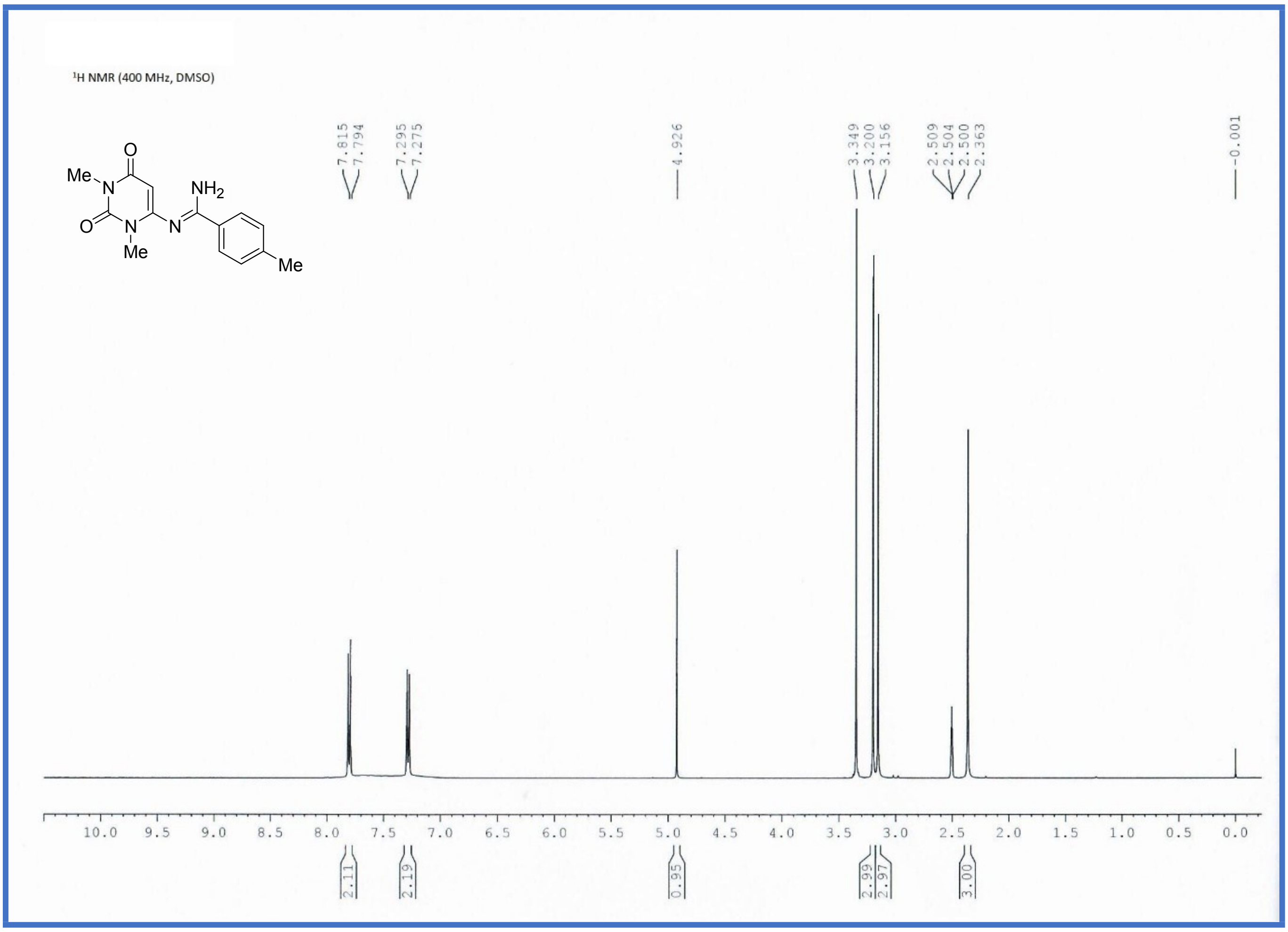

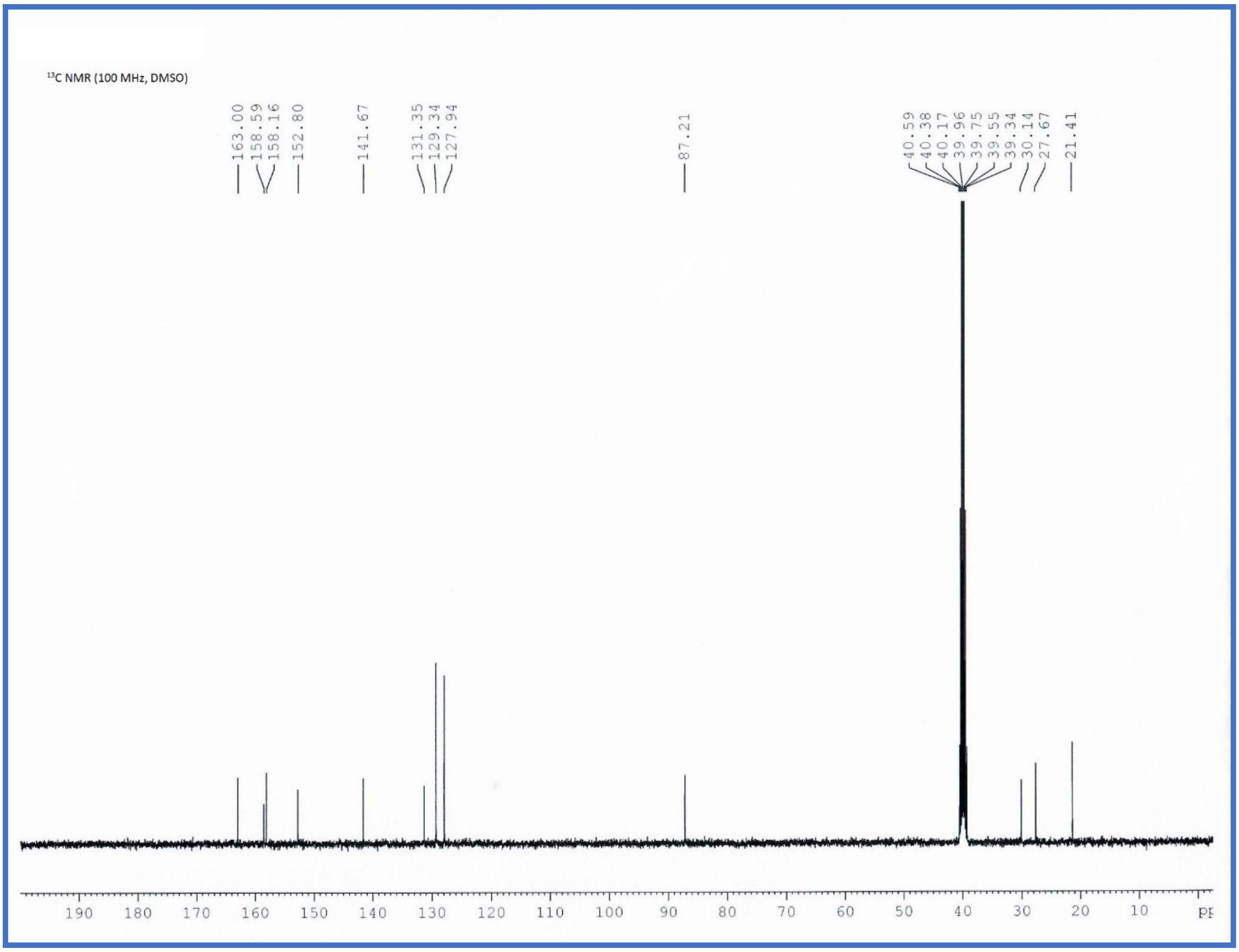

13C NMR (100 MHz, DMSO)
163.00
158.59
158.16
152.80
141.67
131.35
129.34
127.94
87.21
40.59
40.38
40.17
39.96
39.75
39.55
39.34
30.14
27.67
21.41

4-Fluro-*N*-(1,3-dimethyl-2,6-dioxo-1,2,3,6-tetrahydropyrimidin-4-yl)benzimidamide (1c):

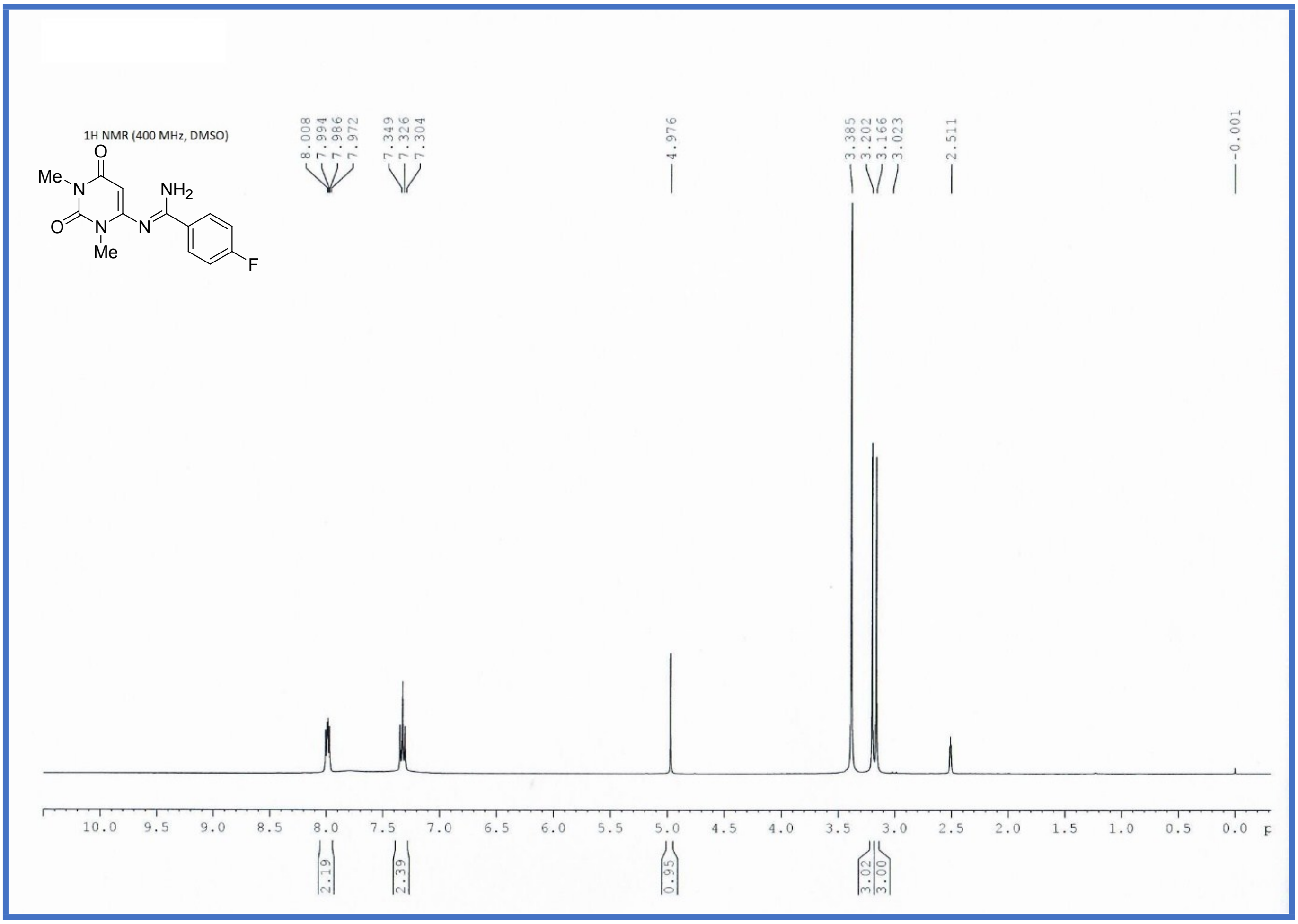

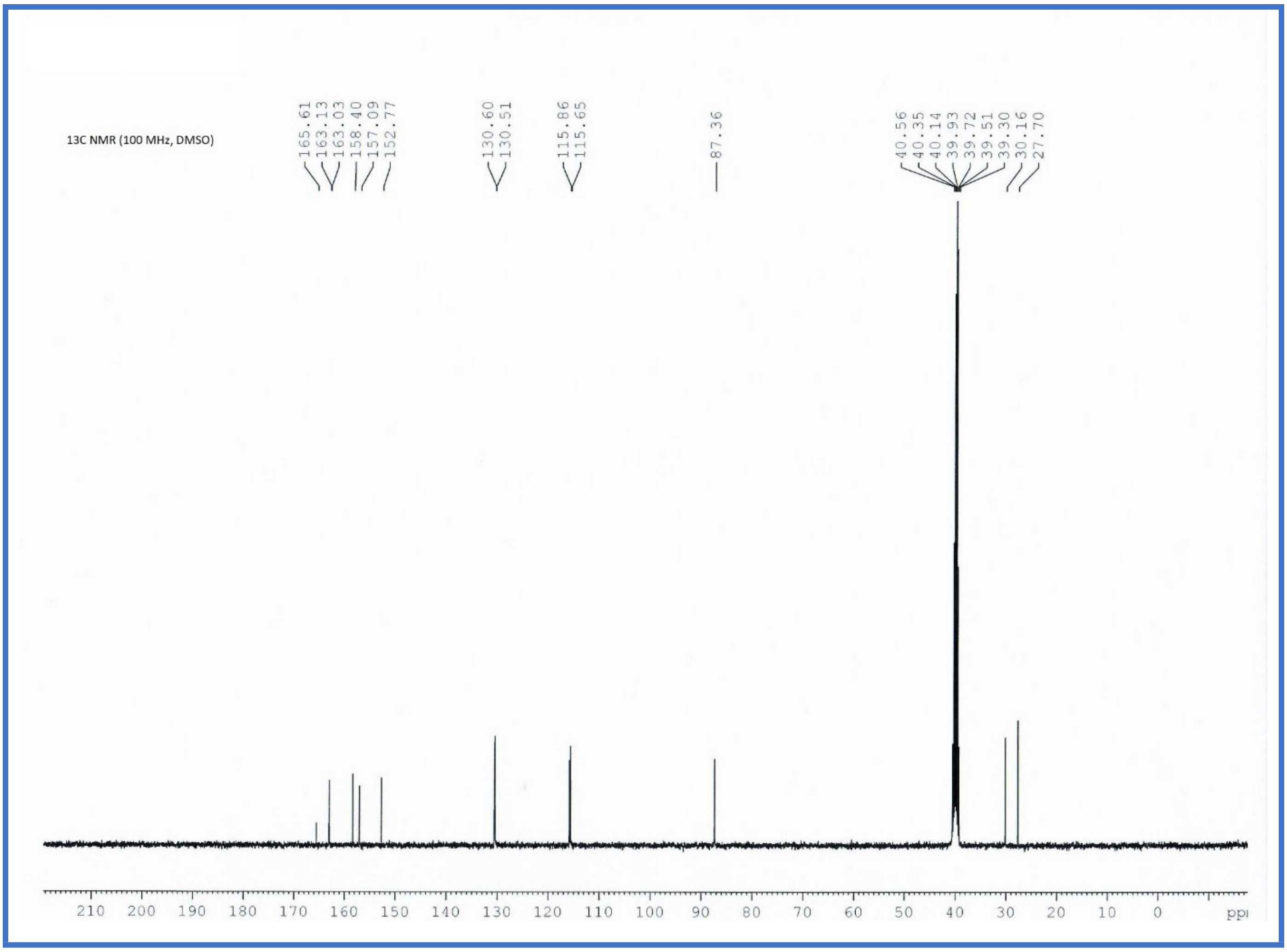
13C NMR (100 MHz, DMSO)
165.61
163.13
163.03
158.40
157.09
152.77
130.60
130.51
115.86
115.65
87.36
40.56
40.35
40.14
39.93
39.72
39.51
39.30
30.16
27.70
210 200 190 180 170 160 150 140 130 120 110 100 90 80 70 60 50 40 30 20 10 0 ppm

4-Chloro-*N*-(1,3-dimethyl-2,6-dioxo-1,2,3,6-tetrahydropyrimidin-4-yl)benzimidamide (1d):

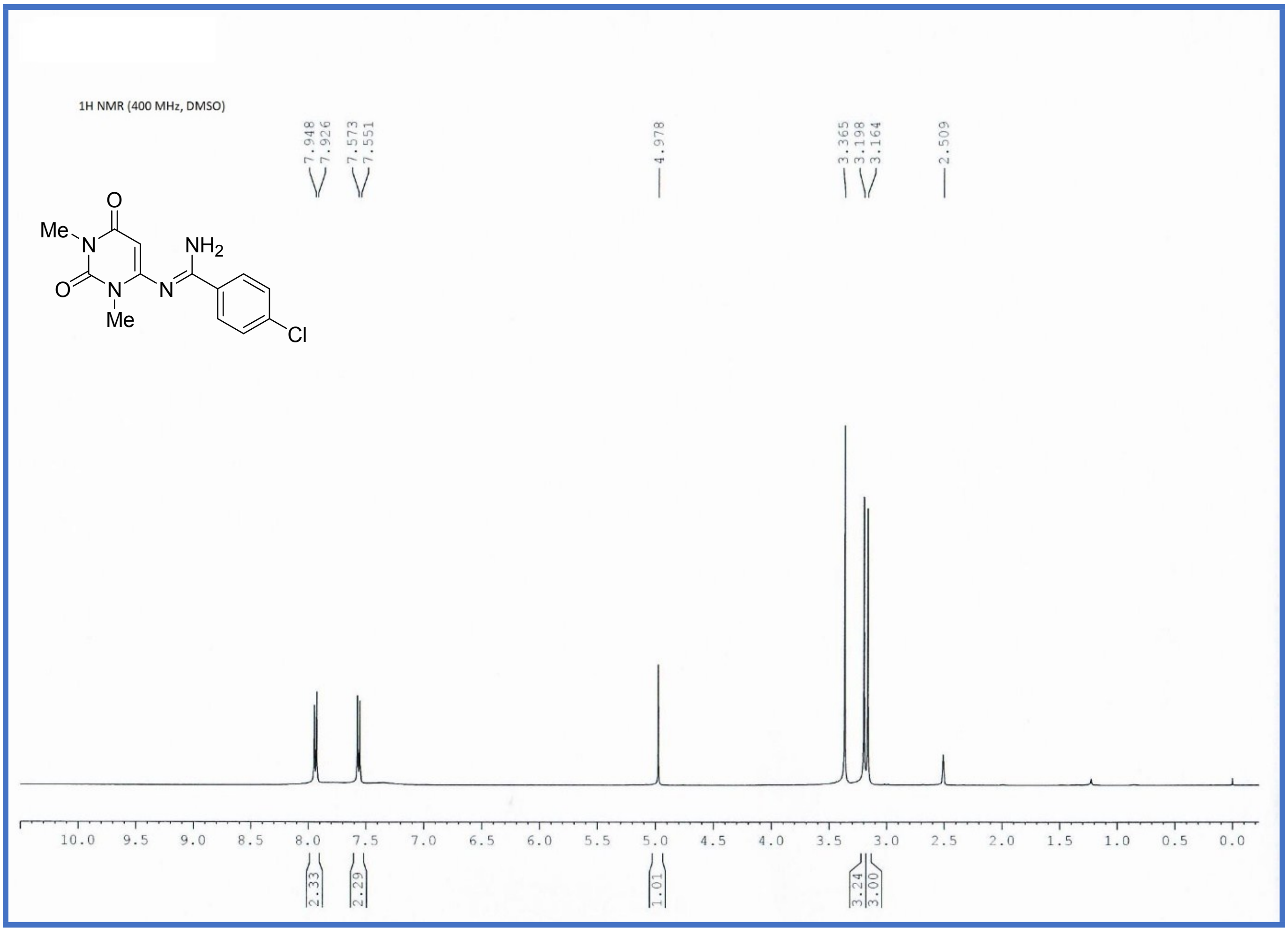

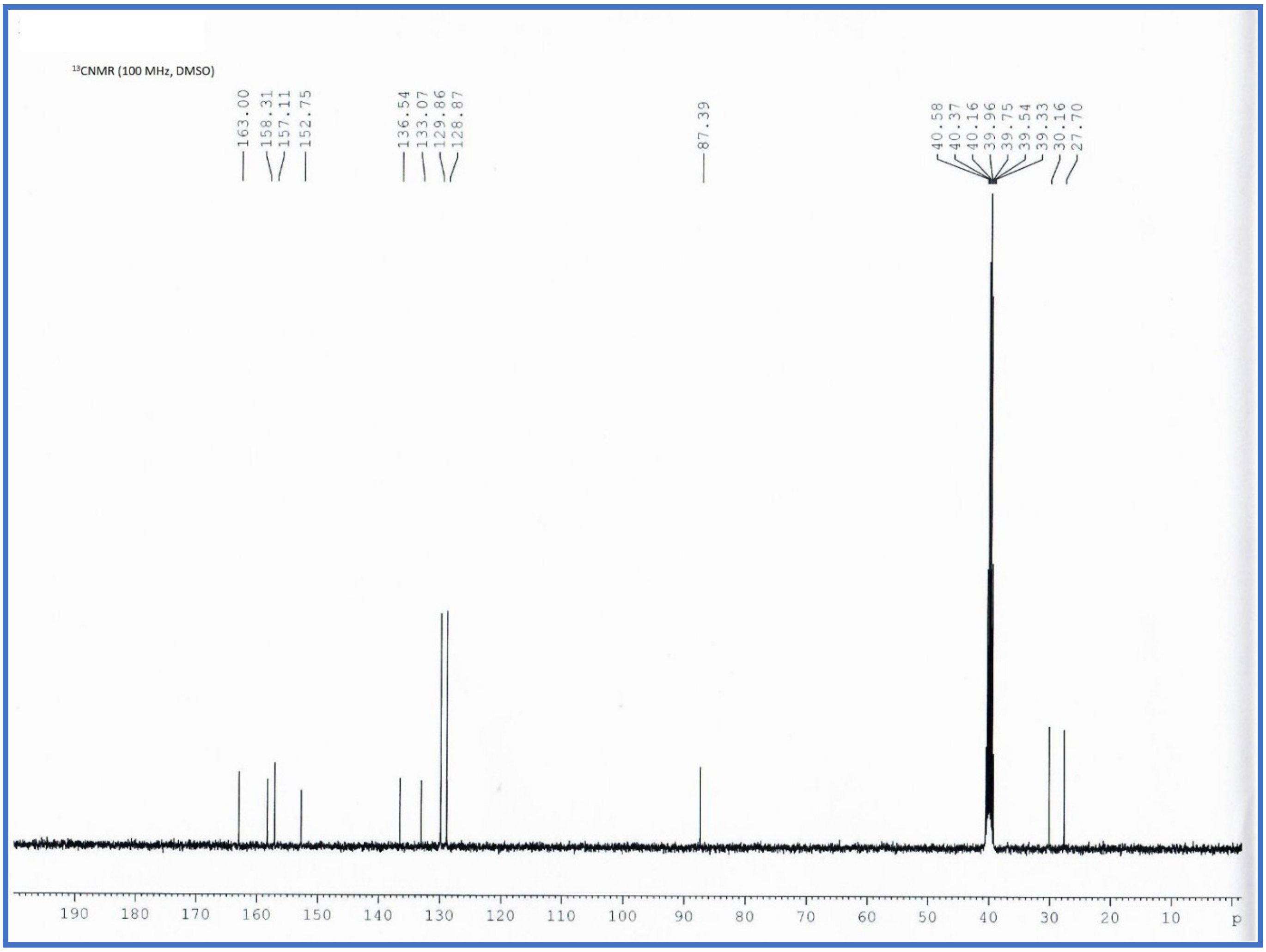

¹³CNMR (100 MHz, DMSO)
163.00
158.31
157.11
152.75
136.54
133.07
129.86
128.87
87.39
40.58
40.37
40.16
39.96
39.75
39.54
39.33
30.16
27.70
190 180 170 160 150 140 130 120 110 100 90 80 70 60 50 40 30 20 10 p

4-Bromo-*N*-(1,3-dimethyl-2,6-dioxo-1,2,3,6-tetrahydropyrimidin-4-yl)benzimidamide (1e):

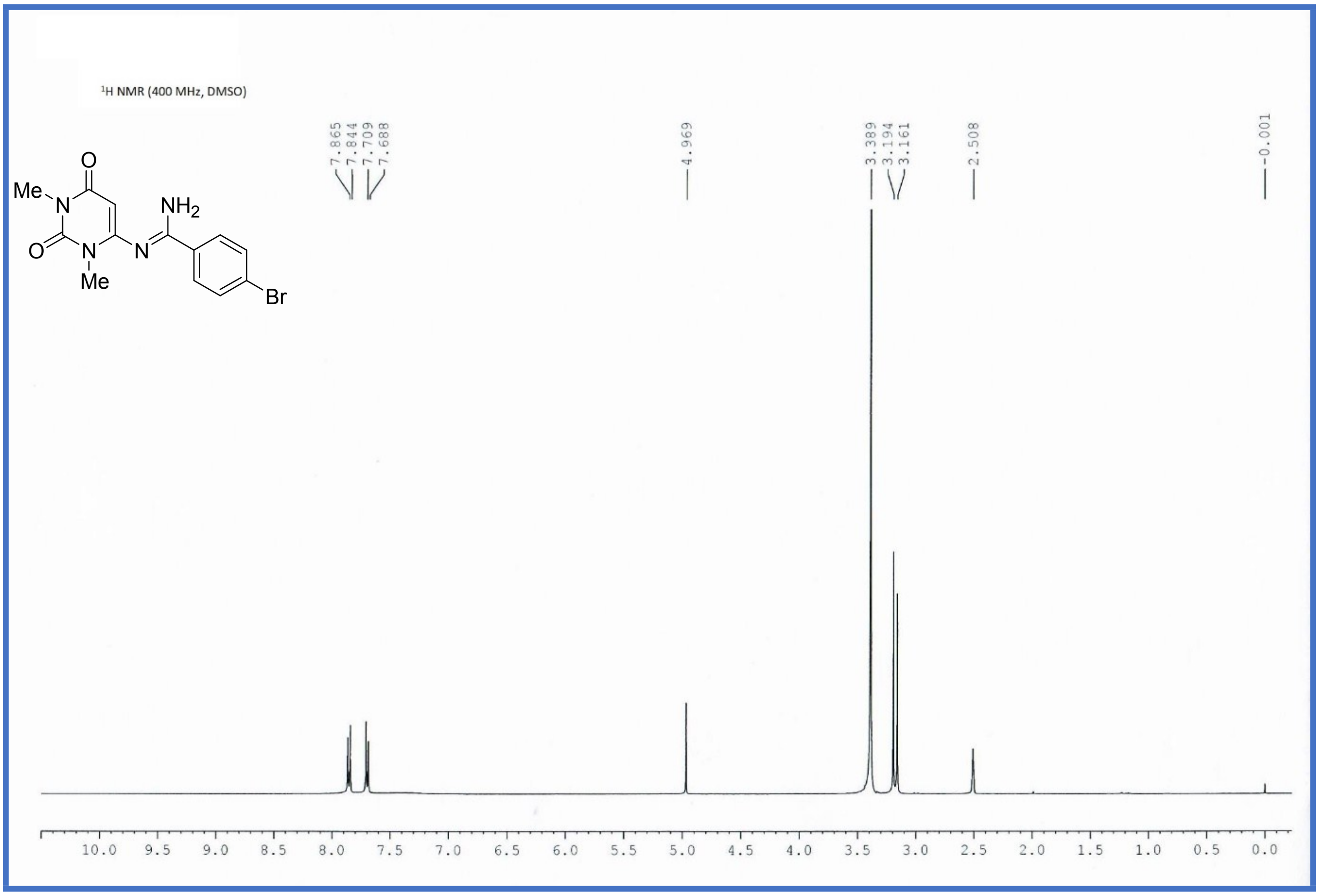

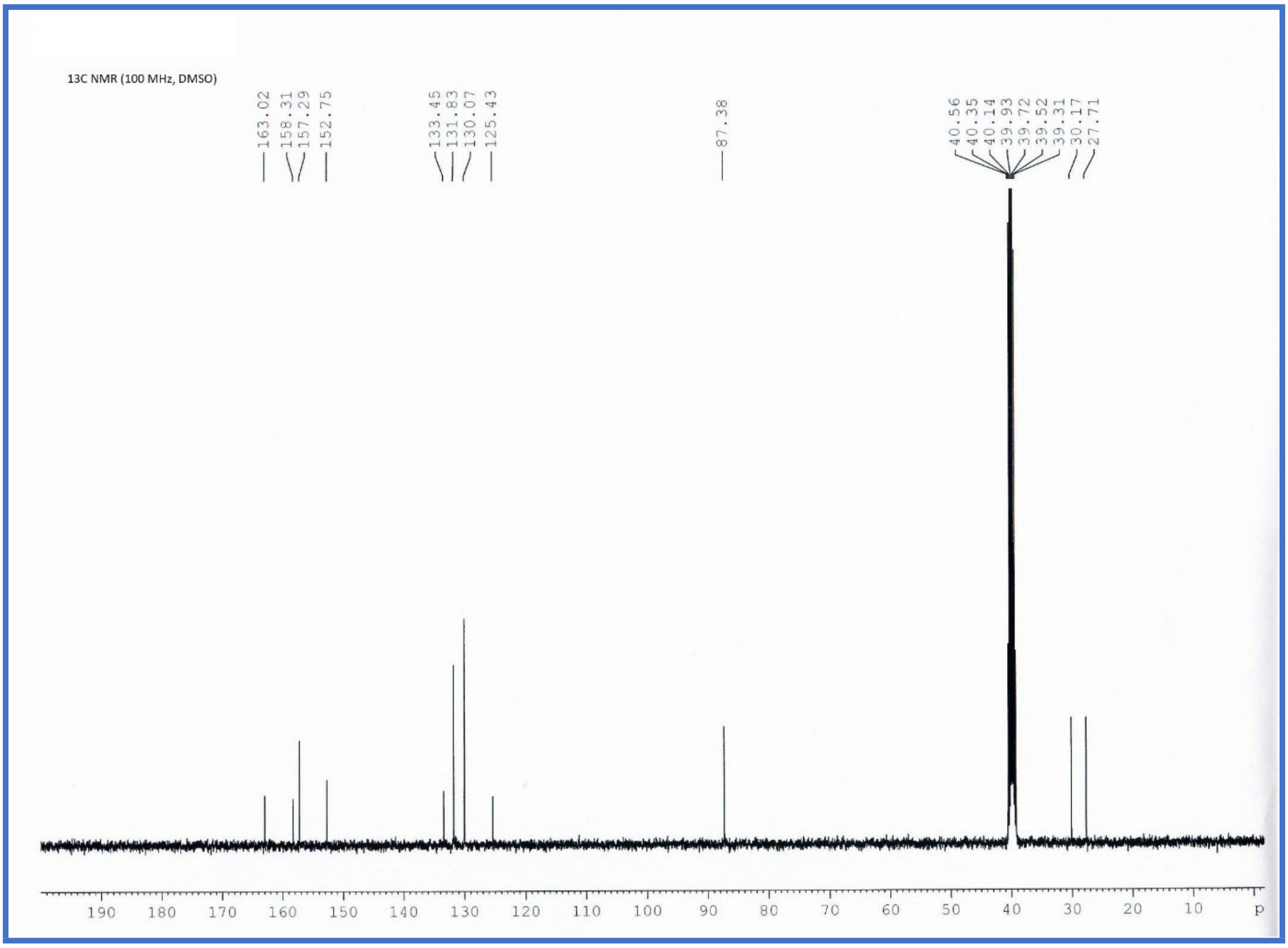
13C NMR (100 MHz, DMSO)
163.02
158.31
157.29
152.75
133.45
131.83
130.07
125.43
87.38
40.56
40.35
40.14
39.93
39.72
39.52
39.31
30.17
27.71
190
180
170
160
150
140
130
120
110
100
90
80
70
60
50
40
30
20
10
p

***N*-(3-Benzyl-1-methyl-2,6-dioxo-1,2,3,6-tetrahydropyrimidin-4-yl)benzimidamide (1f):**

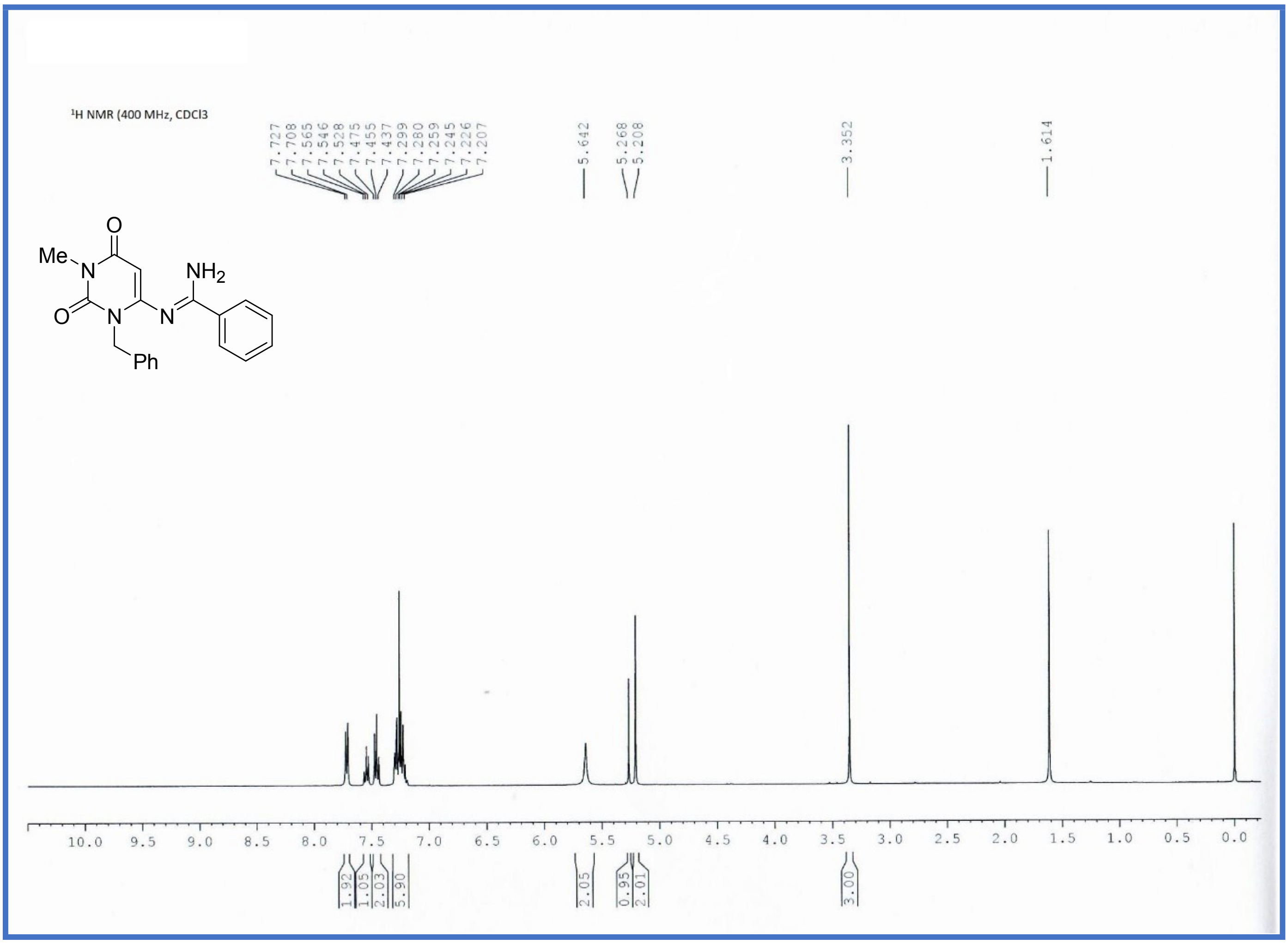

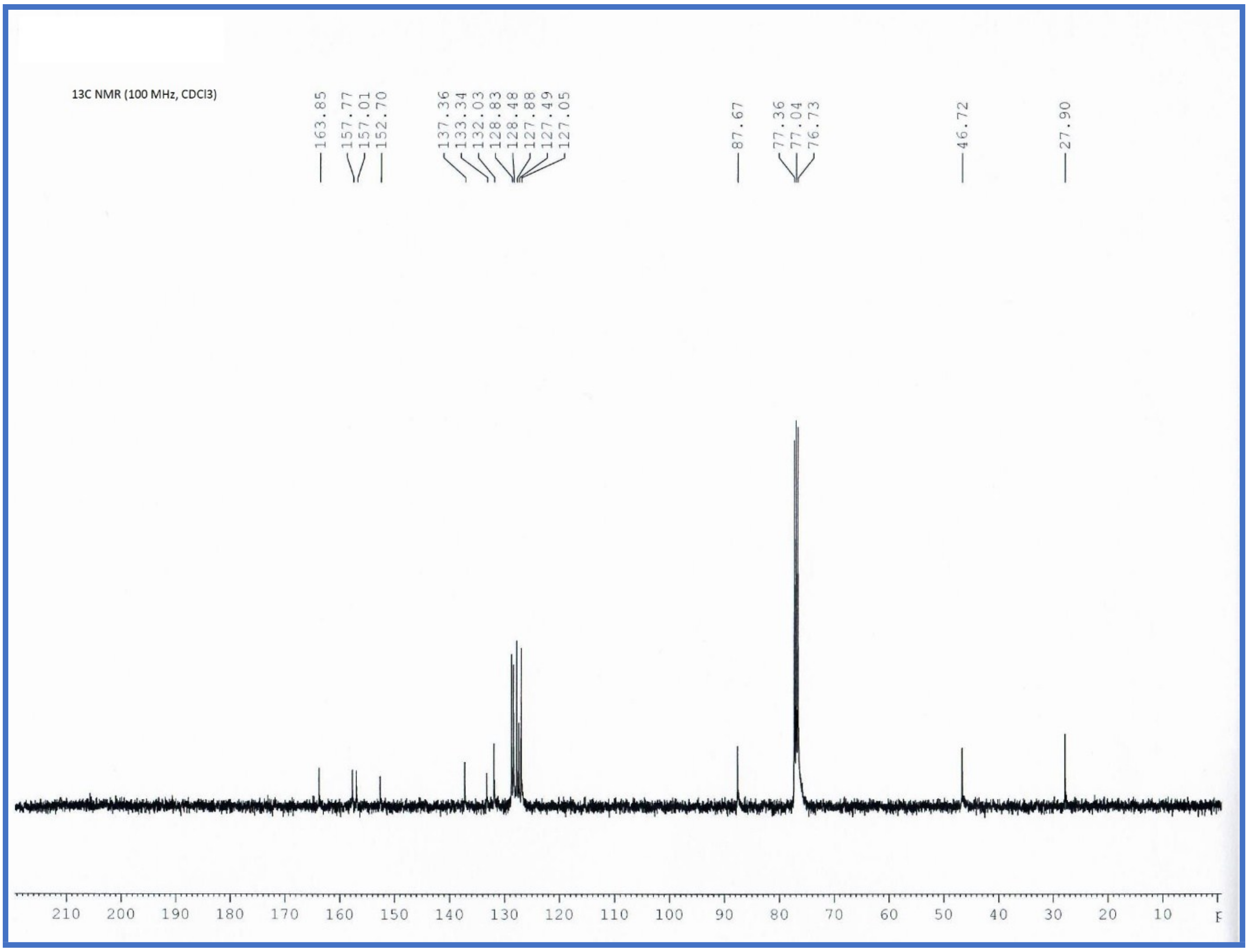
13C NMR (100 MHz, CDCl3)
163.85
157.77
157.01
152.70
137.36
133.34
132.03
128.83
128.48
127.88
127.49
127.05
87.67
77.36
77.04
76.73
46.72
27.90
210 200 190 180 170 160 150 140 130 120 110 100 90 80 70 60 50 40 30 20 10

***N*-(1,3-Dibenzyl-2,6-dioxo-1,2,3,6-tetrahydropyrimidin-4-yl)benzimidamide (1g):**

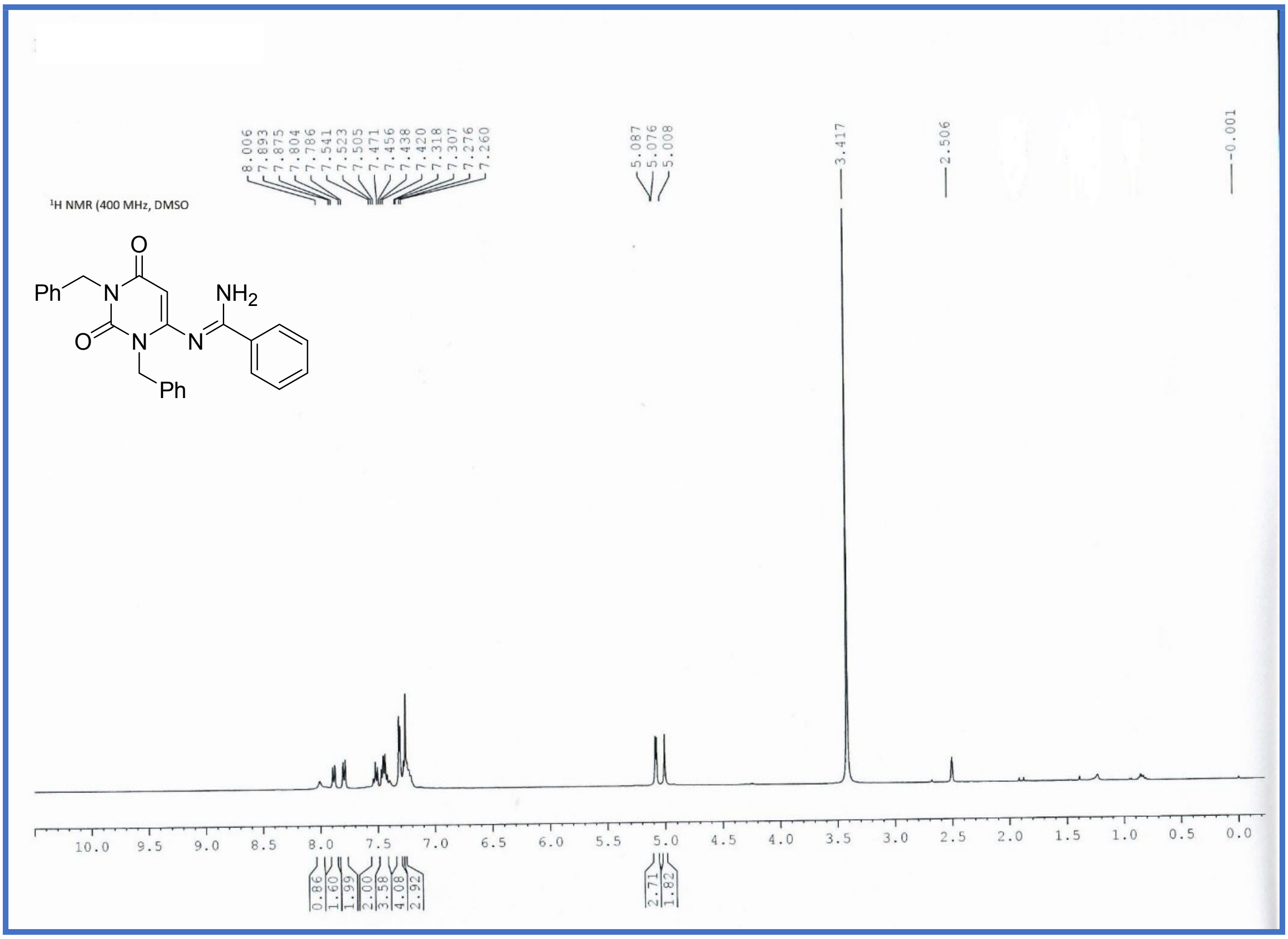

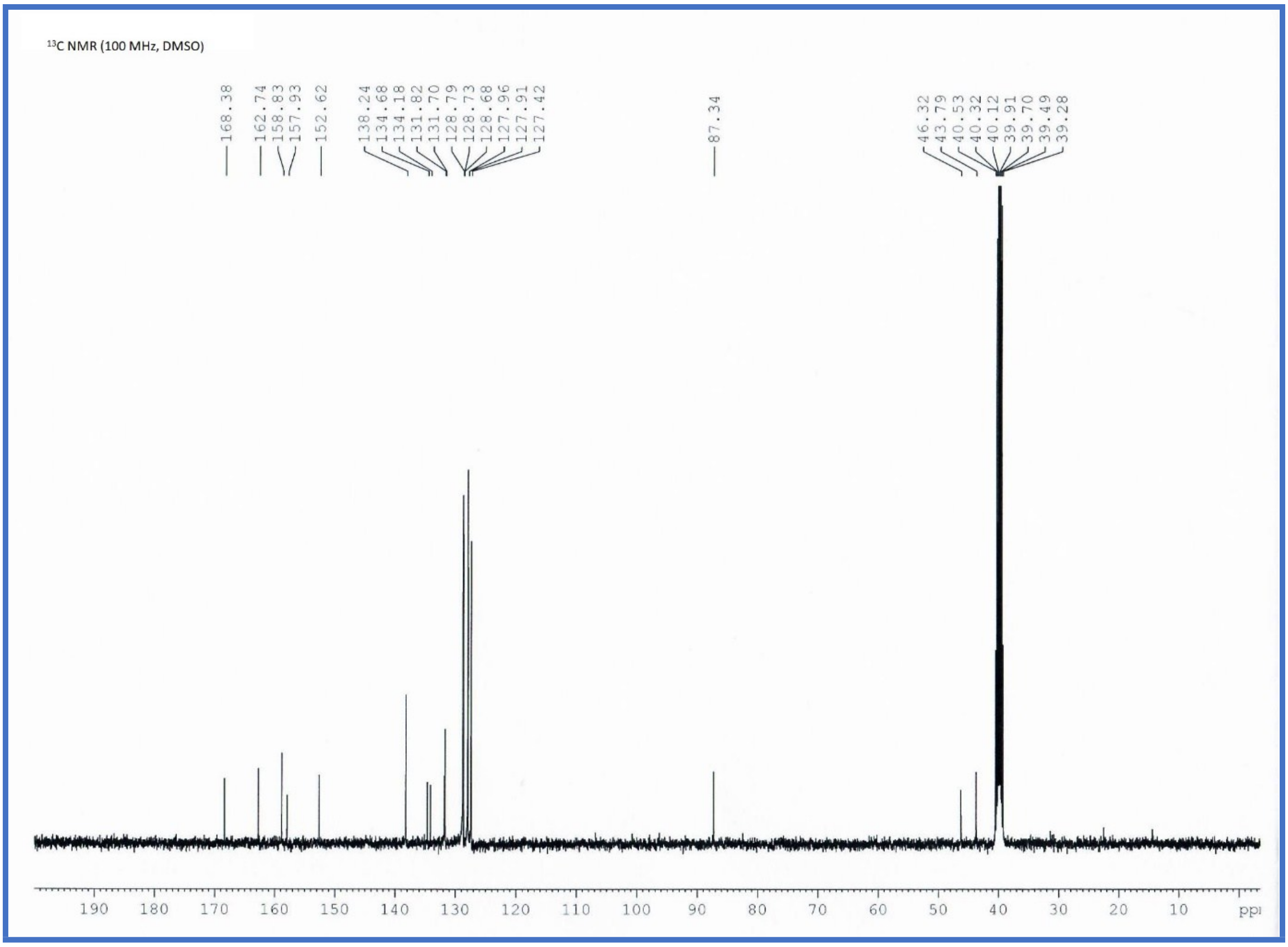

13C NMR (100 MHz, DMSO)
168.38
162.74
158.83
157.93
152.62
138.24
134.68
134.18
131.82
131.70
128.79
128.73
128.68
127.96
127.91
127.42
87.34
46.32
43.79
40.53
40.32
40.12
39.91
39.70
39.49
39.28
190 180 170 160 150 140 130 120 110 100 90 80 70 60 50 40 30 20 10 ppm

***N*-(1,3-Dimethyl-2,6-dioxo-1,2,3,6-tetrahydropyrimidin-4-yl)ethanimidamide (1h):**

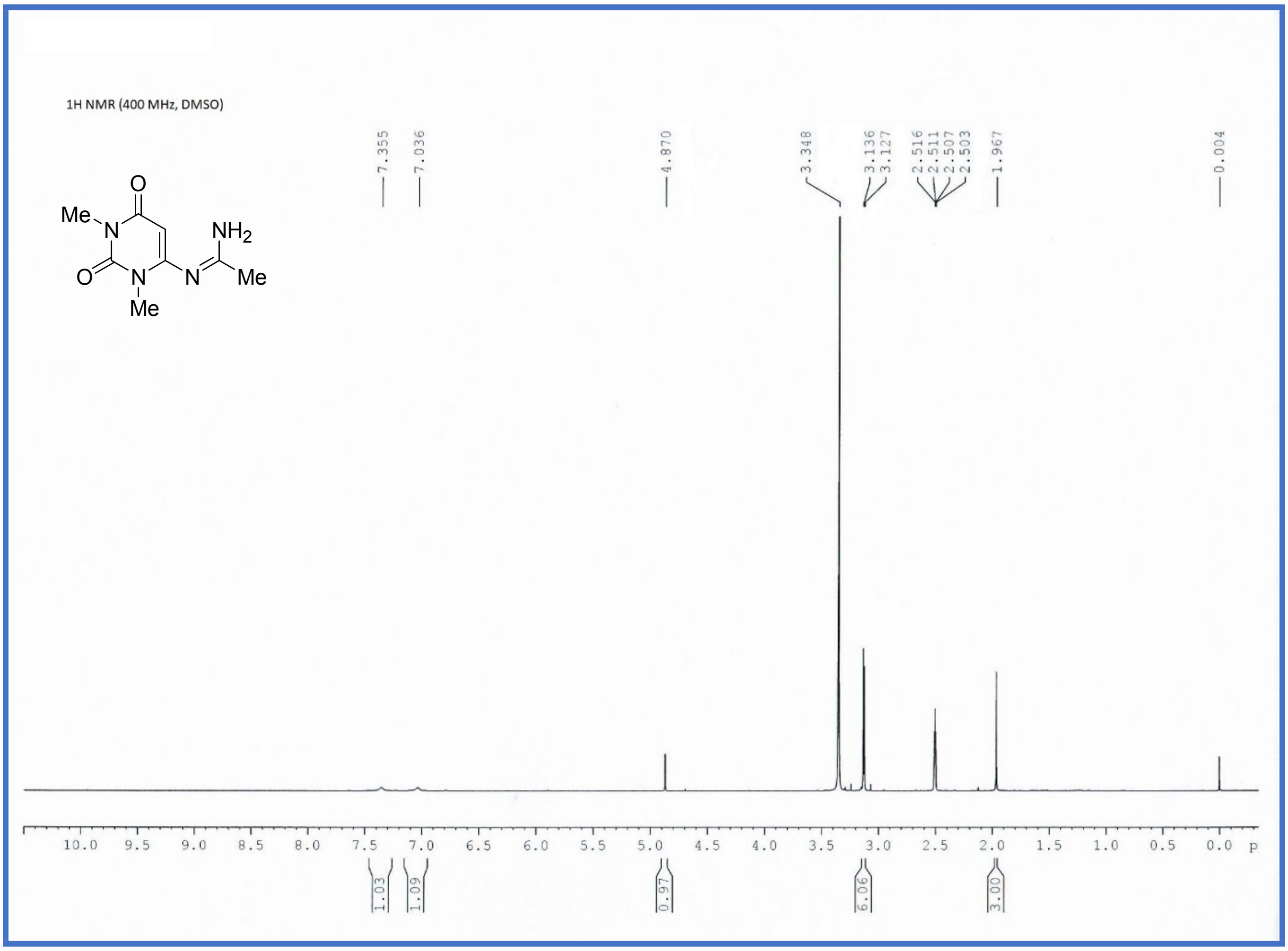

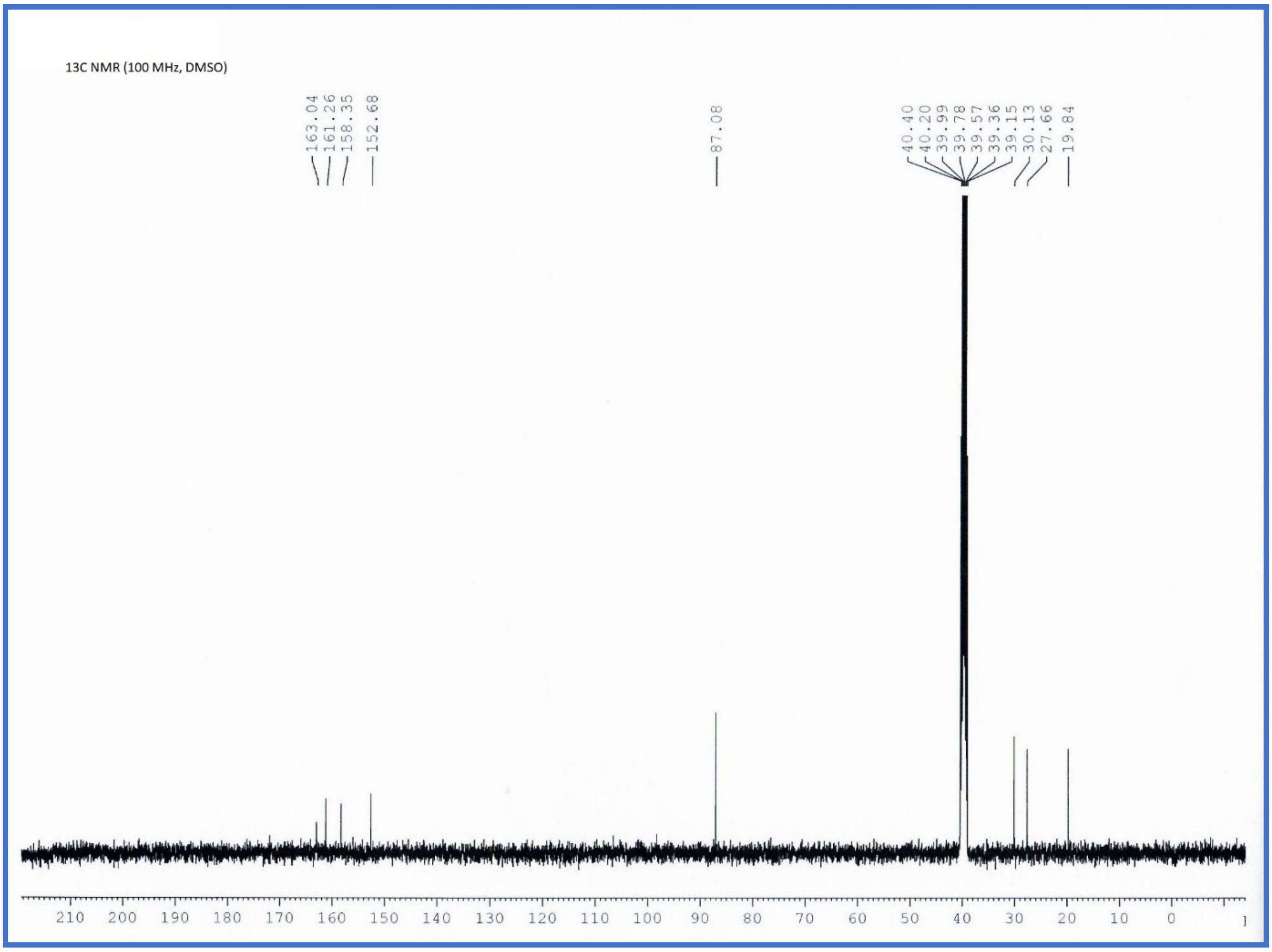
13C NMR (100 MHz, DMSO)
163.04
161.26
158.35
152.68
87.08
40.40
40.20
39.99
39.78
39.57
39.36
39.15
30.13
27.66
19.84
210 200 190 180 170 160 150 140 130 120 110 100 90 80 70 60 50 40 30 20 10 0

1,3-Dimethyl-5,7-diphenylpyrimido[4,5-*d*]pyrimidine-2,4(1*H*,3*H*)-dione (3a):

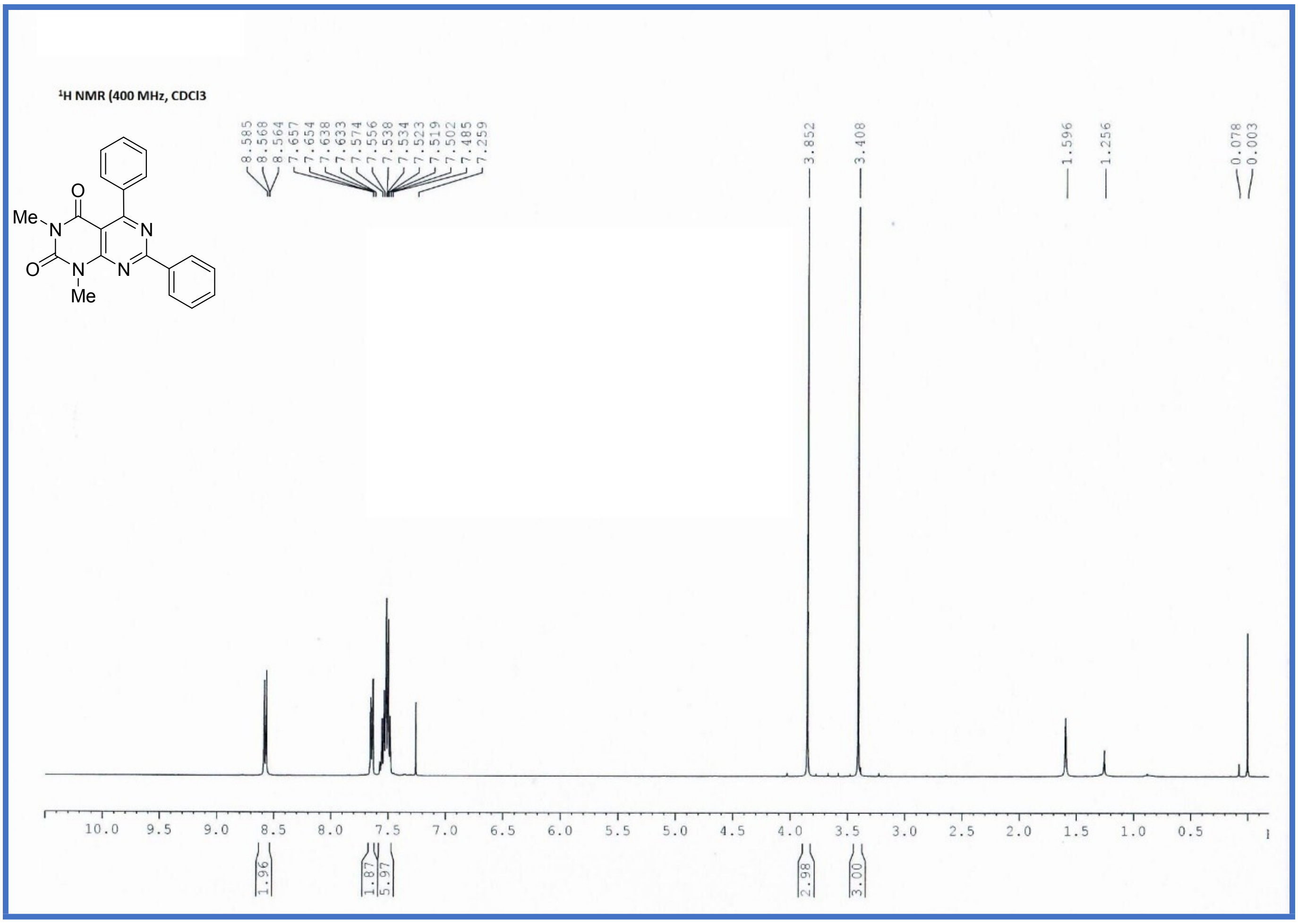

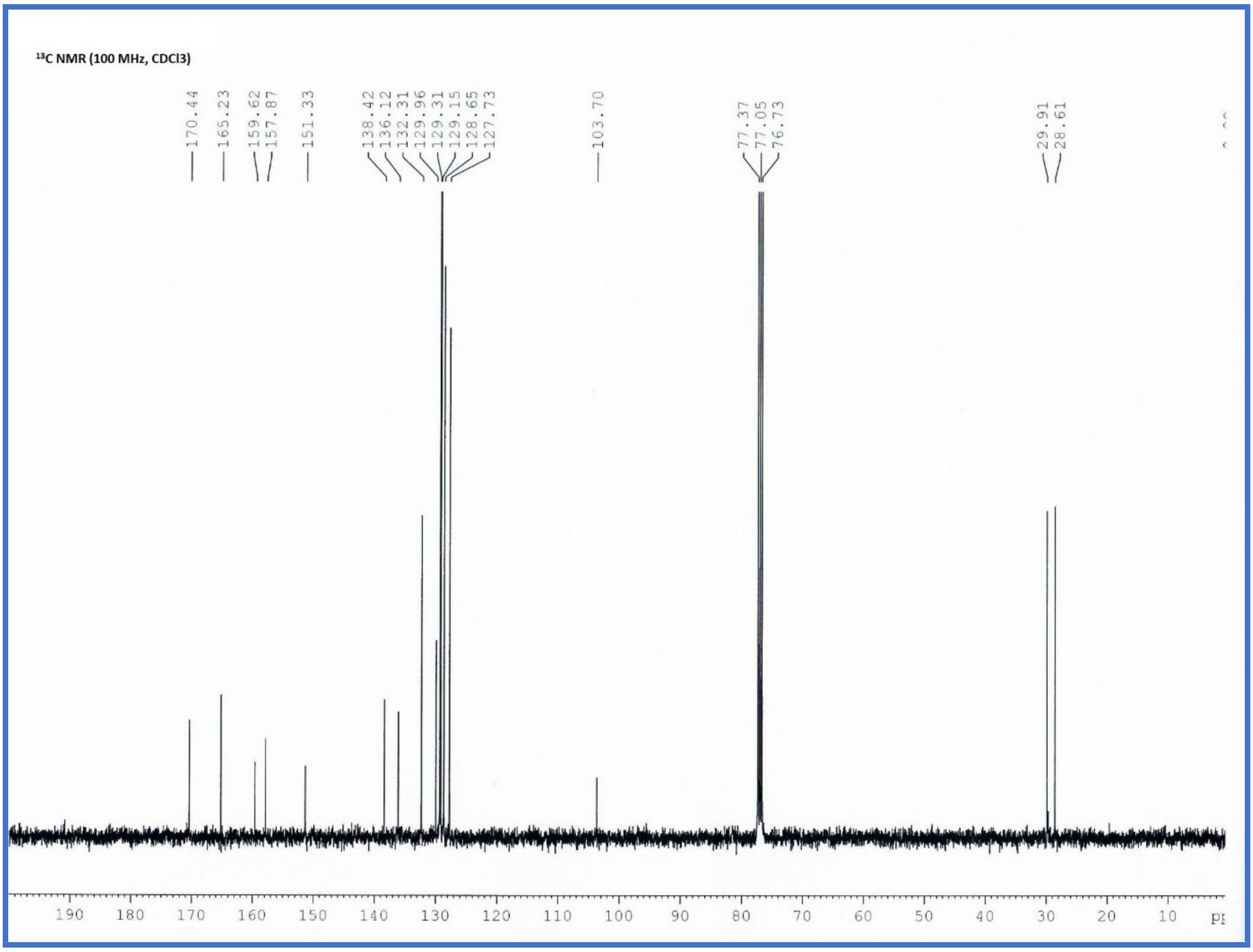

¹³C NMR (100 MHz, CDCl3)
170.44
165.23
159.62
157.87
151.33
138.42
136.12
132.31
129.96
129.31
129.15
128.65
127.73
103.70
77.37
77.05
76.73
29.91
28.61
190 180 170 160 150 140 130 120 110 100 90 80 70 60 50 40 30 20 10

5-(4-Methoxyphenyl)-1,3-dimethyl-7-phenylpyrimido[4,5-*d*]pyrimidine-2,4(1*H*,3*H*)-dione (3b):

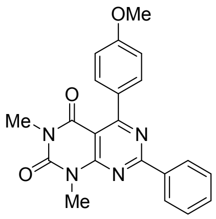

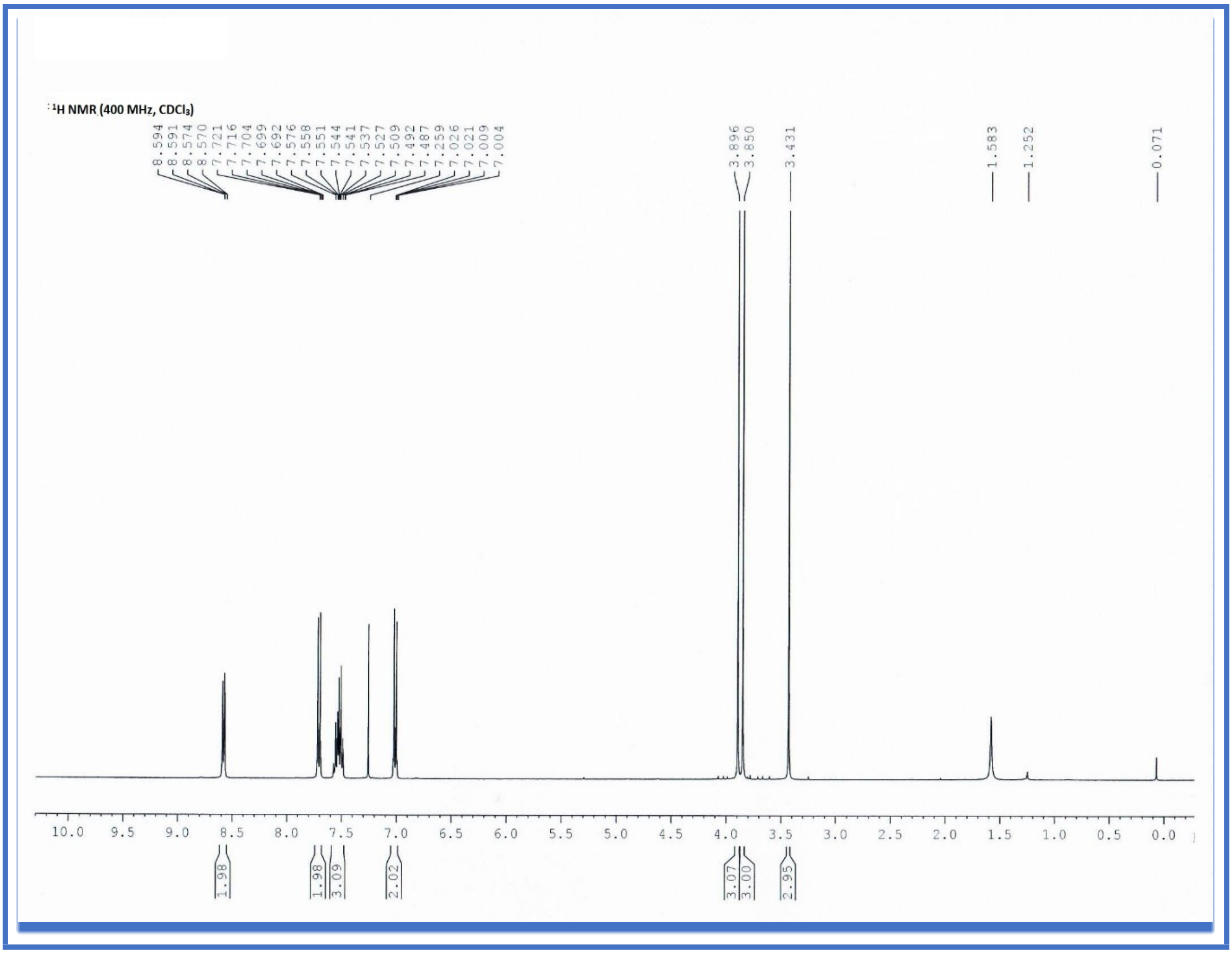

1H NMR (400 MHz, CDCl3)
8.594
8.591
8.574
8.570
7.721
7.716
7.704
7.699
7.692
7.576
7.558
7.551
7.544
7.541
7.537
7.527
7.509
7.492
7.487
7.259
7.026
7.021
7.009
7.004
3.896
3.850
3.431
1.583
1.252
0.071
10.0 9.5 9.0 8.5 8.0 7.5 7.0 6.5 6.0 5.5 5.0 4.5 4.0 3.5 3.0 2.5 2.0 1.5 1.0 0.5 0.0
1.98
1.98
3.09
2.02
3.07
3.00
2.95

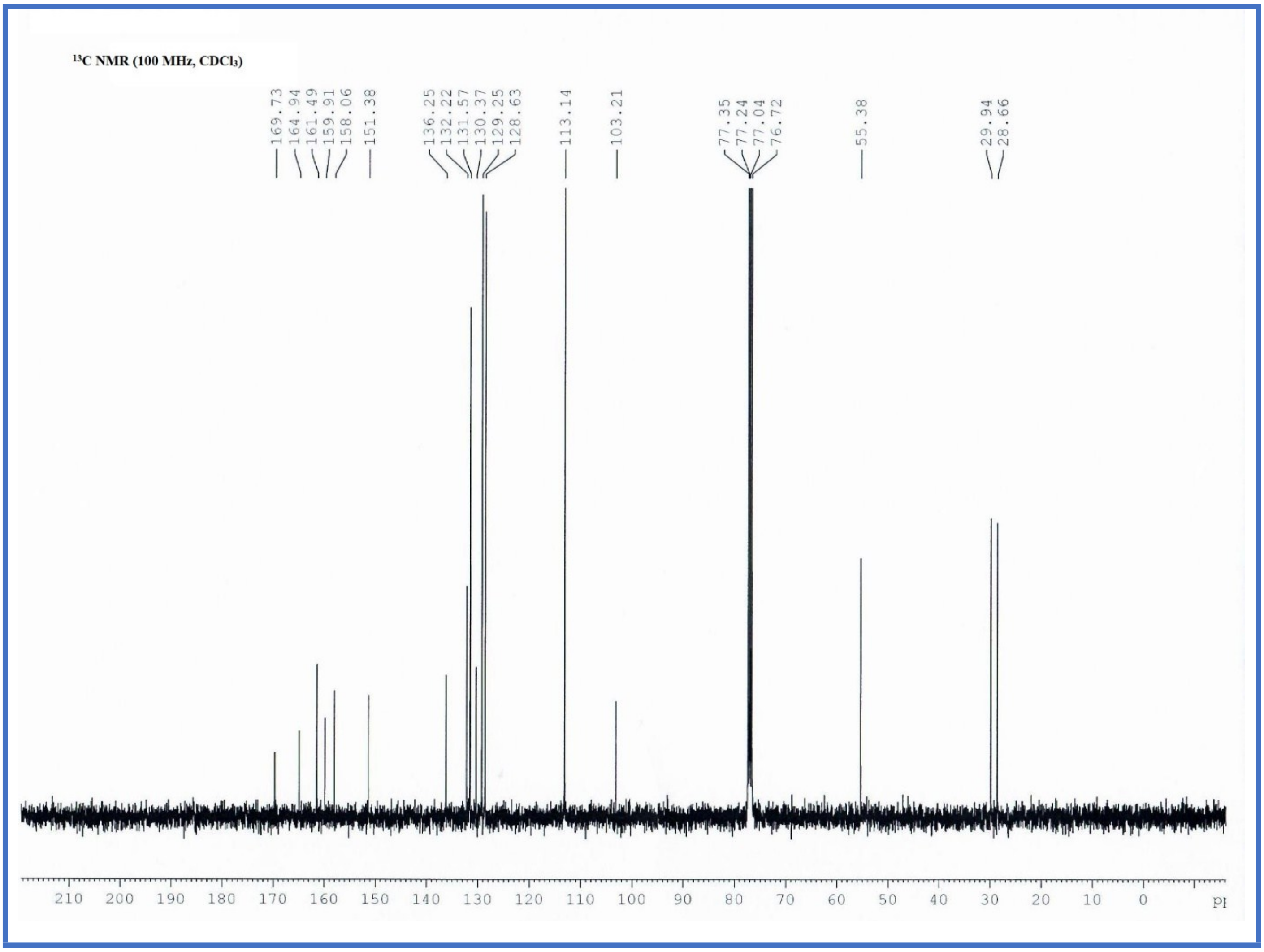

¹³C NMR (100 MHz, CDCl₃)
169.73
164.94
161.49
159.91
158.06
151.38
136.25
132.22
131.57
130.37
129.25
128.63
113.14
103.21
77.35
77.24
77.04
76.72
55.38
29.94
28.66
210 200 190 180 170 160 150 140 130 120 110 100 90 80 70 60 50 40 30 20 10 0

1,3-Dimethyl-5-(4-methylphenyl)-7-phenylpyrimido[4,5-*d*]pyrimidine-2,4(1*H*,3*H*)-dione (3c):

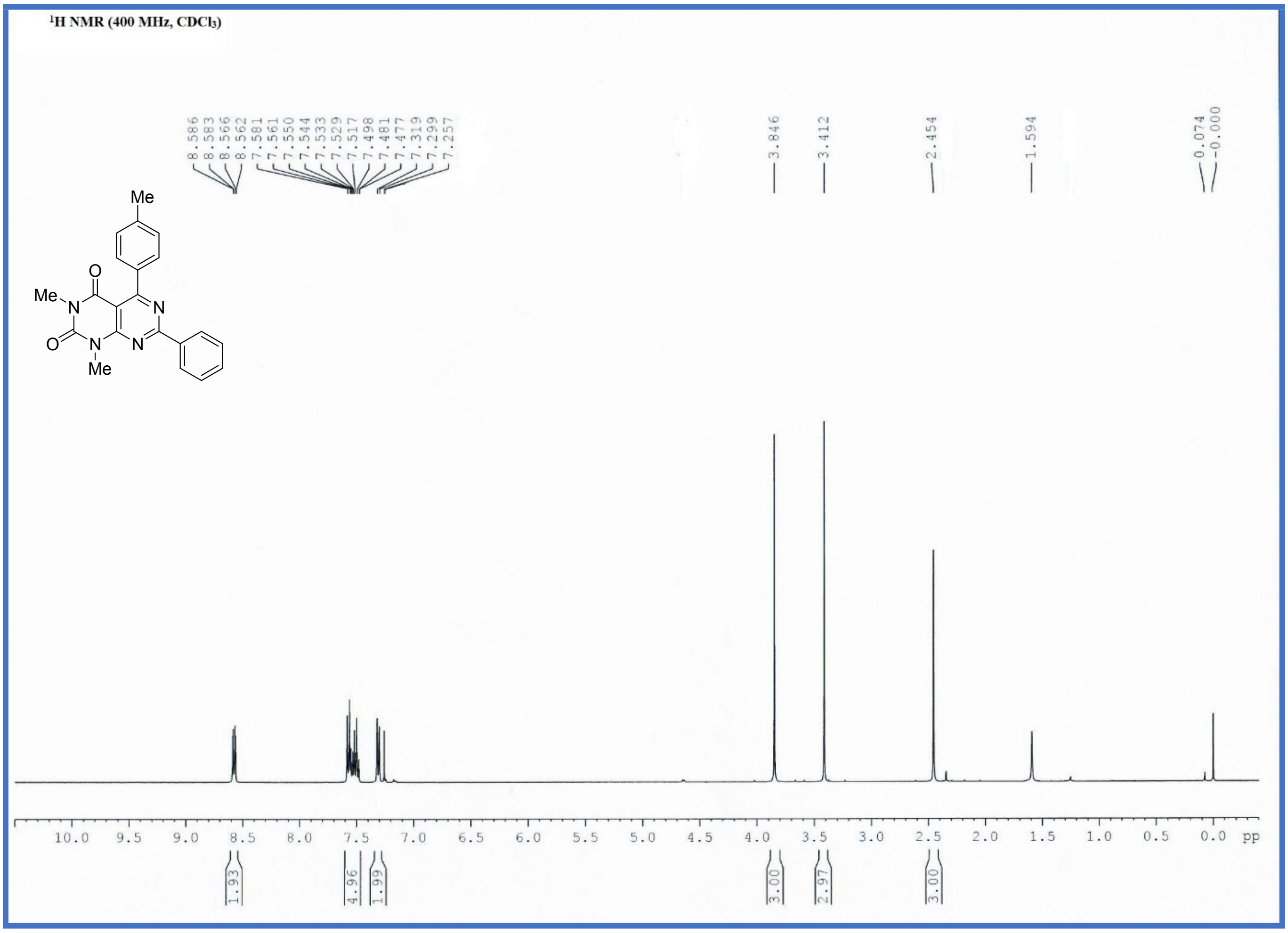

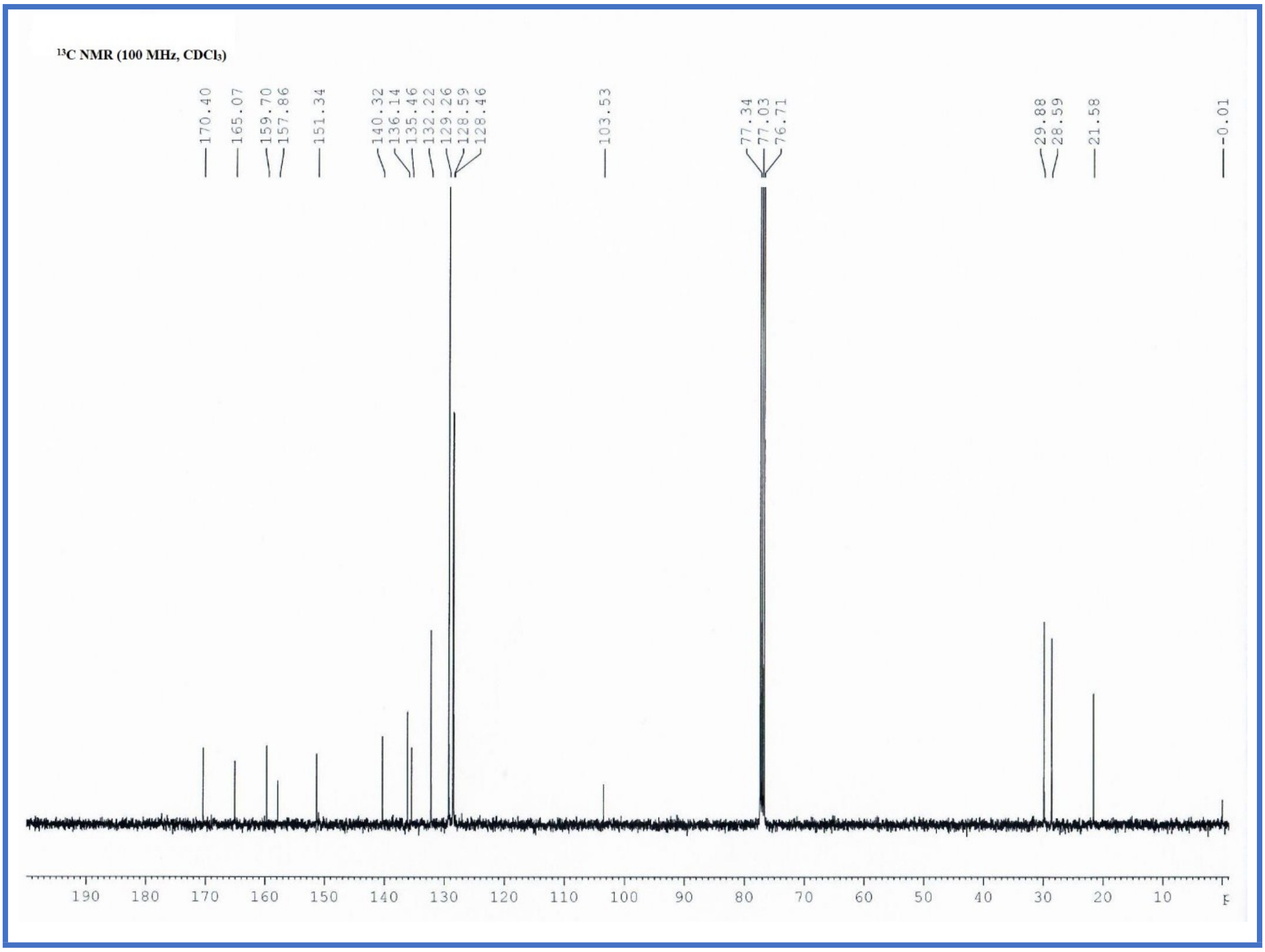

13C NMR (100 MHz, CDCl3)
170.40
165.07
159.70
157.86
151.34
140.32
136.14
135.46
132.22
129.26
128.59
128.46
103.53
77.34
77.03
76.71
29.88
28.59
21.58
-0.01
190 180 170 160 150 140 130 120 110 100 90 80 70 60 50 40 30 20 10

1,3-Dimethyl-5-(2-methylphenyl)-7-phenylpyrimido[4,5-*d*]pyrimidine-2,4(1*H*,3*H*)-dione (3d):

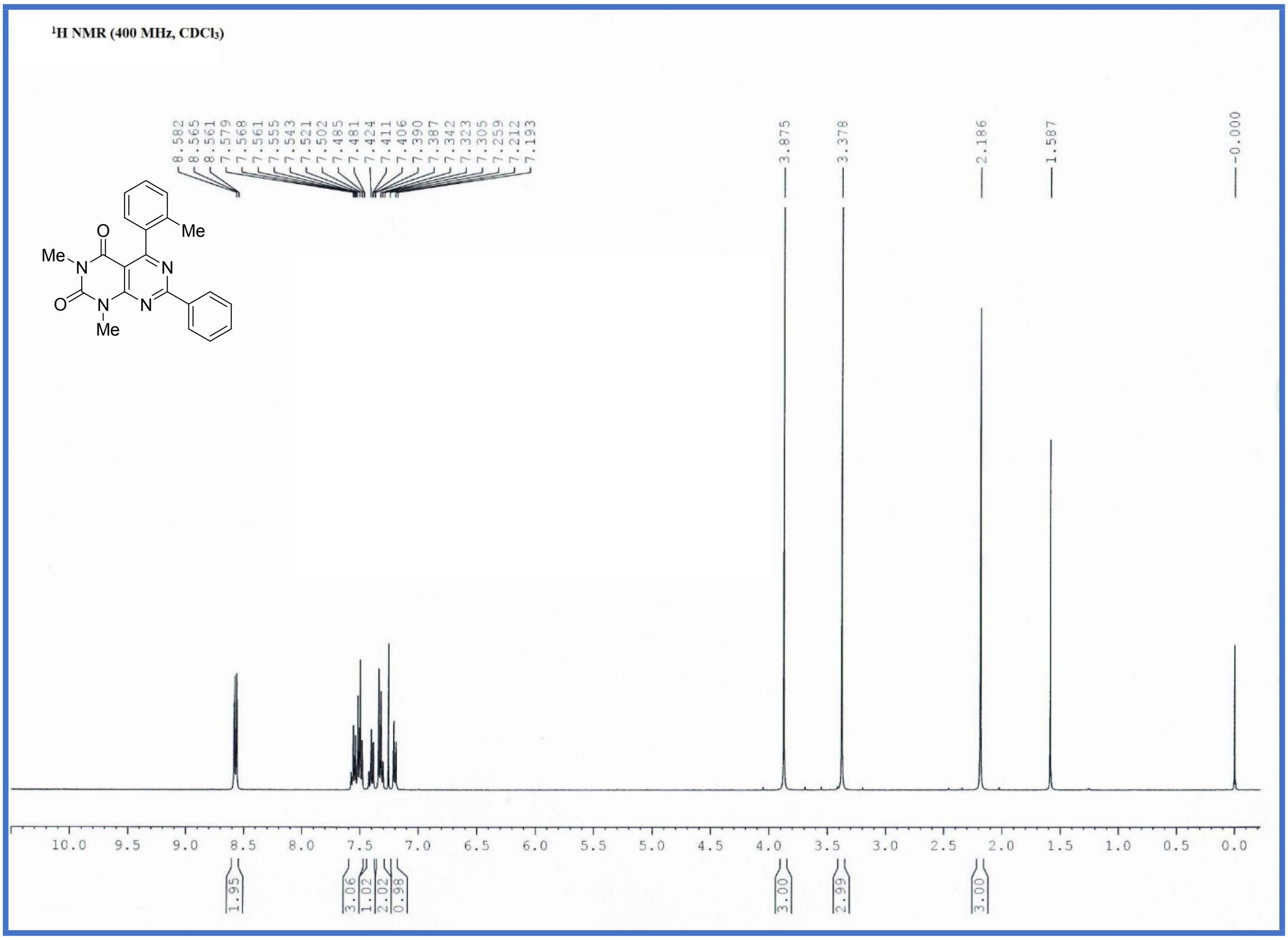

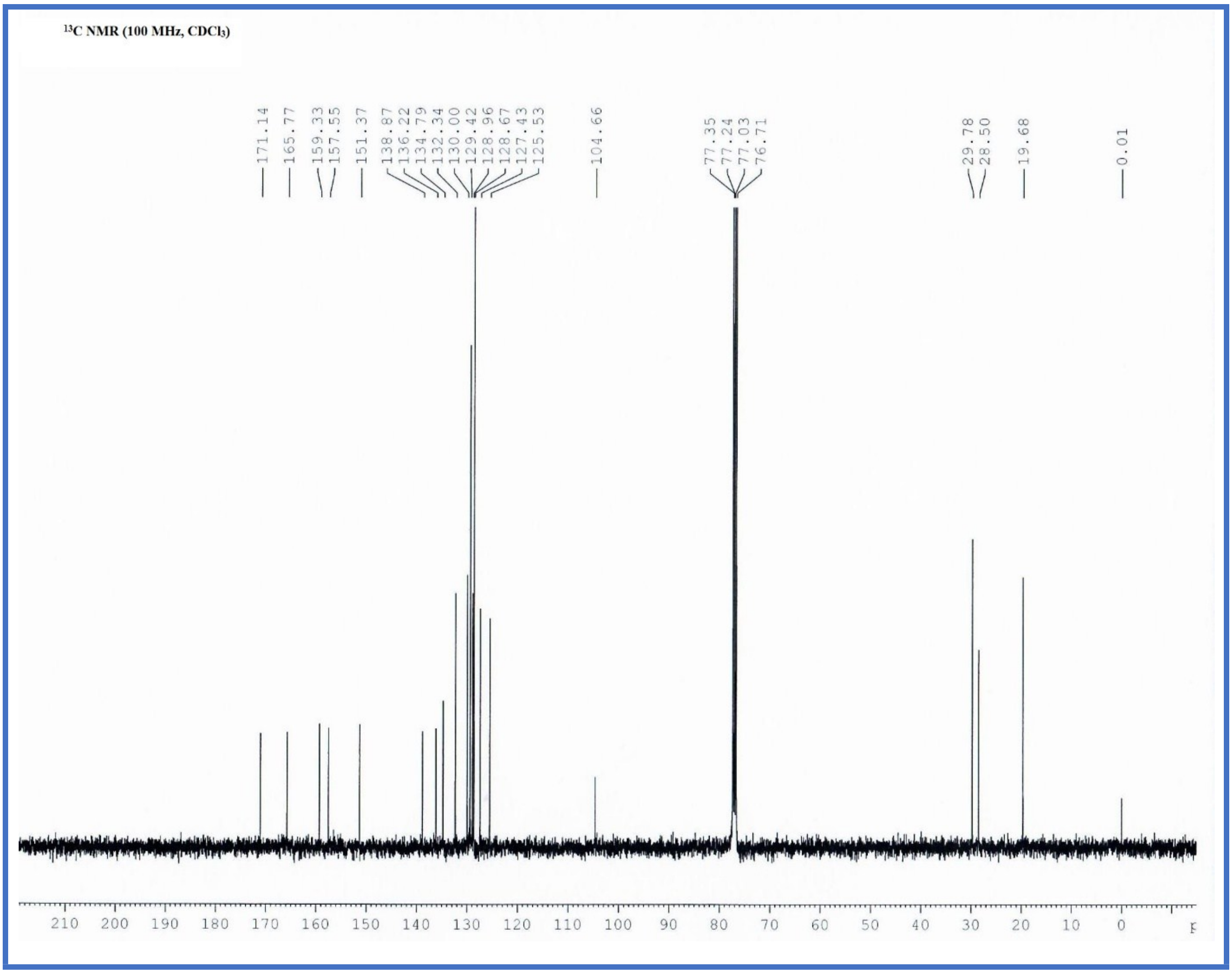

13C NMR (100 MHz, CDCl3)
171.14
165.77
159.33
157.55
151.37
138.87
136.22
134.79
132.34
130.00
129.42
128.96
128.67
127.43
125.53
104.66
77.35
77.24
77.03
76.71
29.78
28.50
19.68
0.01
210 200 190 180 170 160 150 140 130 120 110 100 90 80 70 60 50 40 30 20 10 0

1,3-Dimethyl-5-(4-thiomethylphenyl)-7-phenylpyrimido[4,5-*d*]pyrimidine-2,4(1*H*,3*H*)-dione (3e):

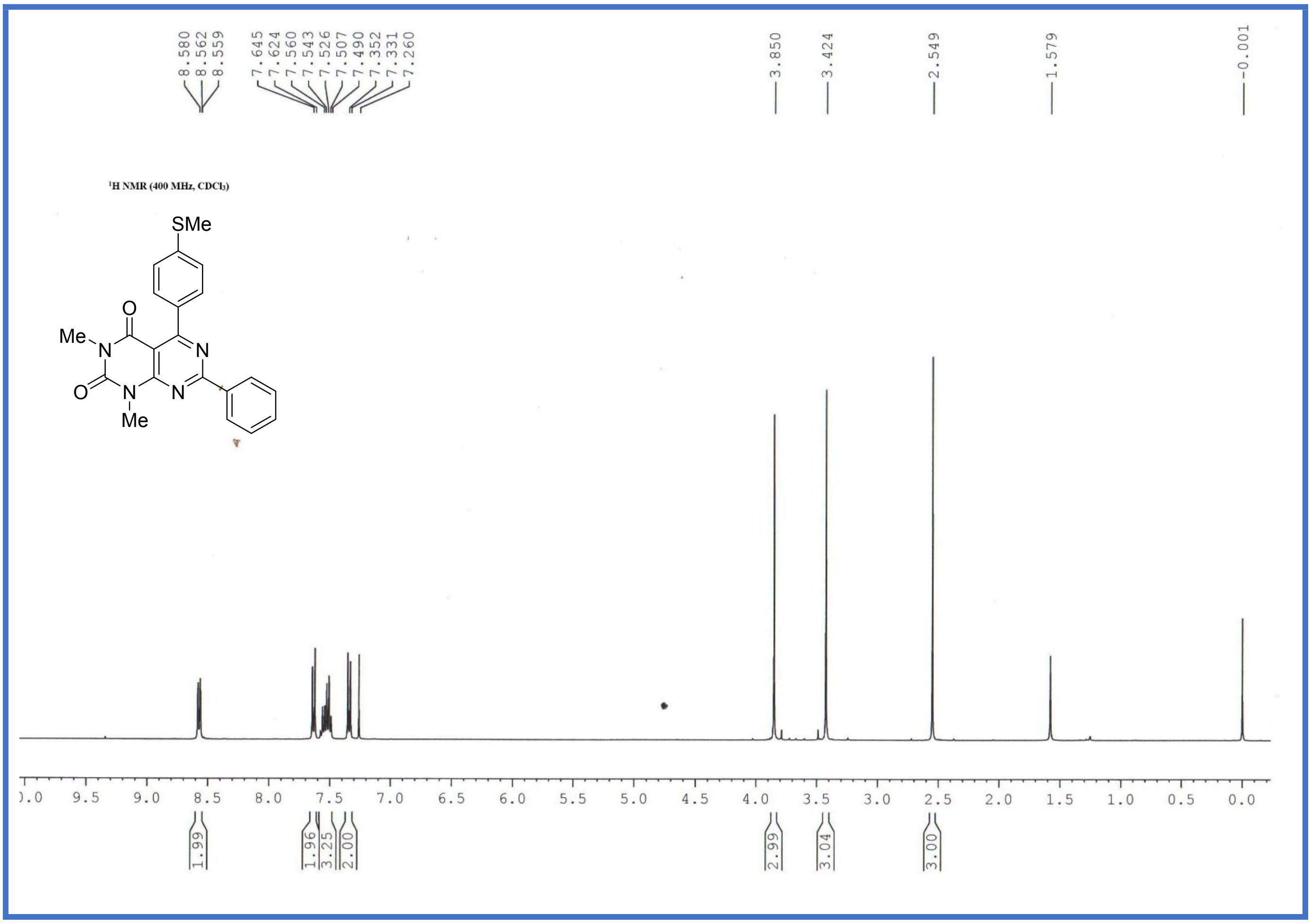

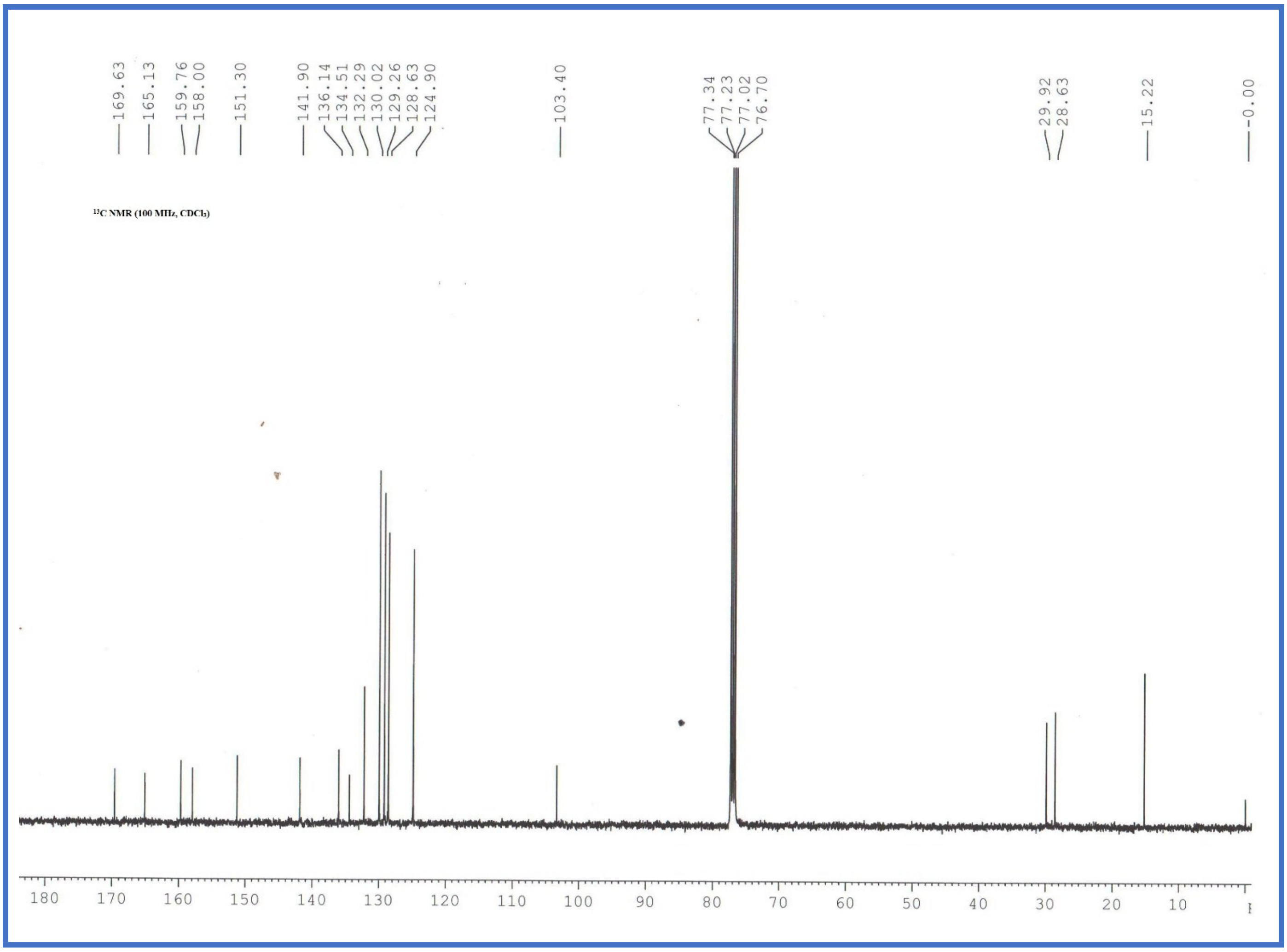
13C NMR (100 MHz, CDCl3)
169.63
165.13
159.76
158.00
151.30
141.90
136.14
134.51
132.29
130.02
129.26
128.63
124.90
103.40
77.34
77.23
77.02
76.70
29.92
28.63
15.22
-0.00
180
170
160
150
140
130
120
110
100
90
80
70
60
50
40
30
20
10

5-(4-Hydroxyphenyl)-1,3-dimethyl-7-phenylpyrimido[4,5-*d*]pyrimidine-2,4(1*H*,3*H*)-dione (3f):

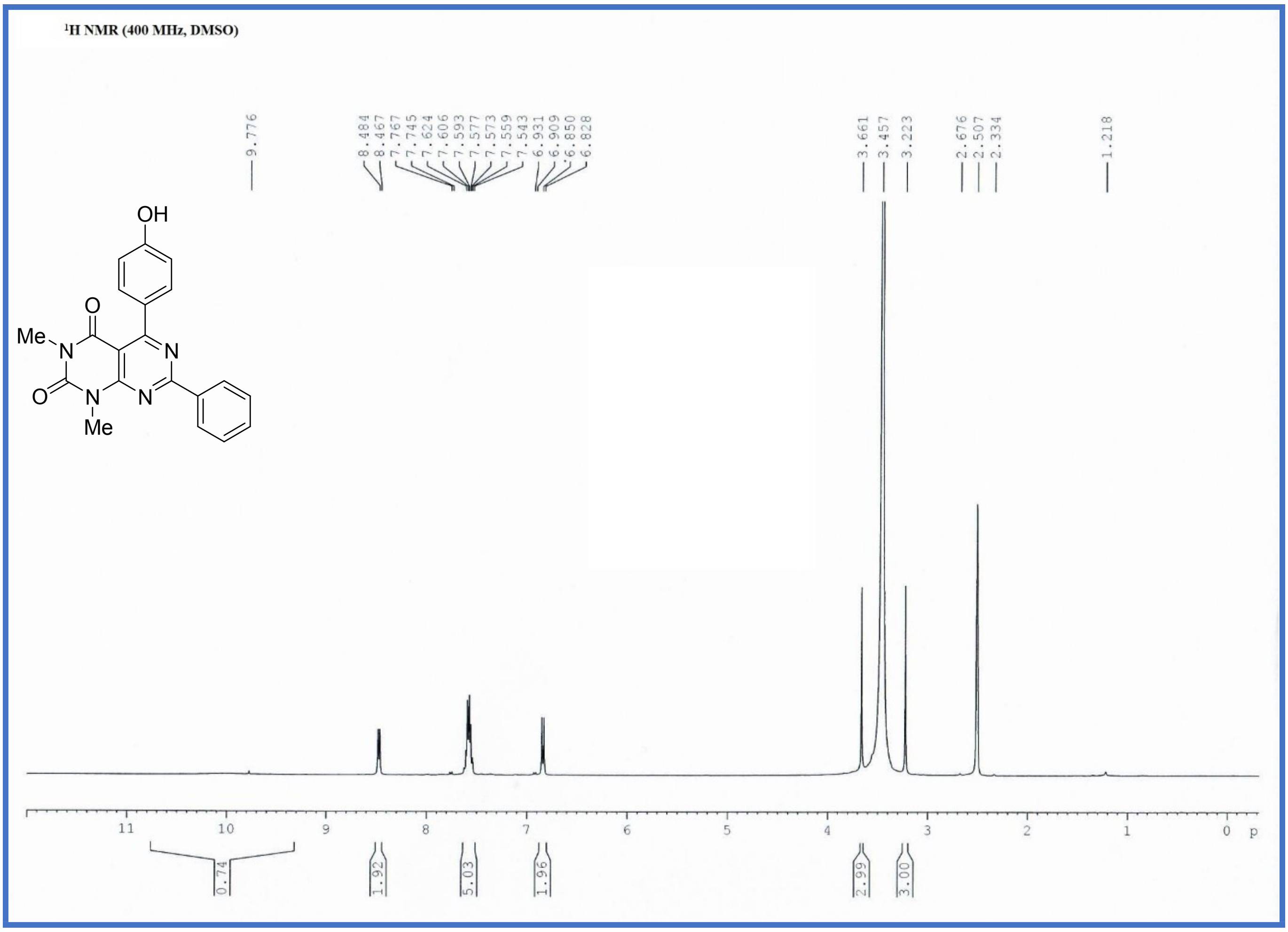

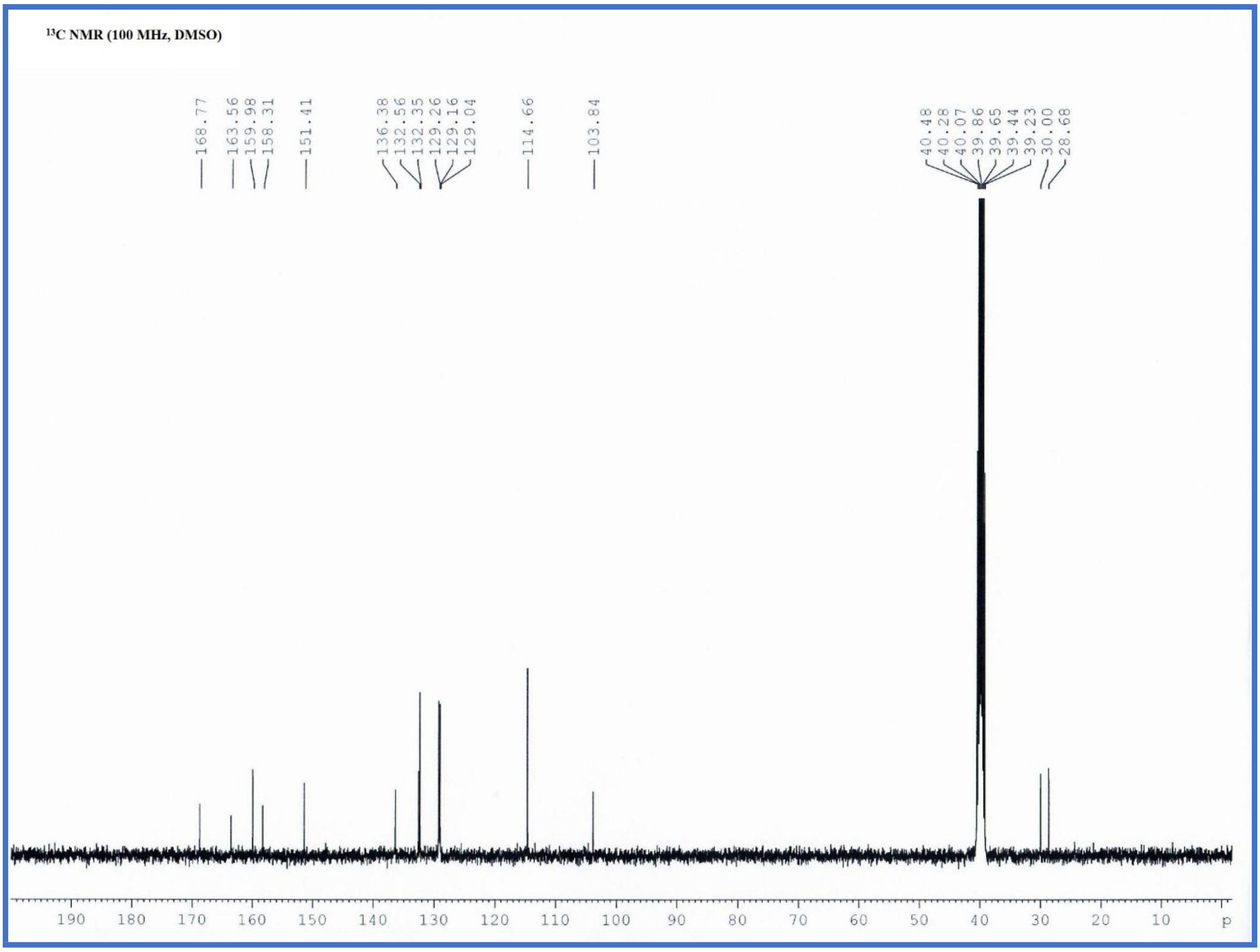

13C NMR (100 MHz, DMSO)
168.77
163.56
159.98
158.31
151.41
136.38
132.56
132.35
129.26
129.16
129.04
114.66
103.84
40.48
40.28
40.07
39.86
39.65
39.44
39.23
30.00
28.68
190 180 170 160 150 140 130 120 110 100 90 80 70 60 50 40 30 20 10 p

5-(4-Bromophenyl)-1,3-dimethyl-7-phenylpyrimido[4,5-*d*]pyrimidine-2,4(1*H*,3*H*)-dione (3g):

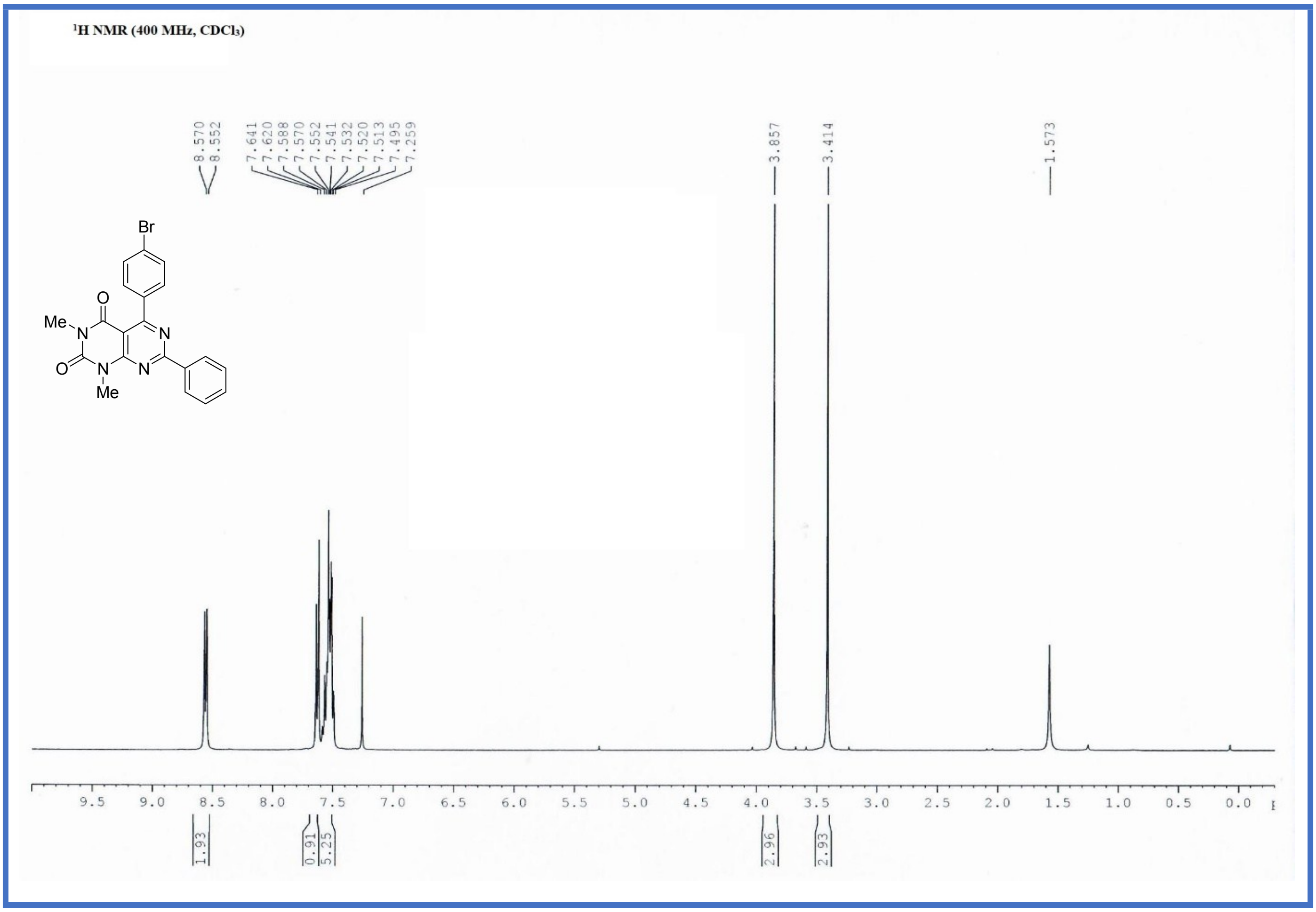

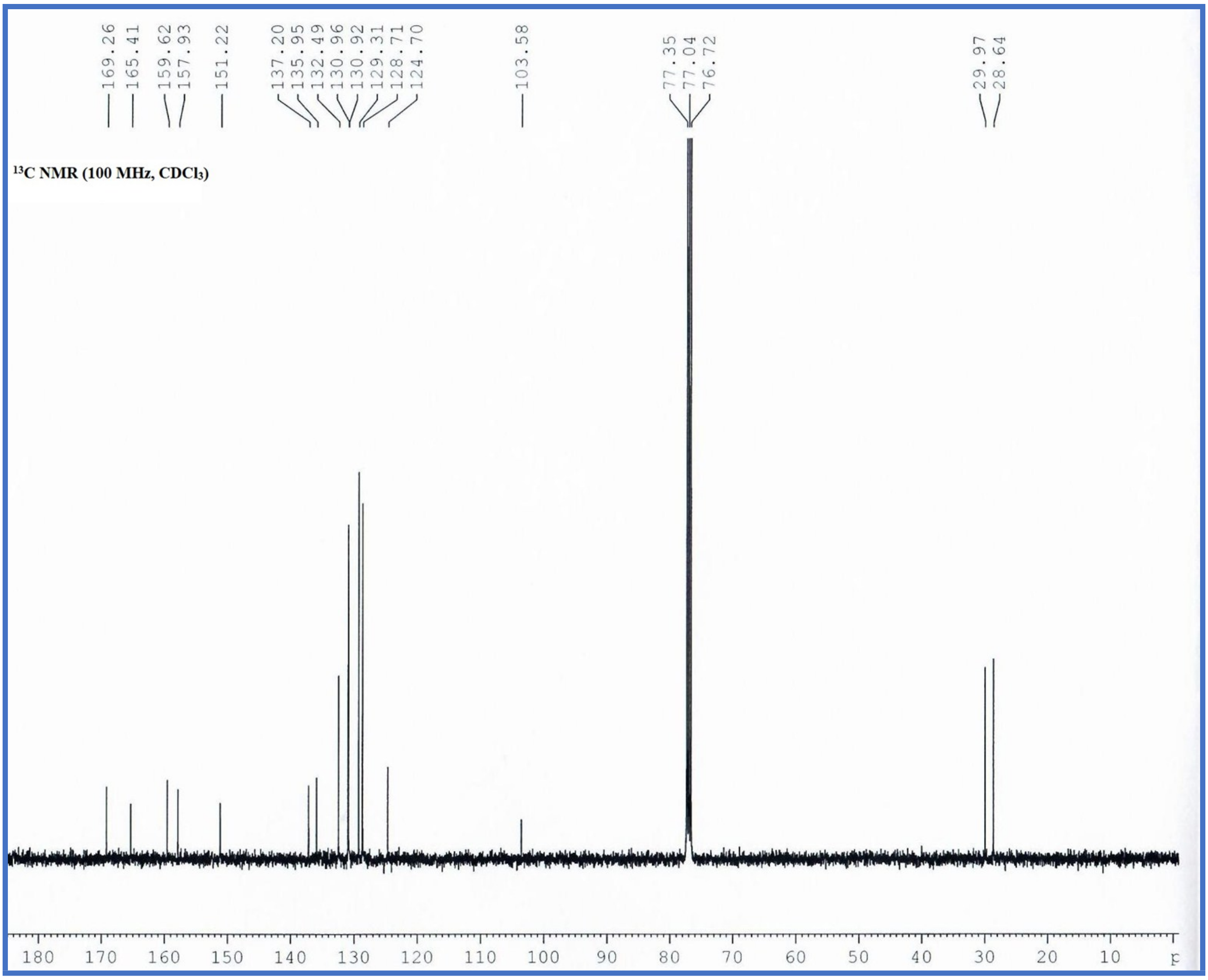

169.26
165.41
159.62
157.93
151.22
137.20
135.95
132.49
130.96
130.92
129.31
128.71
124.70
103.58
77.35
77.04
76.72
29.97
28.64
13C NMR (100 MHz, CDCl3)
180 170 160 150 140 130 120 110 100 90 80 70 60 50 40 30 20 10

5-(4-Chlorophenyl)-1,3-dimethyl-7-phenylpyrimido[4,5-*d*]pyrimidine-2,4(1*H*,3*H*)-dione (3h):

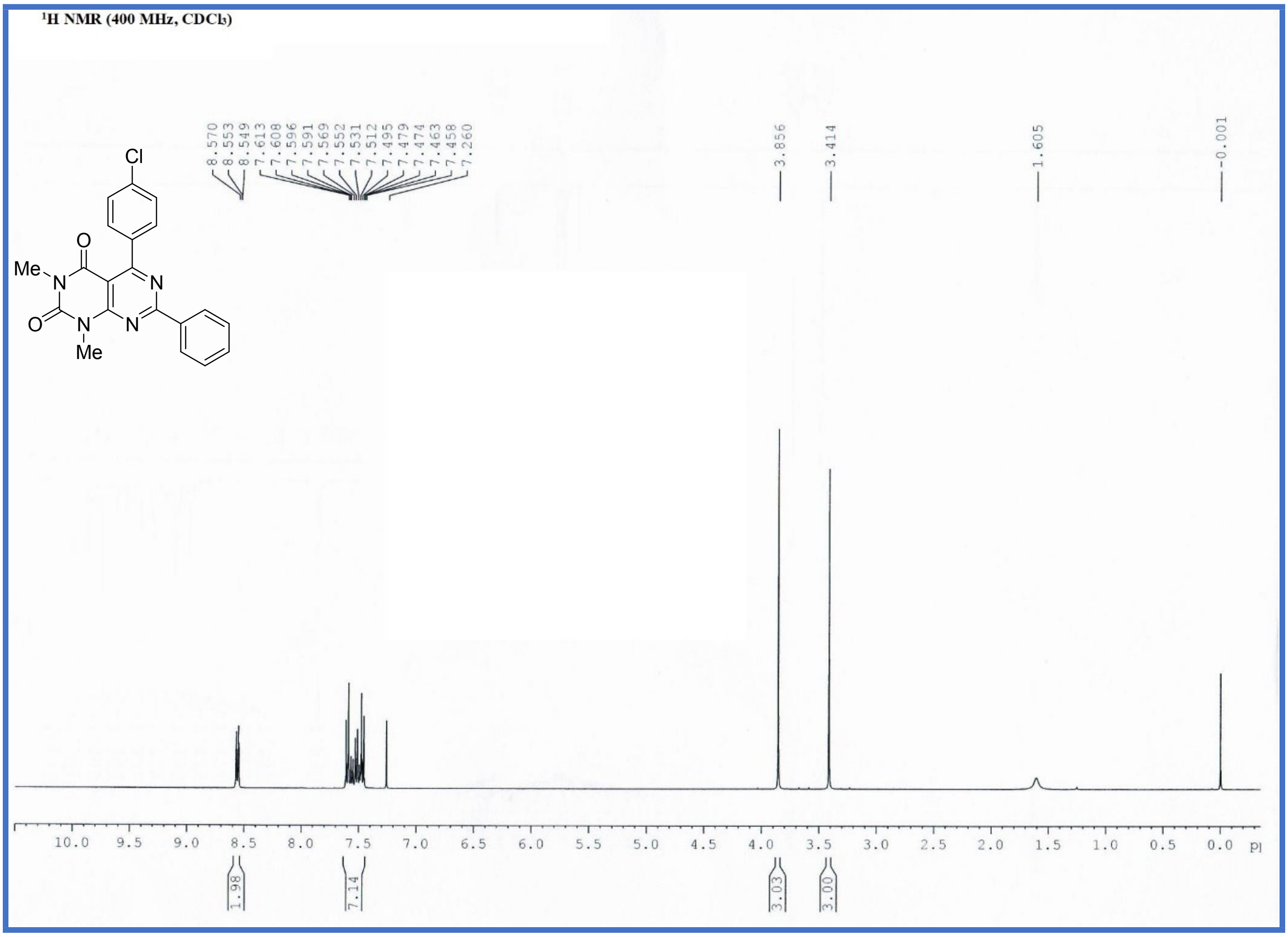

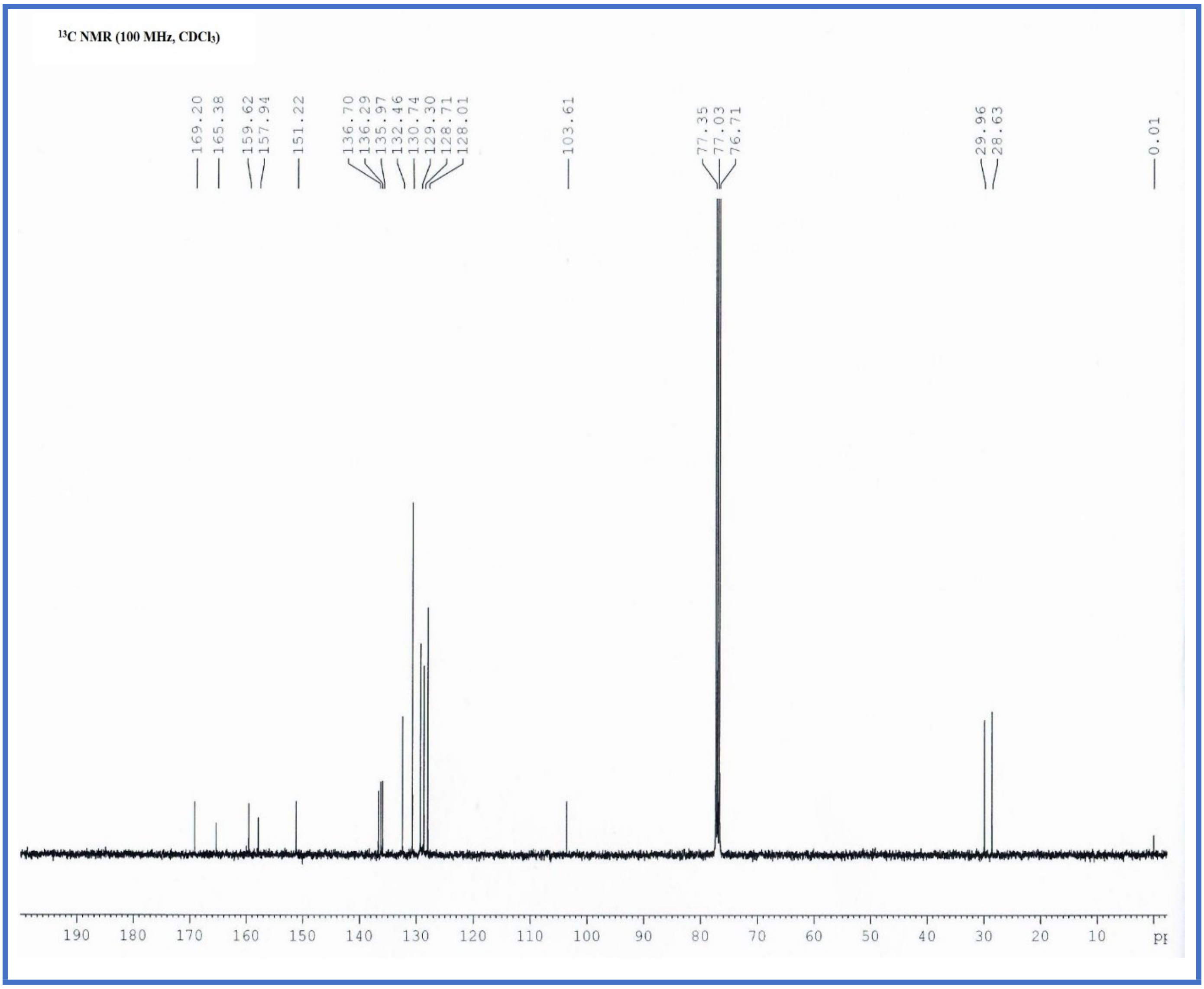

13C NMR (100 MHz, CDCl3)
169.20
165.38
159.62
157.94
151.22
136.70
136.29
135.97
132.46
130.74
129.30
128.71
128.01
103.61
77.35
77.03
76.71
29.96
28.63
0.01
190 180 170 160 150 140 130 120 110 100 90 80 70 60 50 40 30 20 10 pp

1,3-Dimethyl-5-(2-chlorophenyl)-7-phenylpyrimido[4,5-*d*]pyrimidine-2,4(1*H*,3*H*)-dione (3i):

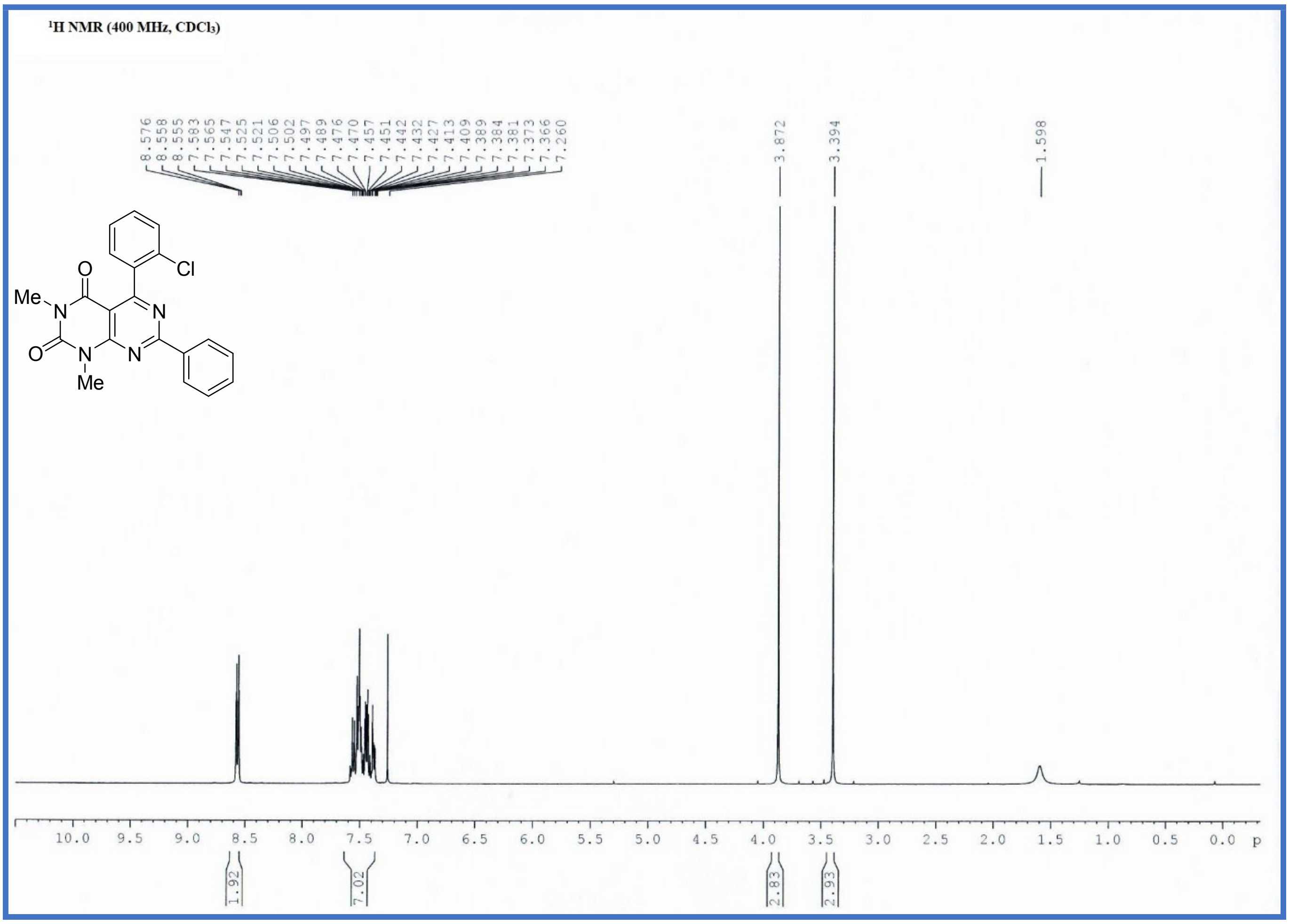

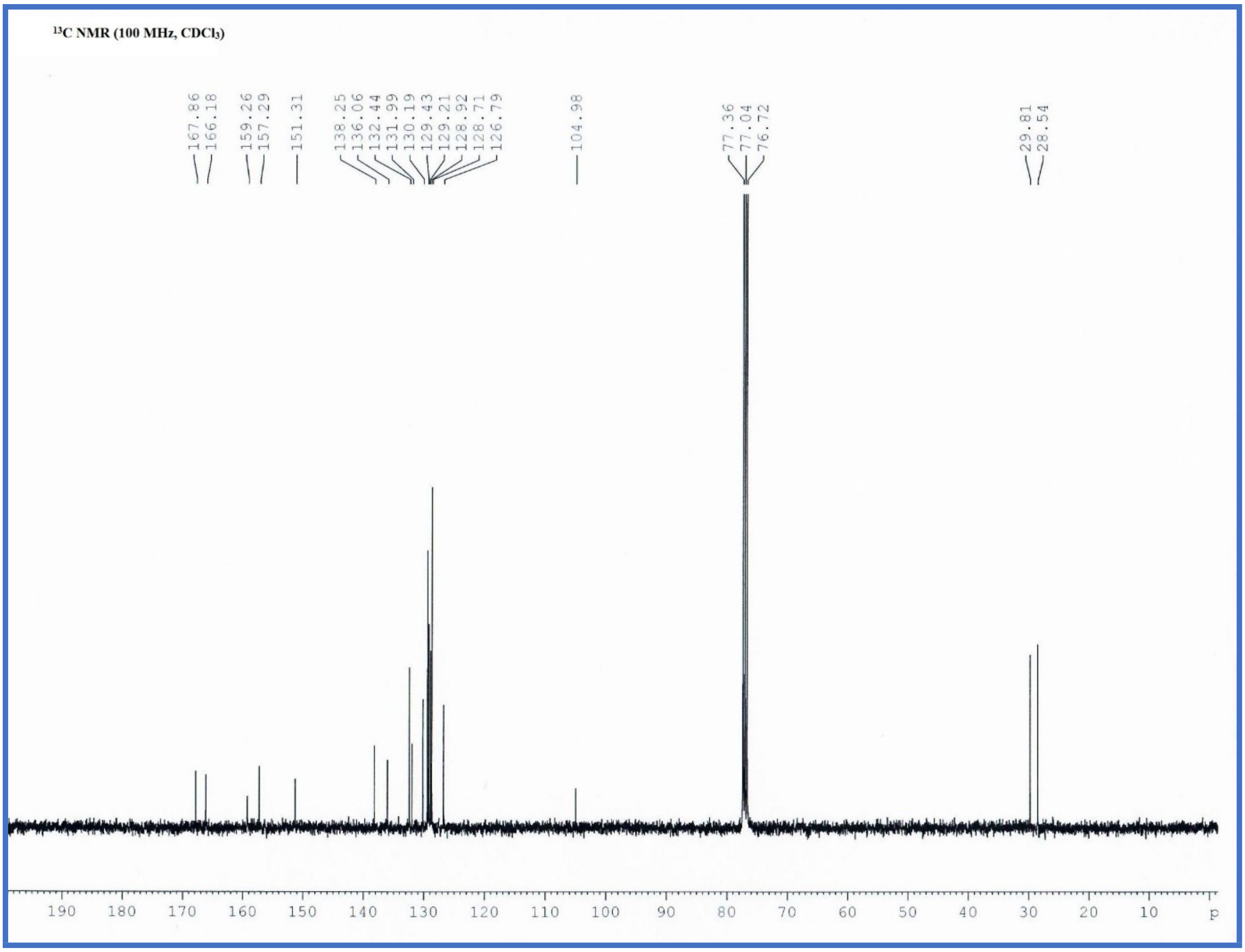
13C NMR (100 MHz, CDCl3)
167.86
166.18
159.26
157.29
151.31
138.25
136.06
132.44
131.99
130.19
129.43
129.21
128.92
128.71
126.79
104.98
77.36
77.04
76.72
29.81
28.54
190
180
170
160
150
140
130
120
110
100
90
80
70
60
50
40
30
20
10
p

5-(4-Fluorophenyl)-1,3-dimethyl-7-phenylpyrimido[4,5-*d*]pyrimidine-2,4(1*H*,3*H*)-dione (3j):

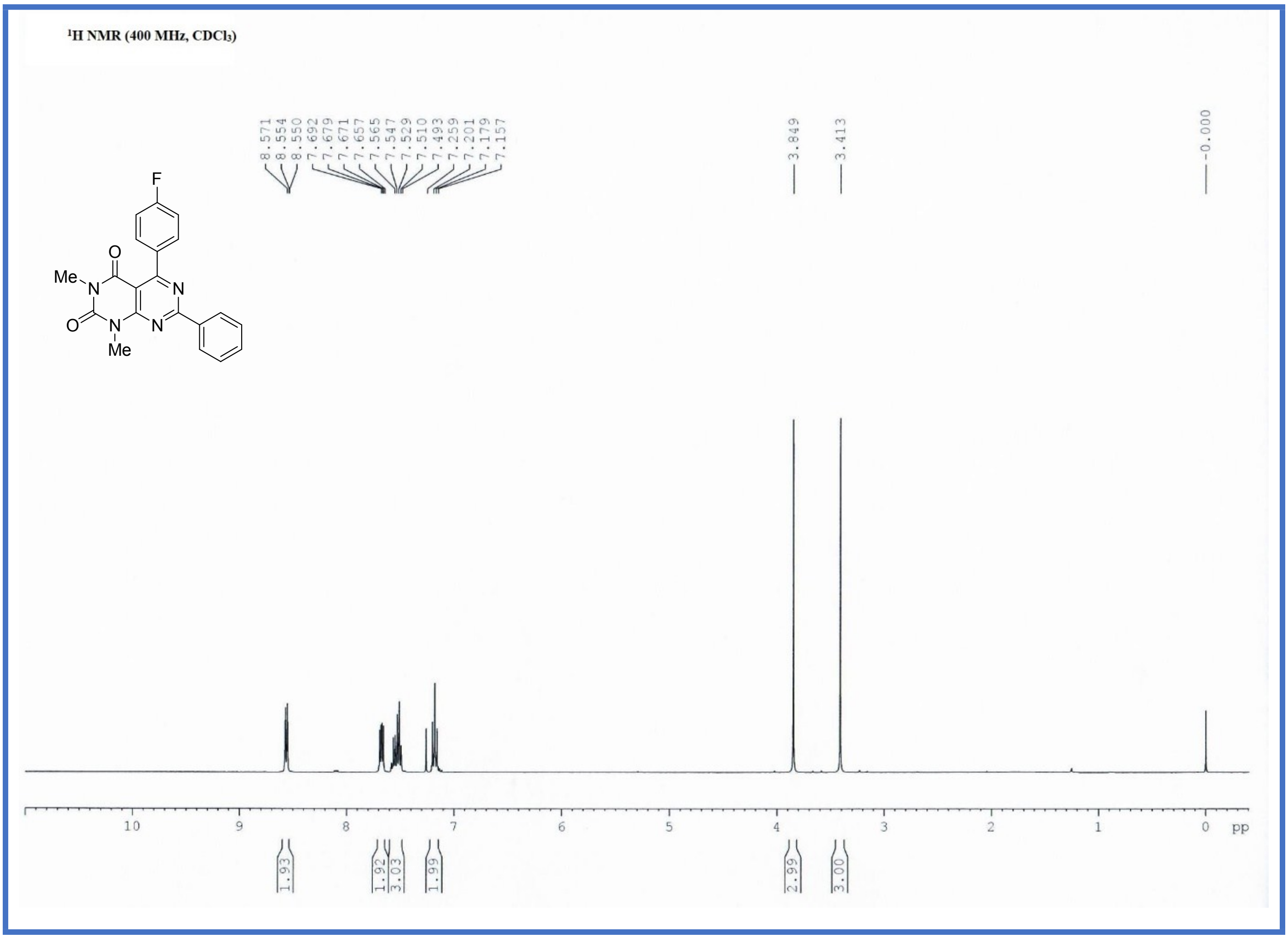

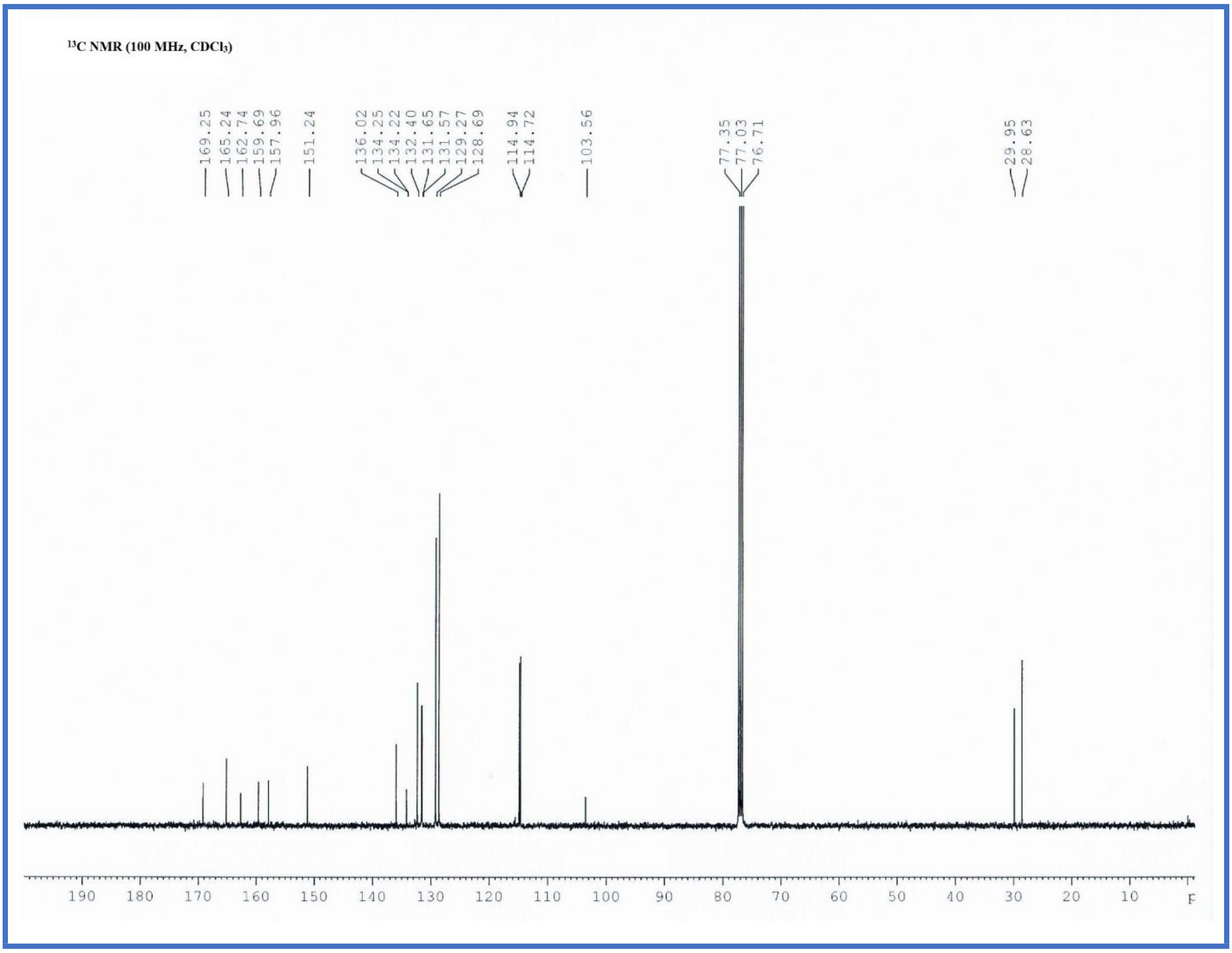

¹³C NMR (100 MHz, CDCl₃)
169.25
165.24
162.74
159.69
157.96
151.24
136.02
134.25
134.22
132.40
131.65
131.57
129.27
128.69
114.94
114.72
103.56
77.35
77.03
76.71
29.95
28.63
190 180 170 160 150 140 130 120 110 100 90 80 70 60 50 40 30 20 10

1,3-Dimethyl-5-(4-cyanophenyl)-7-phenylpyrimido[4,5-*d*]pyrimidine-2,4(1*H*,3*H*)-dione (3k):

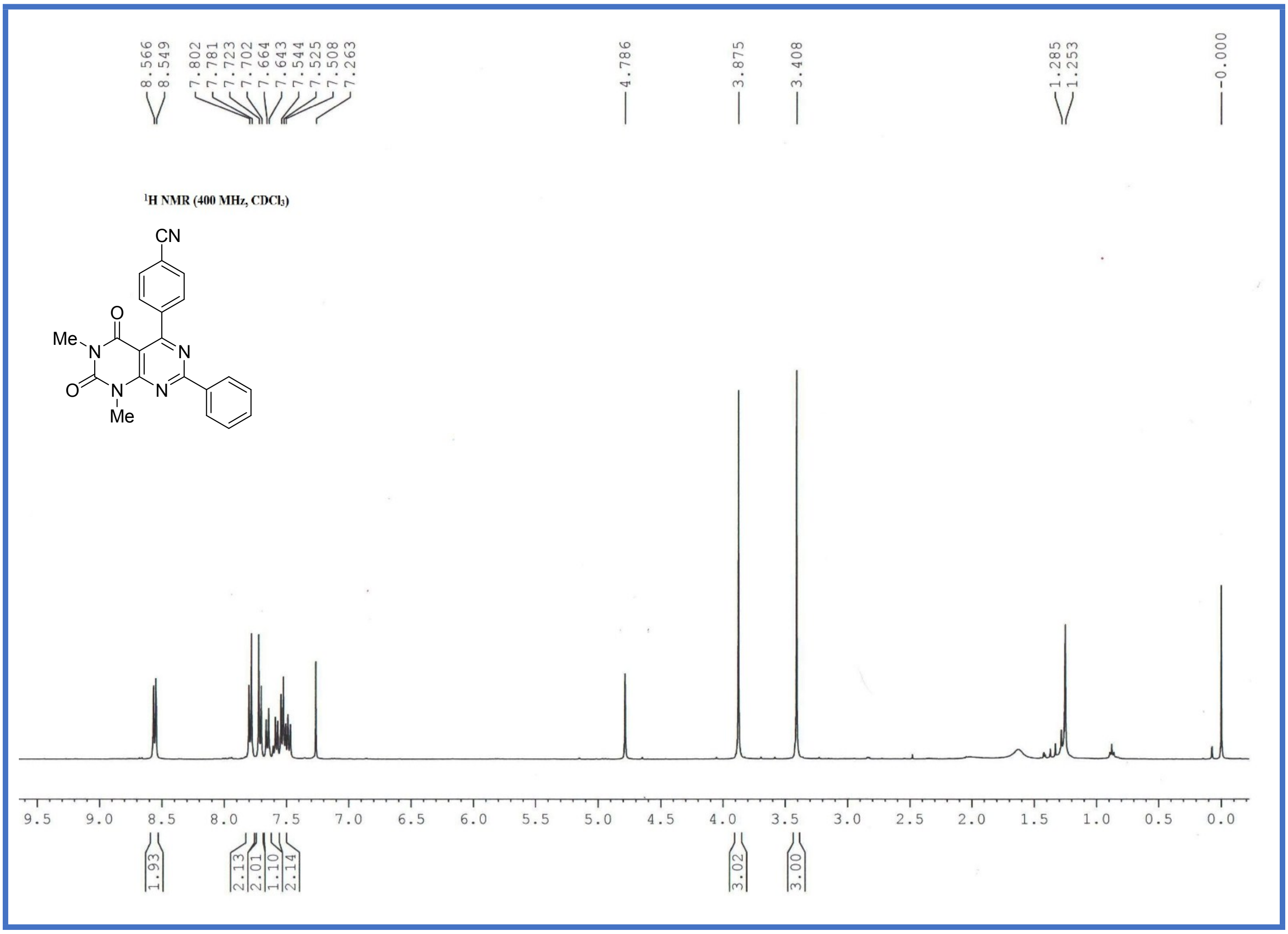

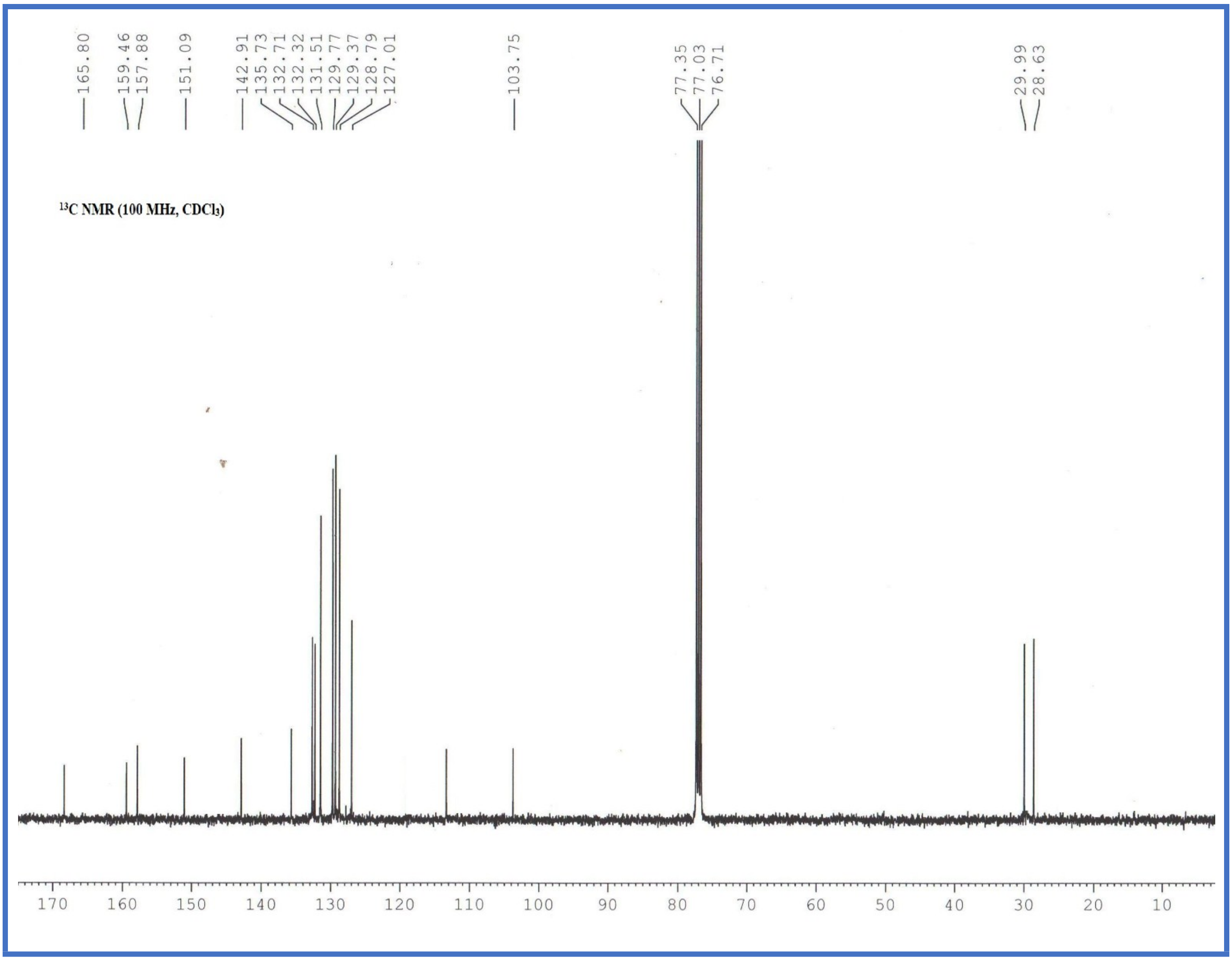

1,3-Dimethyl-5-(naphthalen-1-yl)-7-phenylpyrimido[4,5-*d*]pyrimidine-2,4(1*H*,3*H*)-dione (3l):

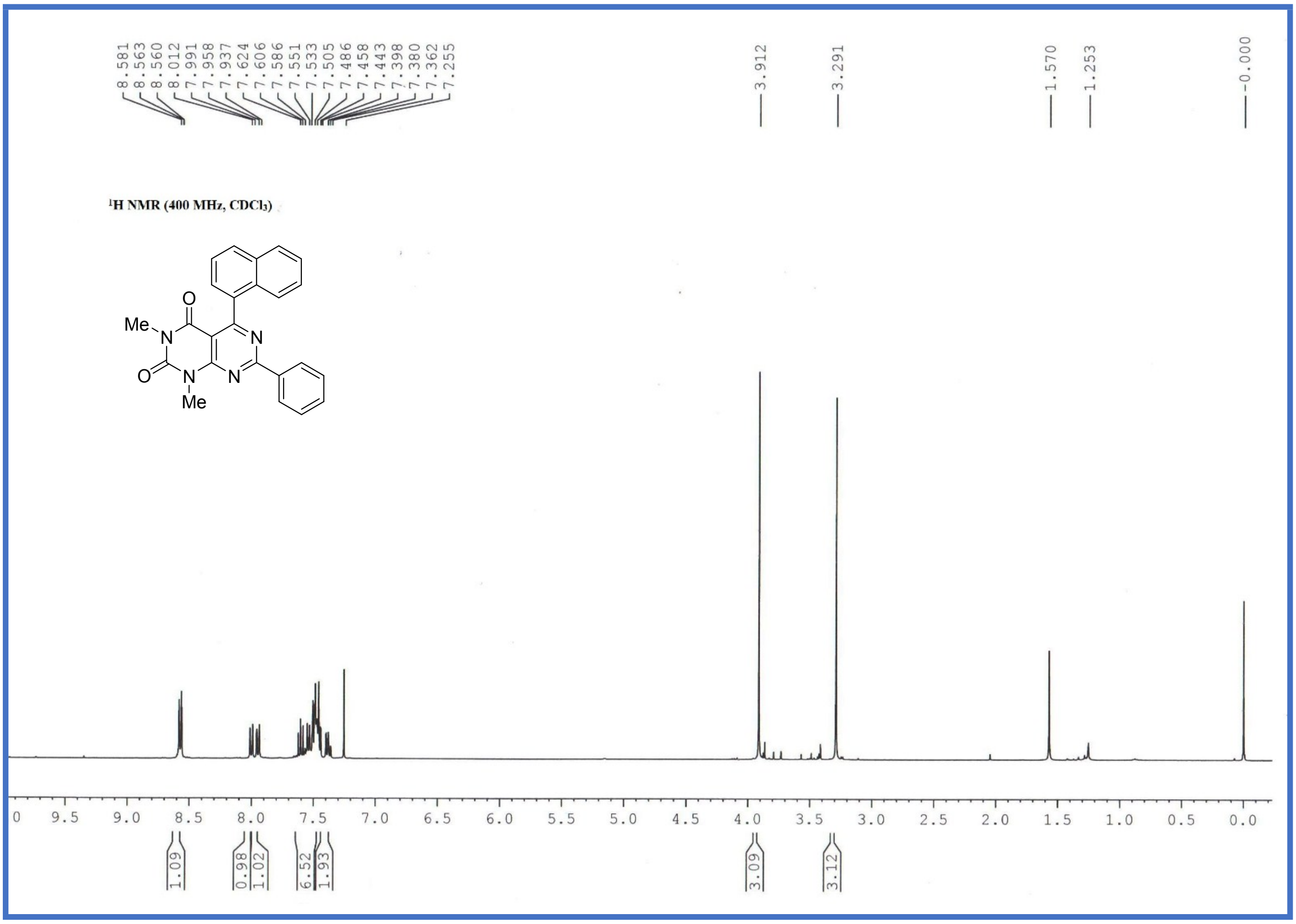

8.581
8.563
8.560
8.012
7.991
7.958
7.937
7.624
7.606
7.586
7.551
7.533
7.505
7.486
7.458
7.443
7.398
7.380
7.362
7.255
3.912
3.291
1.570
1.253
-0.000
1H NMR (400 MHz, CDCl3)
Me
N
O
Me
1.09
0.98
1.02
6.52
1.93
3.09
3.12

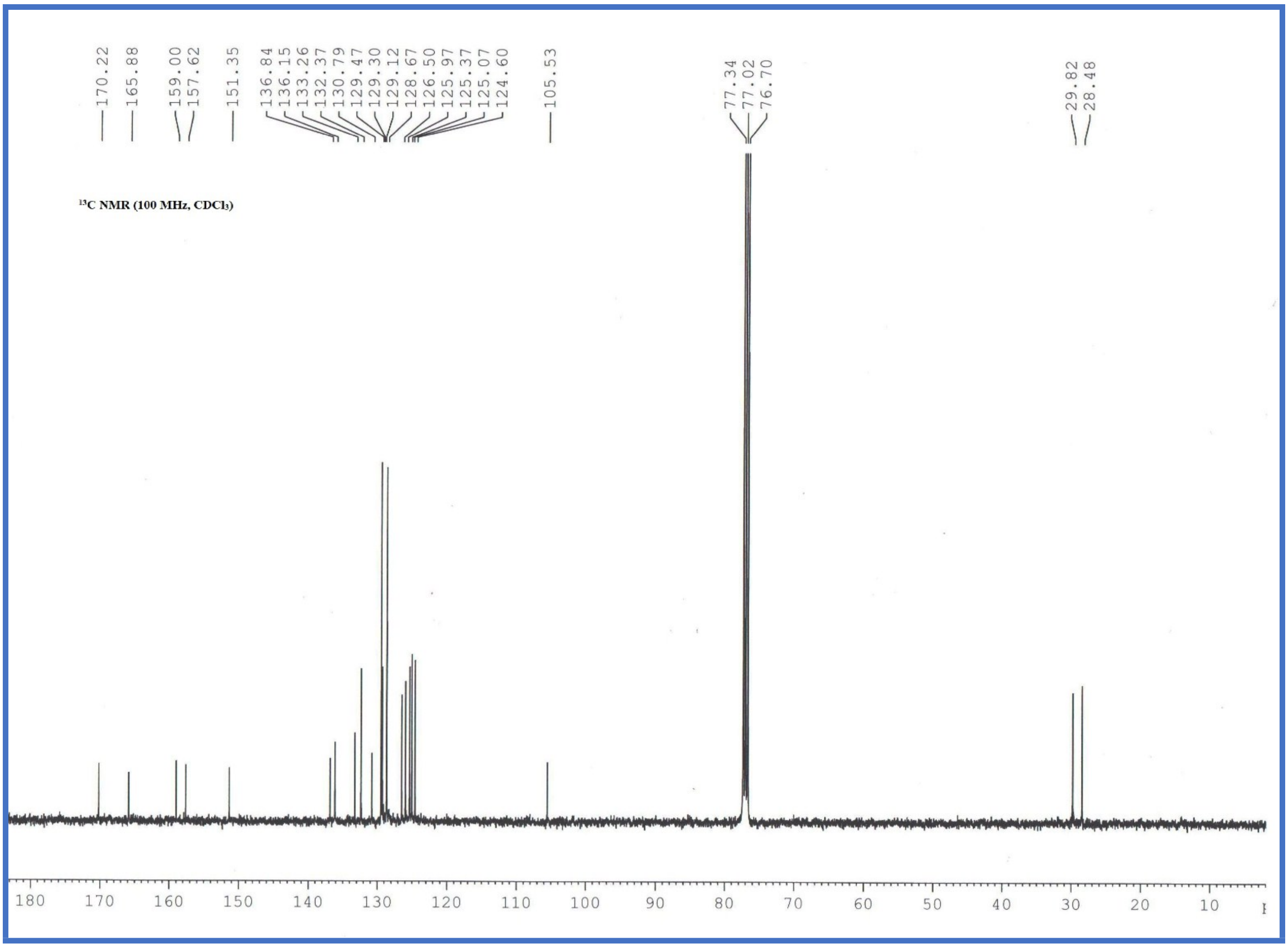

13C NMR (100 MHz, CDCl3)
170.22
165.88
159.00
157.62
151.35
136.84
136.15
133.26
132.37
130.79
129.47
129.30
129.12
128.67
126.50
125.97
125.37
125.07
124.60
105.53
77.34
77.02
76.70
29.82
28.48
180
170
160
150
140
130
120
110
100
90
80
70
60
50
40
30
20
10

1,3-Dimethyl-7-phenyl-5-(3,4,5-trimethoxyphenyl)pyrimido[4,5-*d*]pyrimidine-2,4(1*H*,3*H*)-dione (3m):

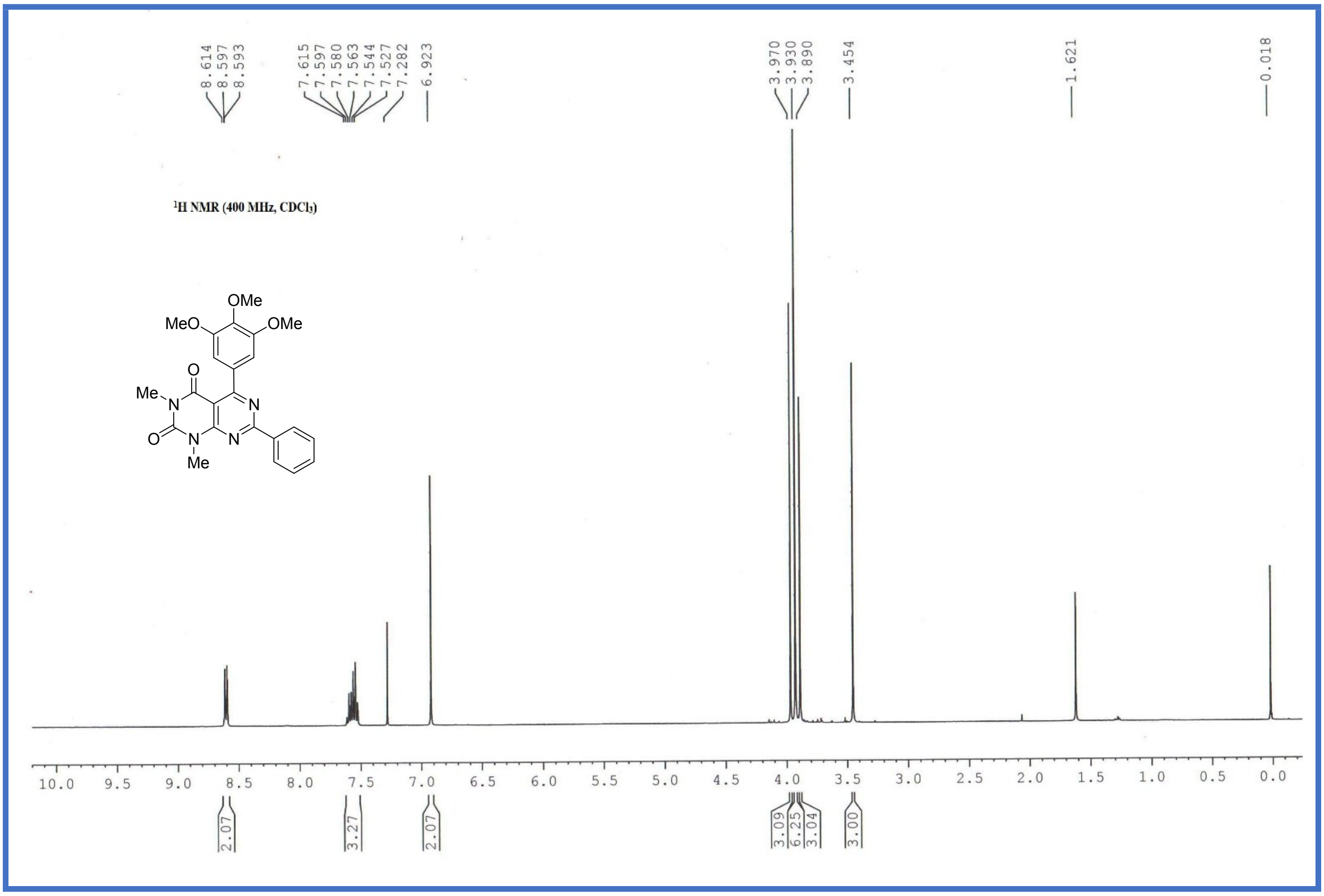

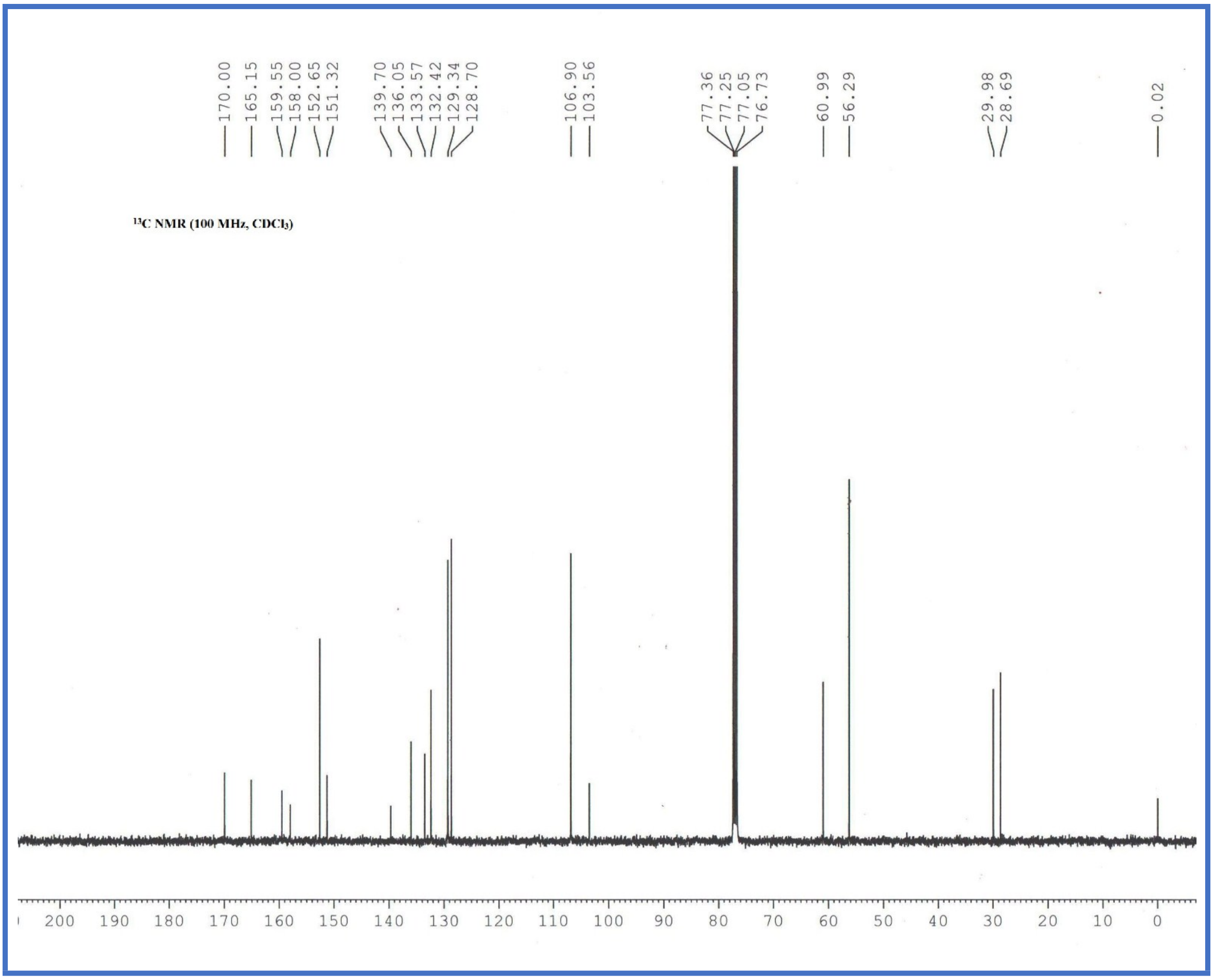

1,3-Dimethyl-7-phenyl-5-(thiophen-2-yl)pyrimido[4,5-*d*]pyrimidine-2,4(1*H*,3*H*)-dione (3n):

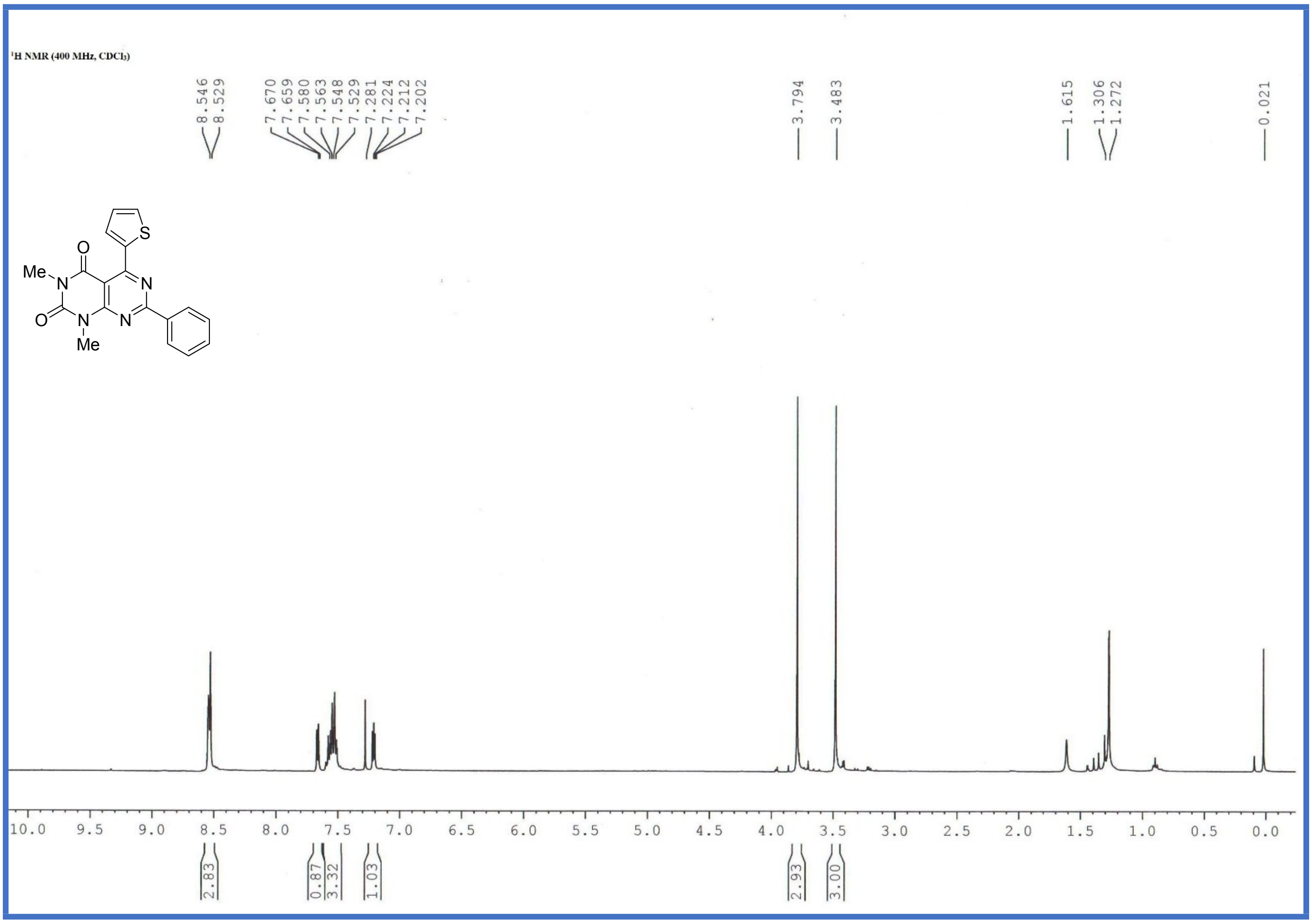

¹H NMR (400 MHz, CDCl₃)
8.546
8.529
7.670
7.659
7.580
7.563
7.548
7.529
7.281
7.224
7.212
7.202
3.794
3.483
1.615
1.306
1.272
0.021
Me
N
O
O
N
Me
N
N
S
10.0 9.5 9.0 8.5 8.0 7.5 7.0 6.5 6.0 5.5 5.0 4.5 4.0 3.5 3.0 2.5 2.0 1.5 1.0 0.5 0.0
2.83
0.87
3.32
1.03
2.93
3.00

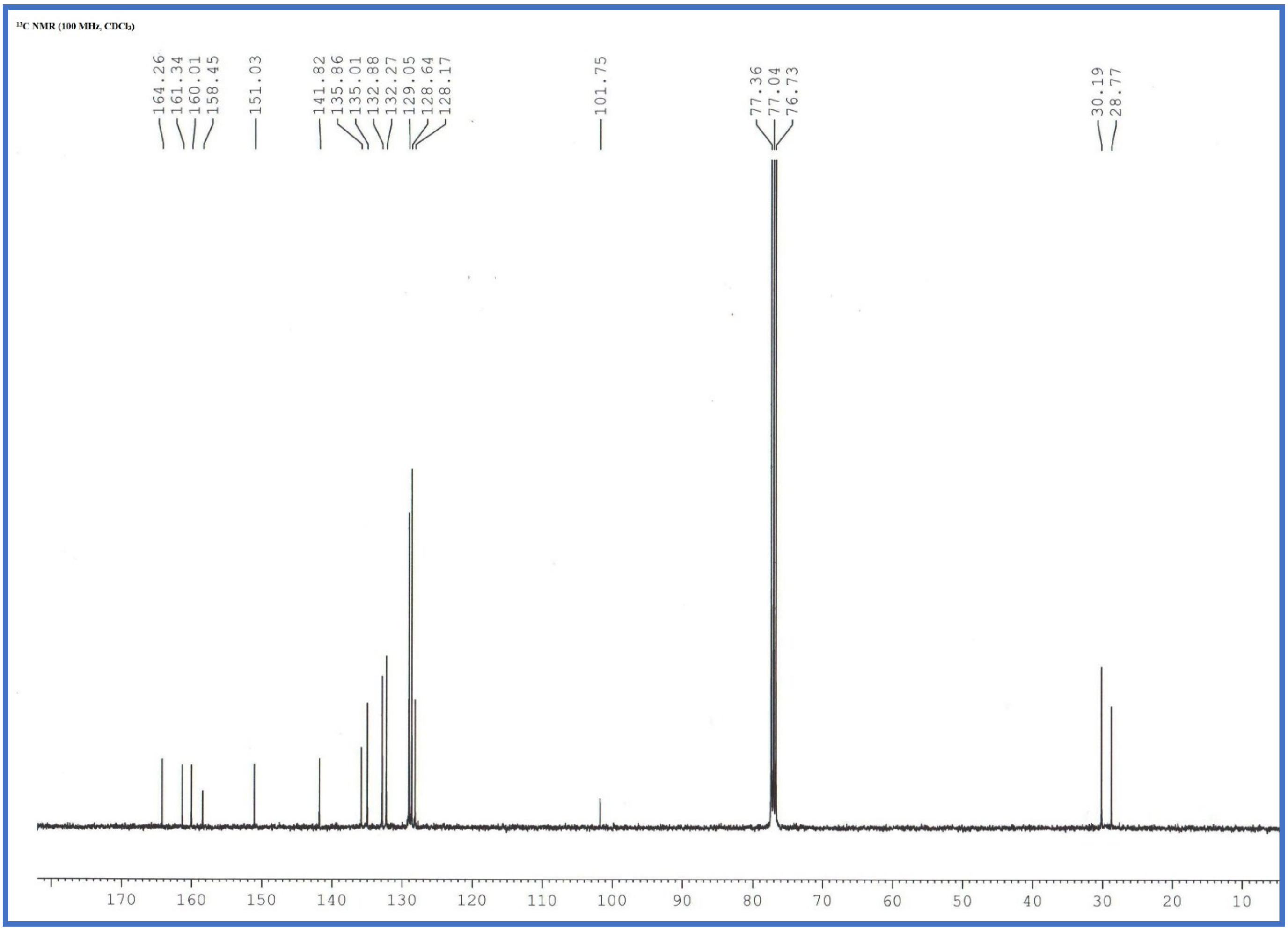

^{13}C NMR (100 MHz, CDCl$_3$)
164.26
161.34
160.01
158.45
151.03
141.82
135.86
135.01
132.88
132.27
129.05
128.64
128.17
101.75
77.36
77.04
76.73
30.19
28.77
170 160 150 140 130 120 110 100 90 80 70 60 50 40 30 20 10

1,3-Dimethyl-7-phenyl-5-(pyridin-2-yl)pyrimido[4,5-*d*]pyrimidine-2,4(1*H*,3*H*)-dione (3o):

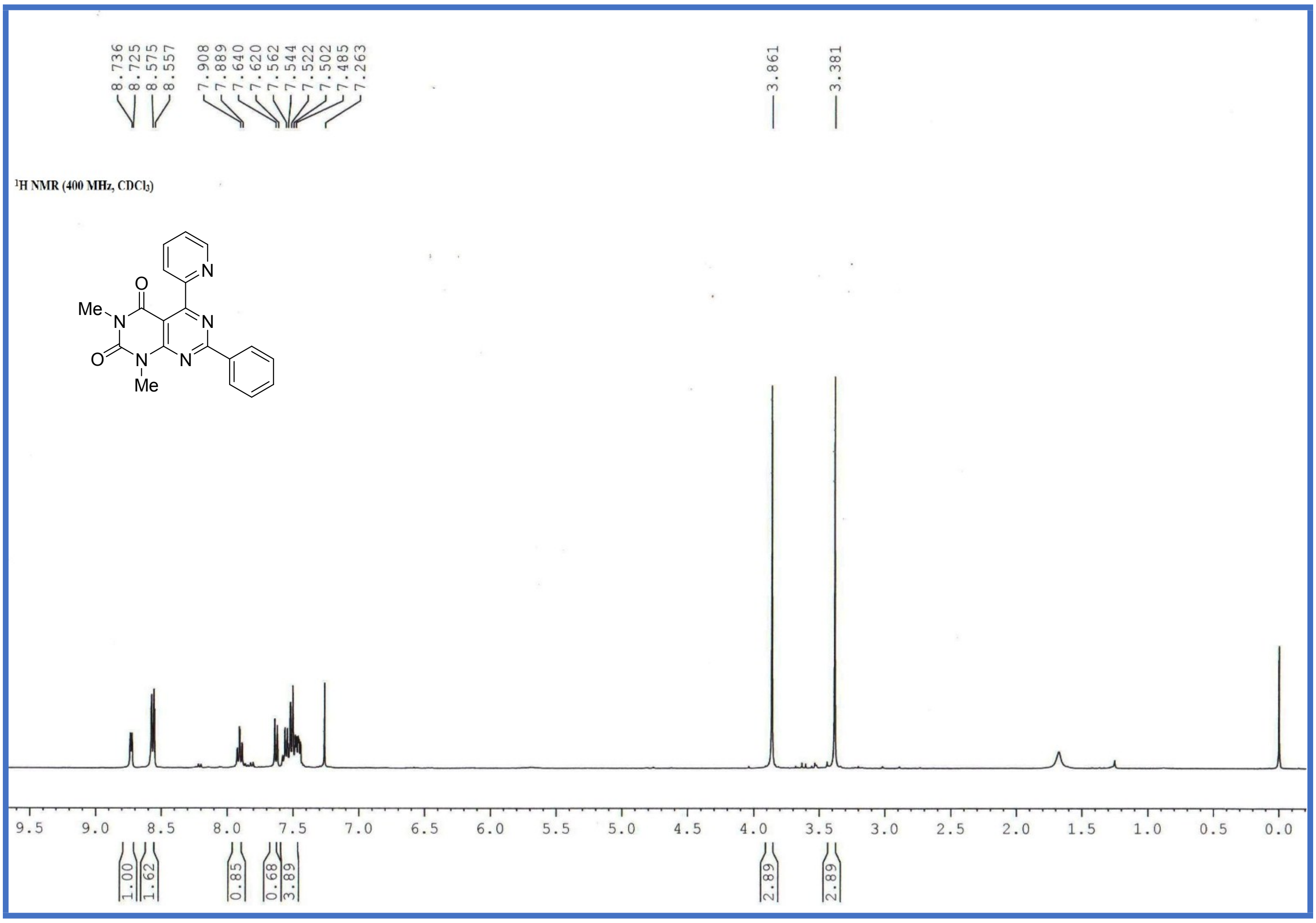

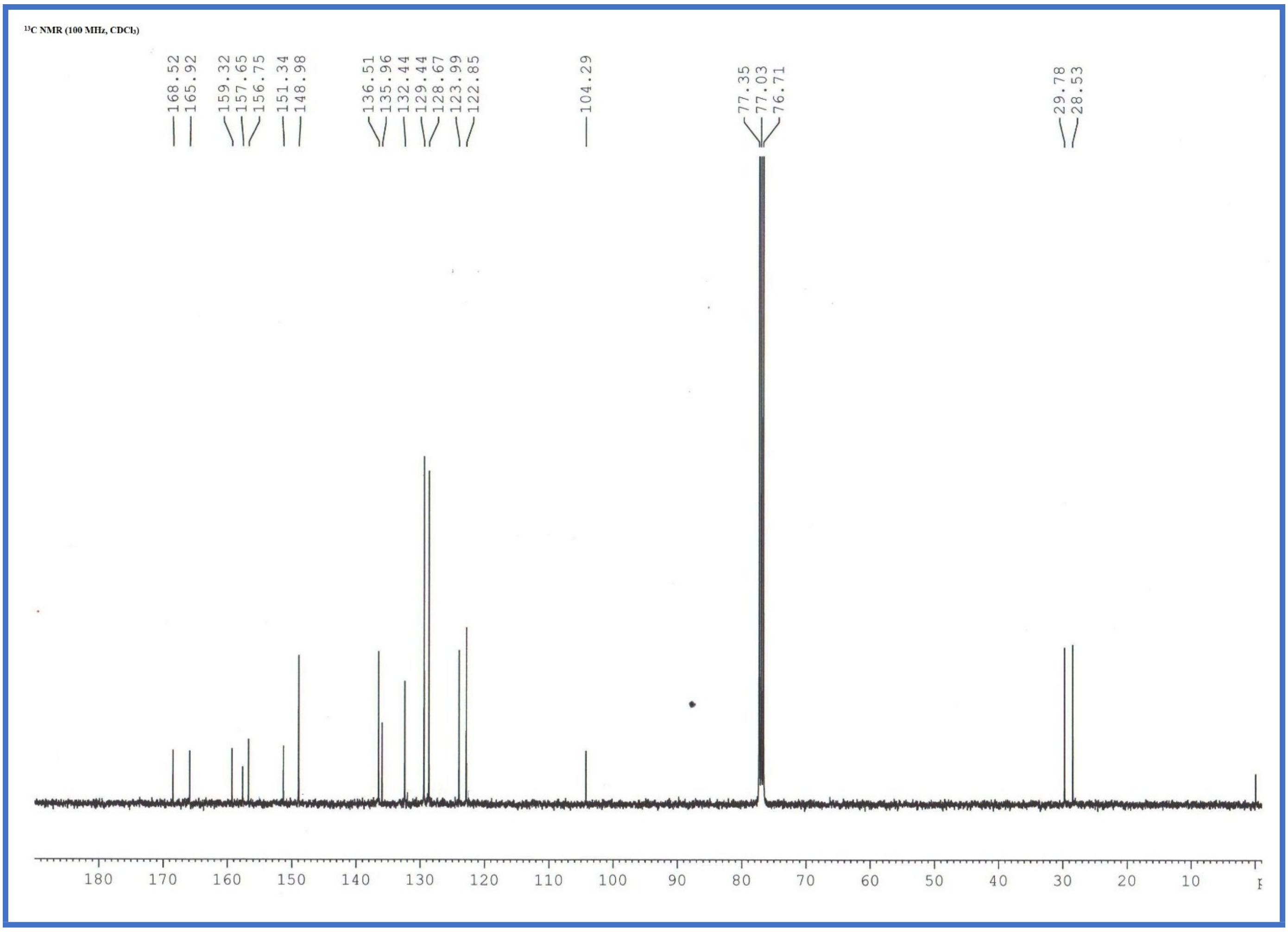

1,3-Dimethyl-5-phenyl-7-(*p*-tolyl)pyrimido[4,5-*d*]pyrimidine-2,4(1*H*,3*H*)-dione (3p):

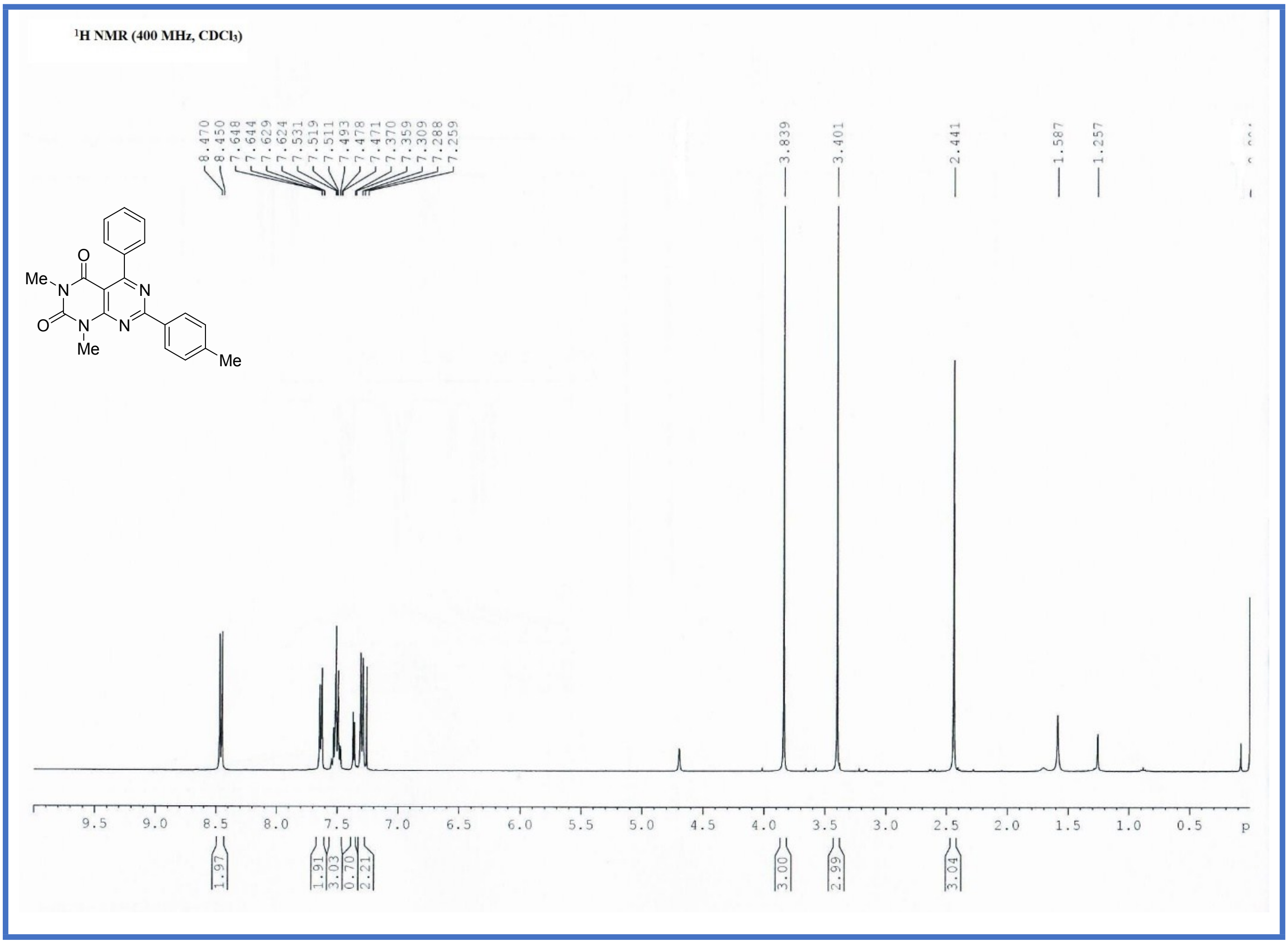

¹H NMR (400 MHz, CDCl₃)
Me
N
O
O
N
Me
N
N
Me

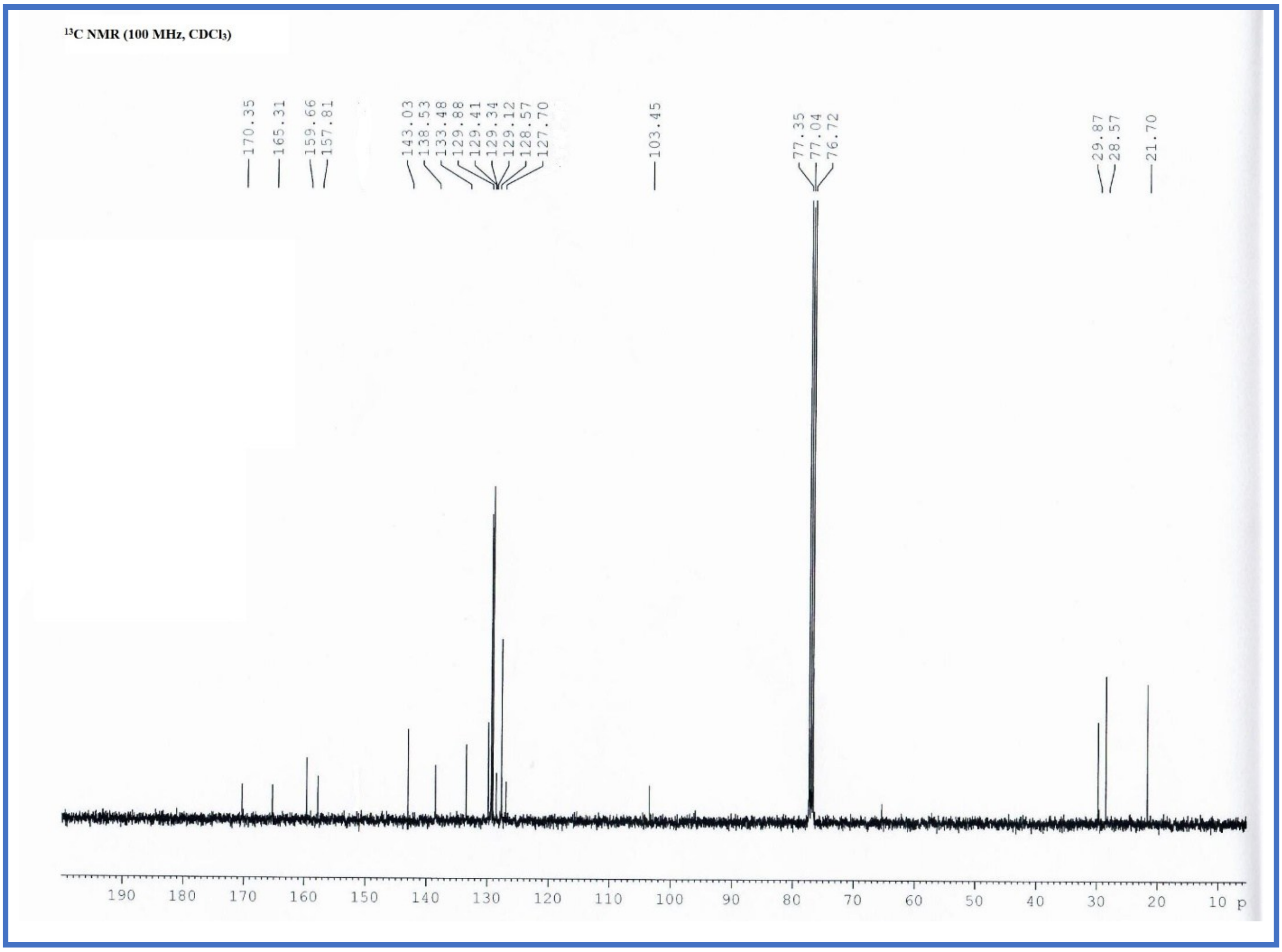

7-(4-Fluorophenyl)-1,3-dimethyl-5-phenylpyrimido[4,5-*d*]pyrimidine-2,4(1*H*,3*H*)-dione (3q):

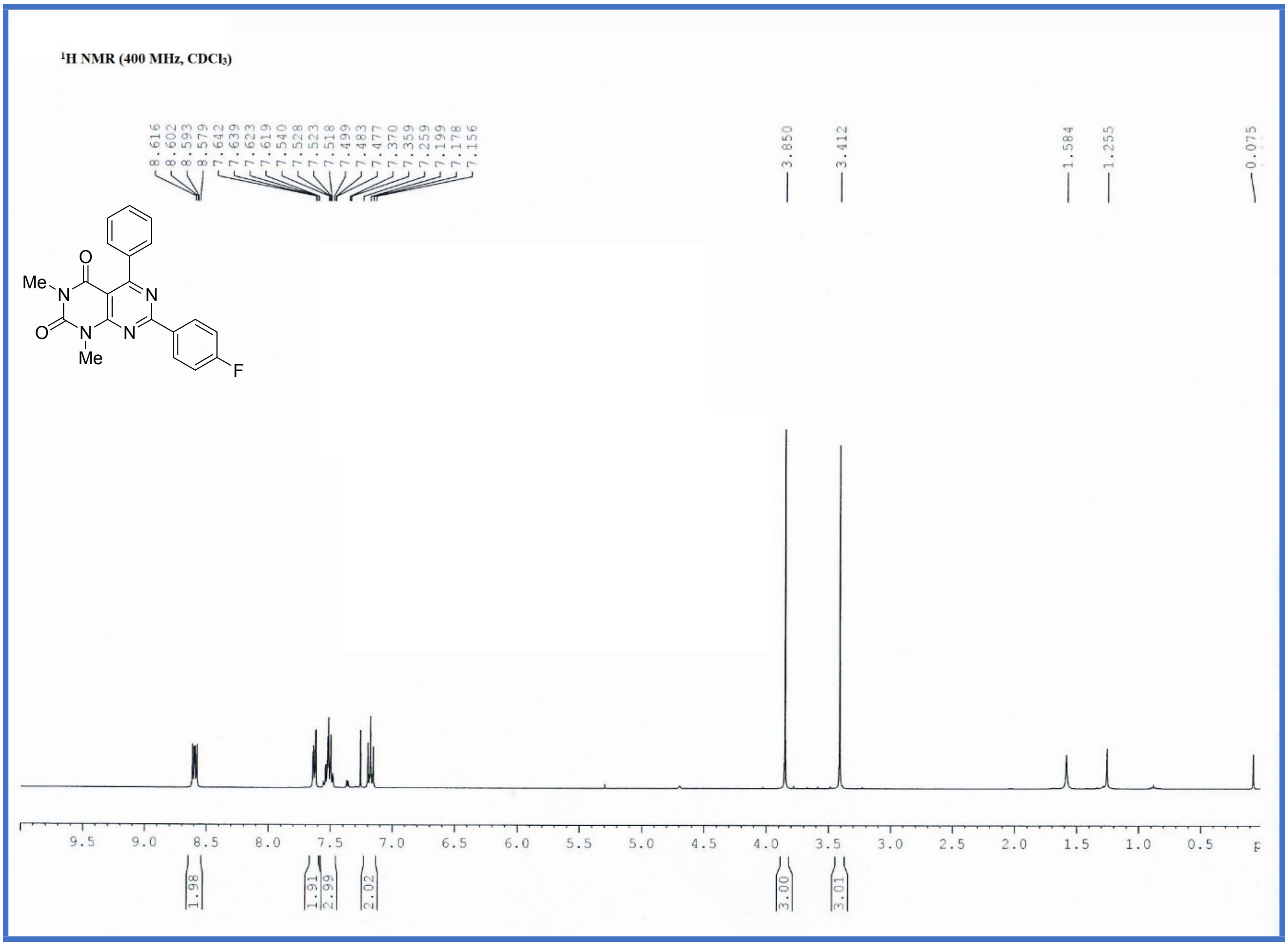
¹H NMR (400 MHz, CDCl₃)
8.616
8.602
8.593
8.579
7.642
7.639
7.623
7.619
7.540
7.528
7.523
7.518
7.499
7.483
7.477
7.370
7.359
7.259
7.199
7.178
7.156
3.850
3.412
1.584
1.255
0.075
Me
N
O
N
Me
N
N
F
9.5
9.0
8.5
8.0
7.5
7.0
6.5
6.0
5.5
5.0
4.5
4.0
3.5
3.0
2.5
2.0
1.5
1.0
0.5
1.98
1.91
2.99
2.02
3.00
3.01

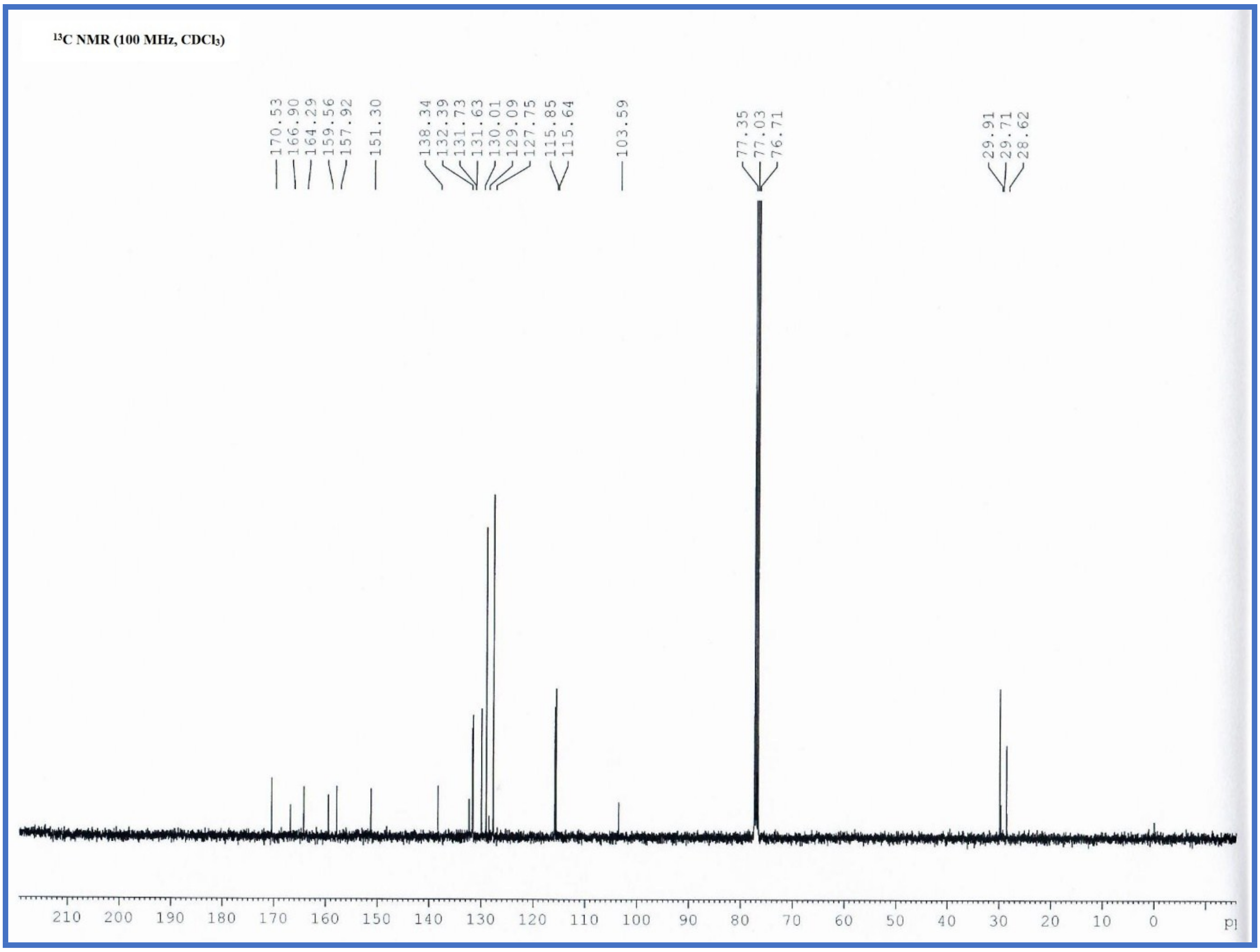
13C NMR (100 MHz, CDCl3)
170.53
166.90
164.29
159.56
157.92
151.30
138.34
132.39
131.73
131.63
130.01
129.09
127.75
115.85
115.64
103.59
77.35
77.03
76.71
29.91
29.71
28.62
210 200 190 180 170 160 150 140 130 120 110 100 90 80 70 60 50 40 30 20 10 0

7-(4-Chlorophenyl)-1,3-dimethyl-5-phenylpyrimido[4,5-*d*]pyrimidine-2,4(1*H*,3*H*)-dione (3r):

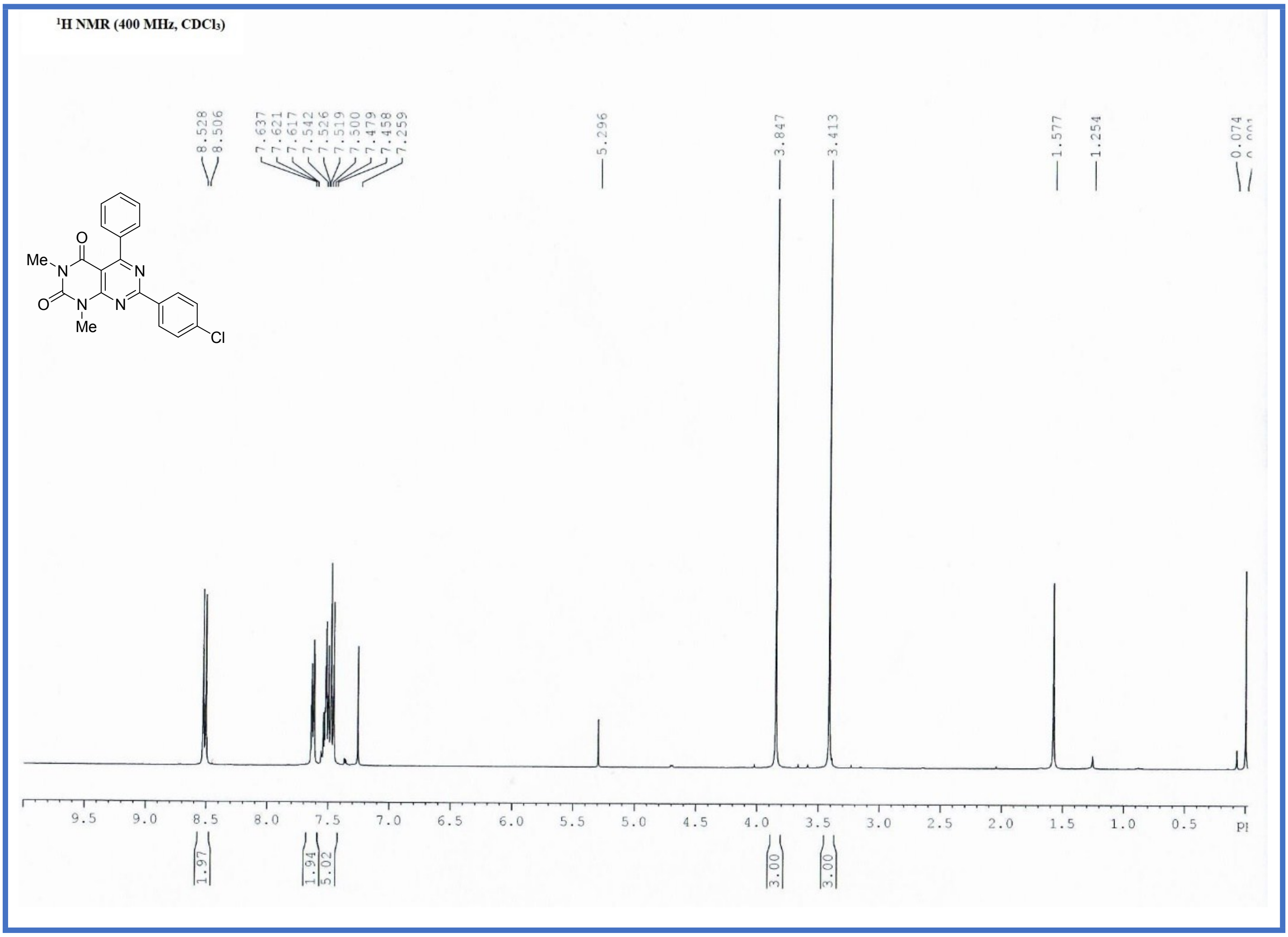

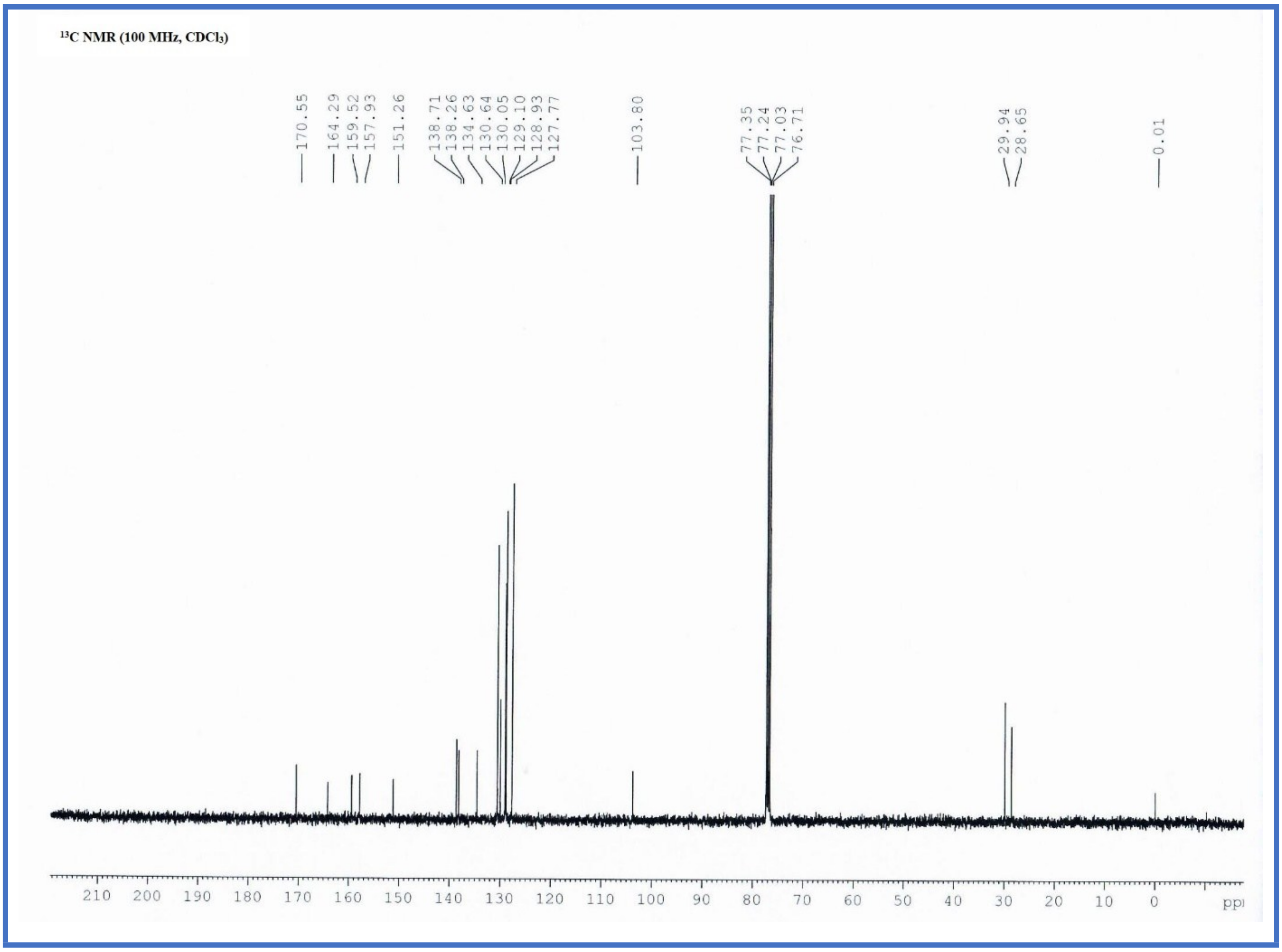

13C NMR (100 MHz, CDCl3)
170.55
164.29
159.52
157.93
151.26
138.71
138.26
134.63
130.64
130.05
129.10
128.93
127.77
103.80
77.35
77.24
77.03
76.71
29.94
28.65
0.01
210 200 190 180 170 160 150 140 130 120 110 100 90 80 70 60 50 40 30 20 10 0 ppm

7-(4-Bromophenyl)-1,3-dimethyl-5-phenylpyrimido[4,5-*d*]pyrimidine-2,4(1*H*,3*H*)-dione (3s):

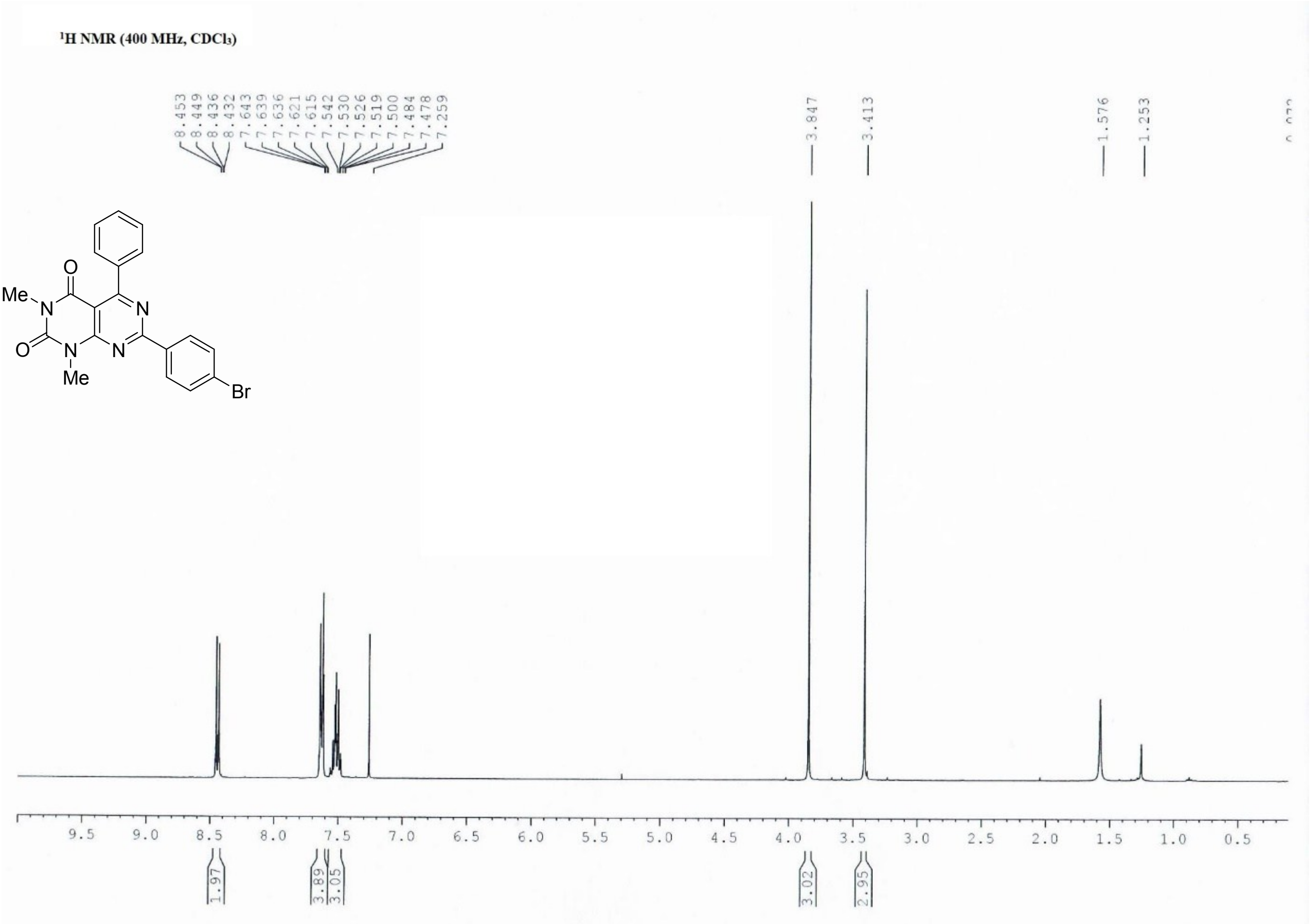

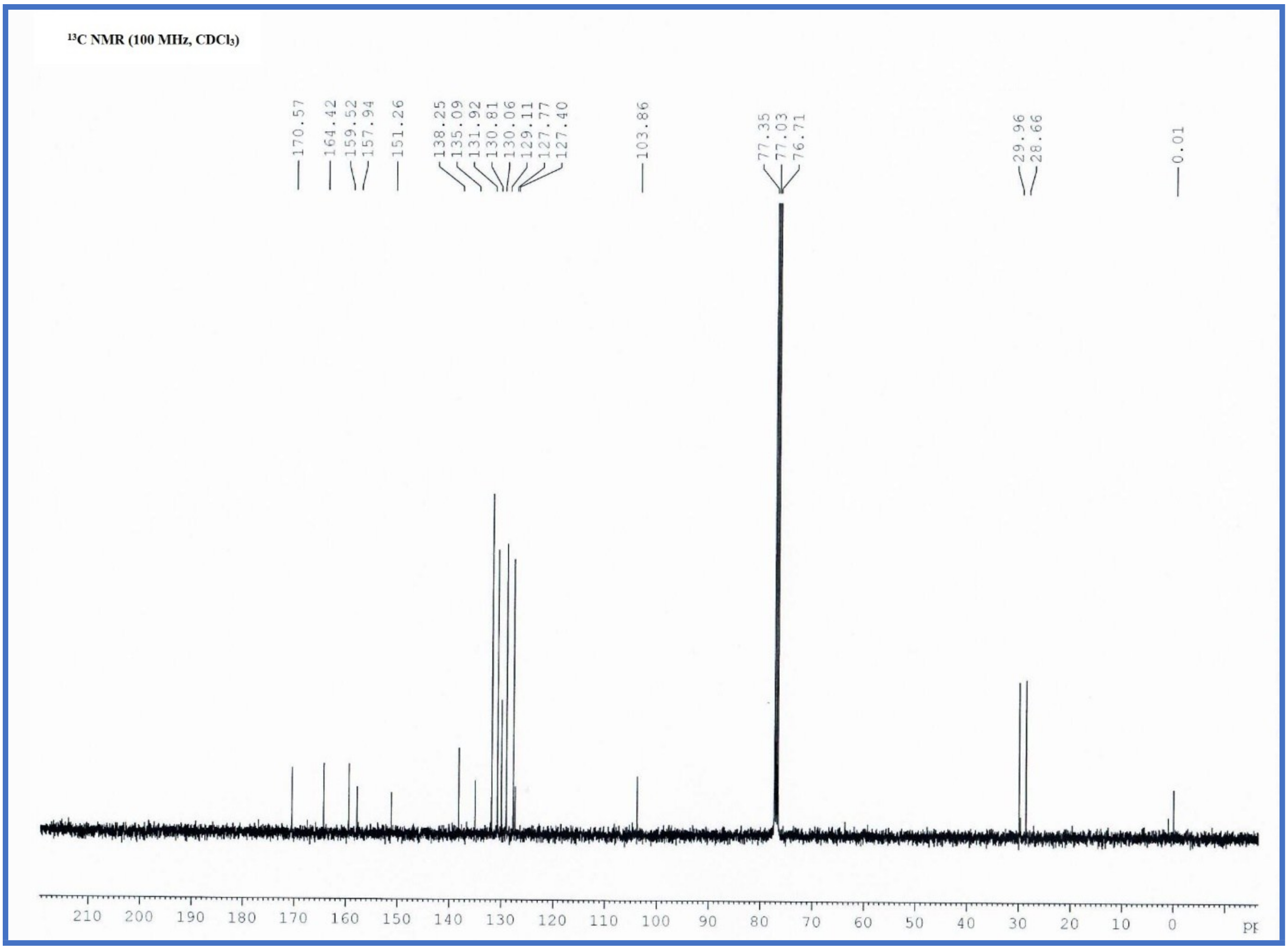

13C NMR (100 MHz, CDCl3)
170.57
164.42
159.52
157.94
151.26
138.25
135.09
131.92
130.81
130.06
129.11
127.77
127.40
103.86
77.35
77.03
76.71
29.96
28.66
0.01
210 200 190 180 170 160 150 140 130 120 110 100 90 80 70 60 50 40 30 20 10 0

1-Benzyl-3-methyl-5,7-diphenylpyrimido[4,5-*d*]pyrimidine-2,4(1*H*,3*H*)-dione (3t):

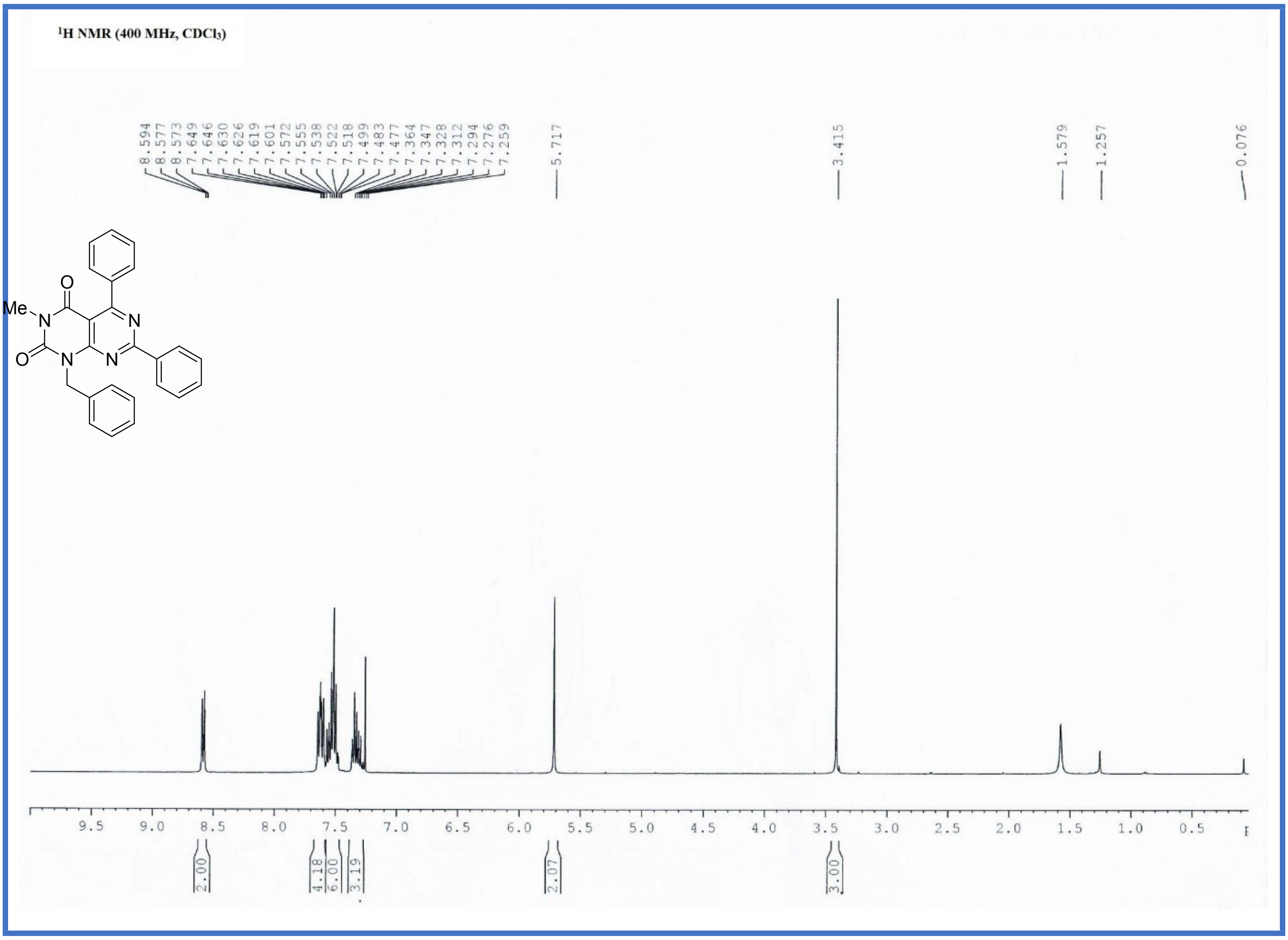

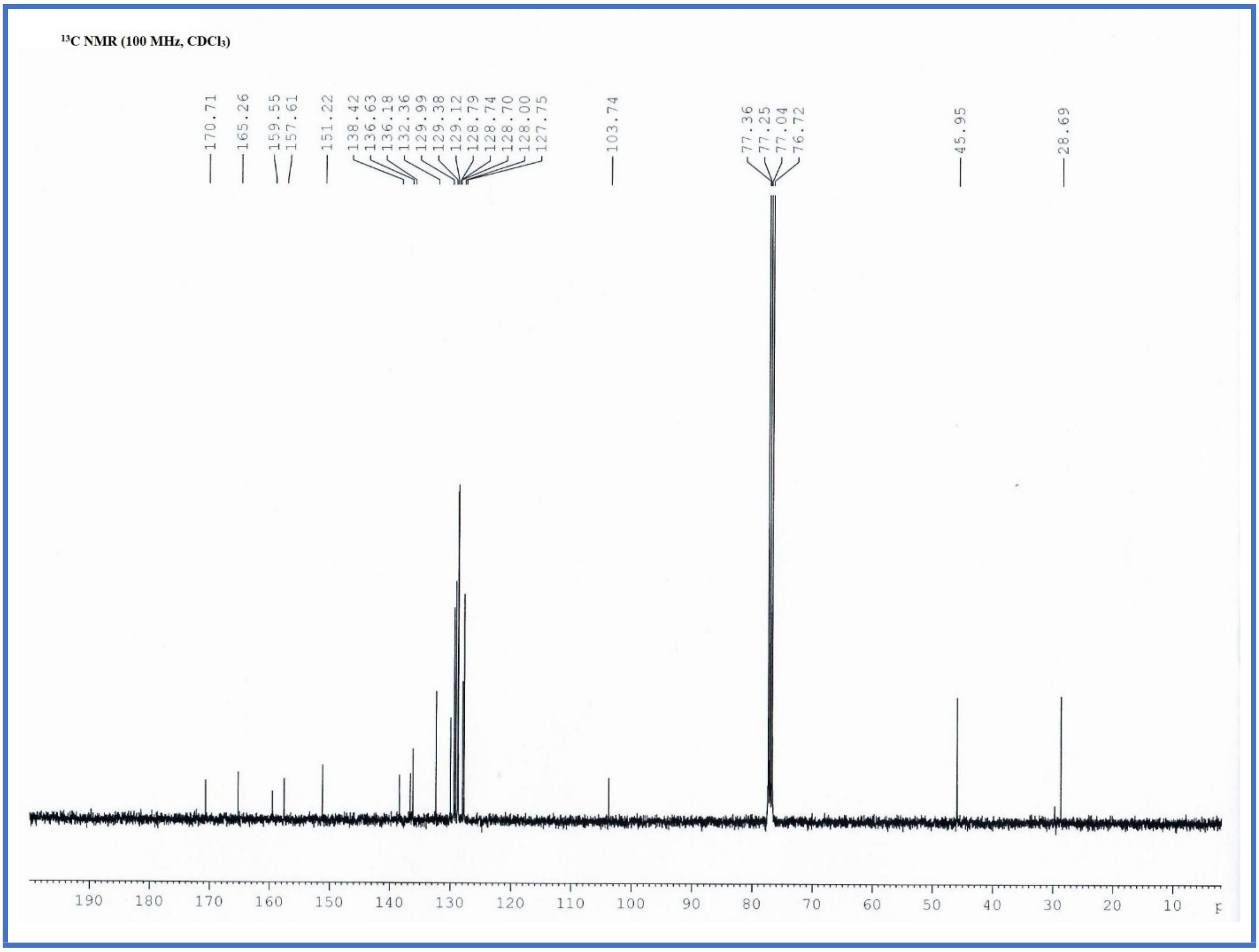
13C NMR (100 MHz, CDCl3)
170.71
165.26
159.55
157.61
151.22
138.42
136.63
136.18
132.36
129.99
129.38
129.12
128.79
128.74
128.70
128.00
127.75
103.74
77.36
77.25
77.04
76.72
45.95
28.69
190 180 170 160 150 140 130 120 110 100 90 80 70 60 50 40 30 20 10

1,3-Dibenzyl-5,7-diphenylpyrimido[4,5-*d*]pyrimidine-2,4(1*H*,3*H*)-dione (3u):

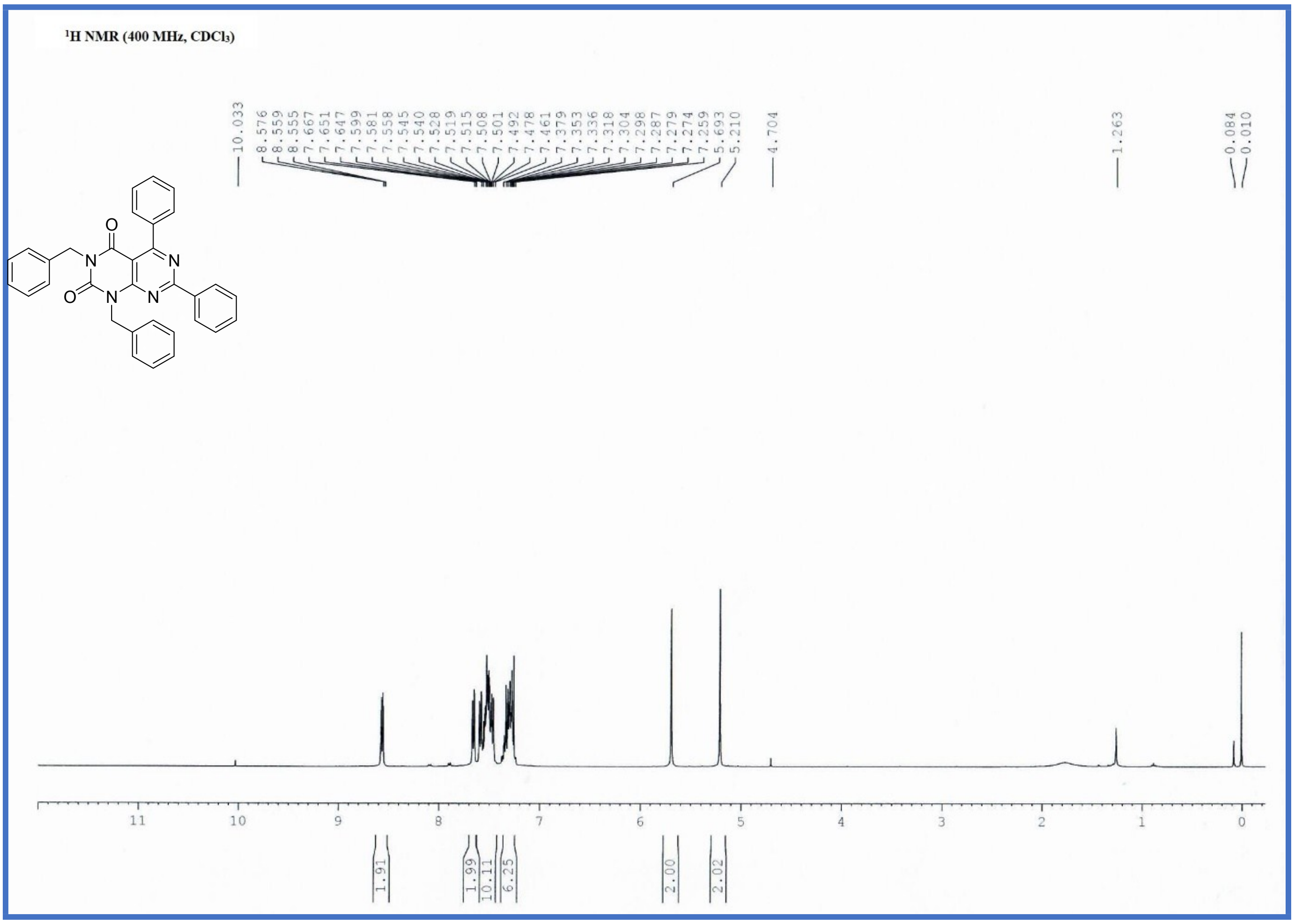

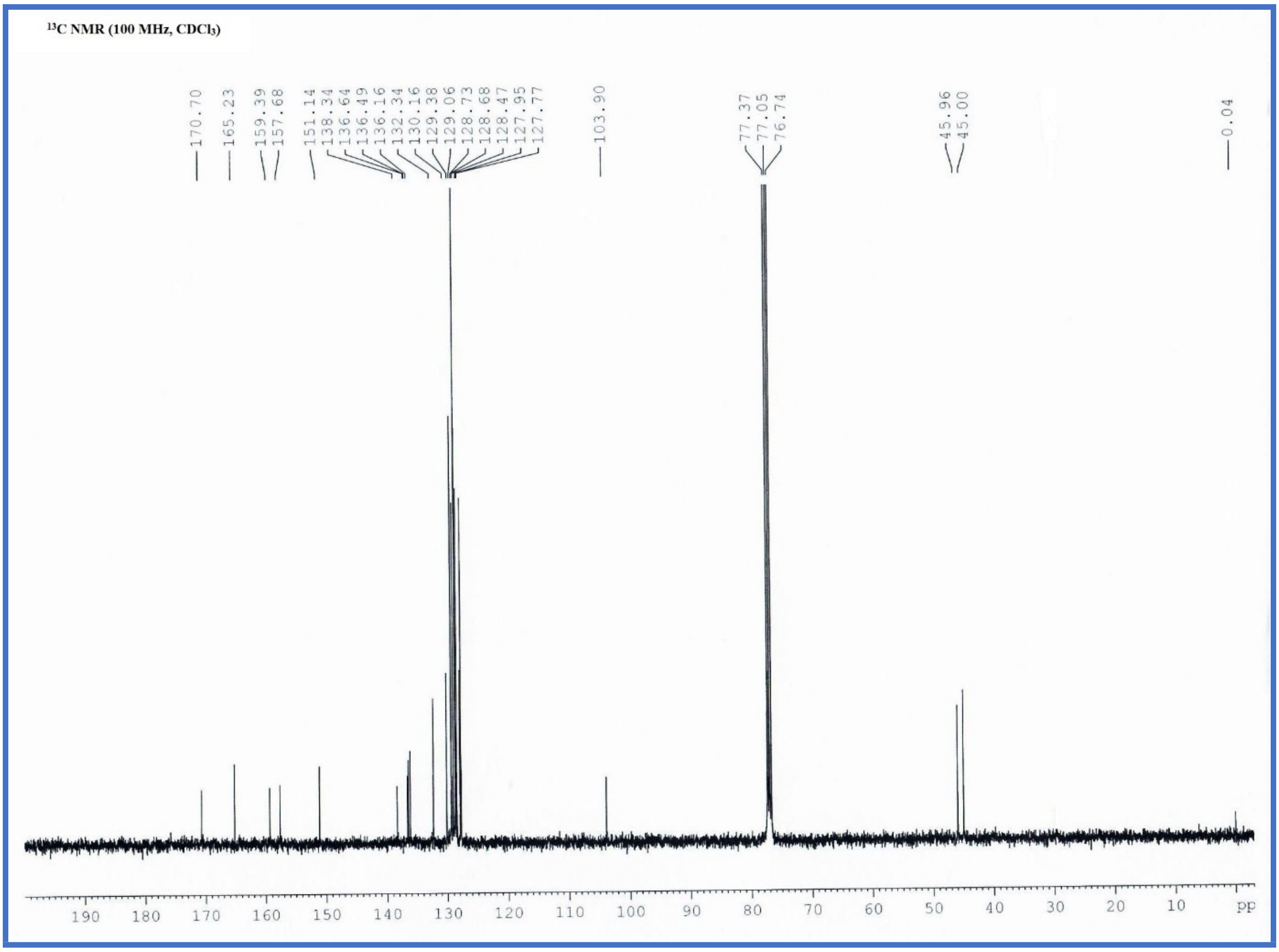
13C NMR (100 MHz, CDCl3)
170.70
165.23
159.39
157.68
151.14
138.34
136.64
136.49
136.16
132.34
130.16
129.38
129.06
128.73
128.68
128.47
127.95
127.77
103.90
77.37
77.05
76.74
45.96
45.00
0.04
190 180 170 160 150 140 130 120 110 100 90 80 70 60 50 40 30 20 10 pp

1,3-Dimethyl-5,7-diphenyl-5,6-dihydropyrimido[4,5-*d*]pyrimidine-2,4(1*H*,3*H*)-dione (3a/):

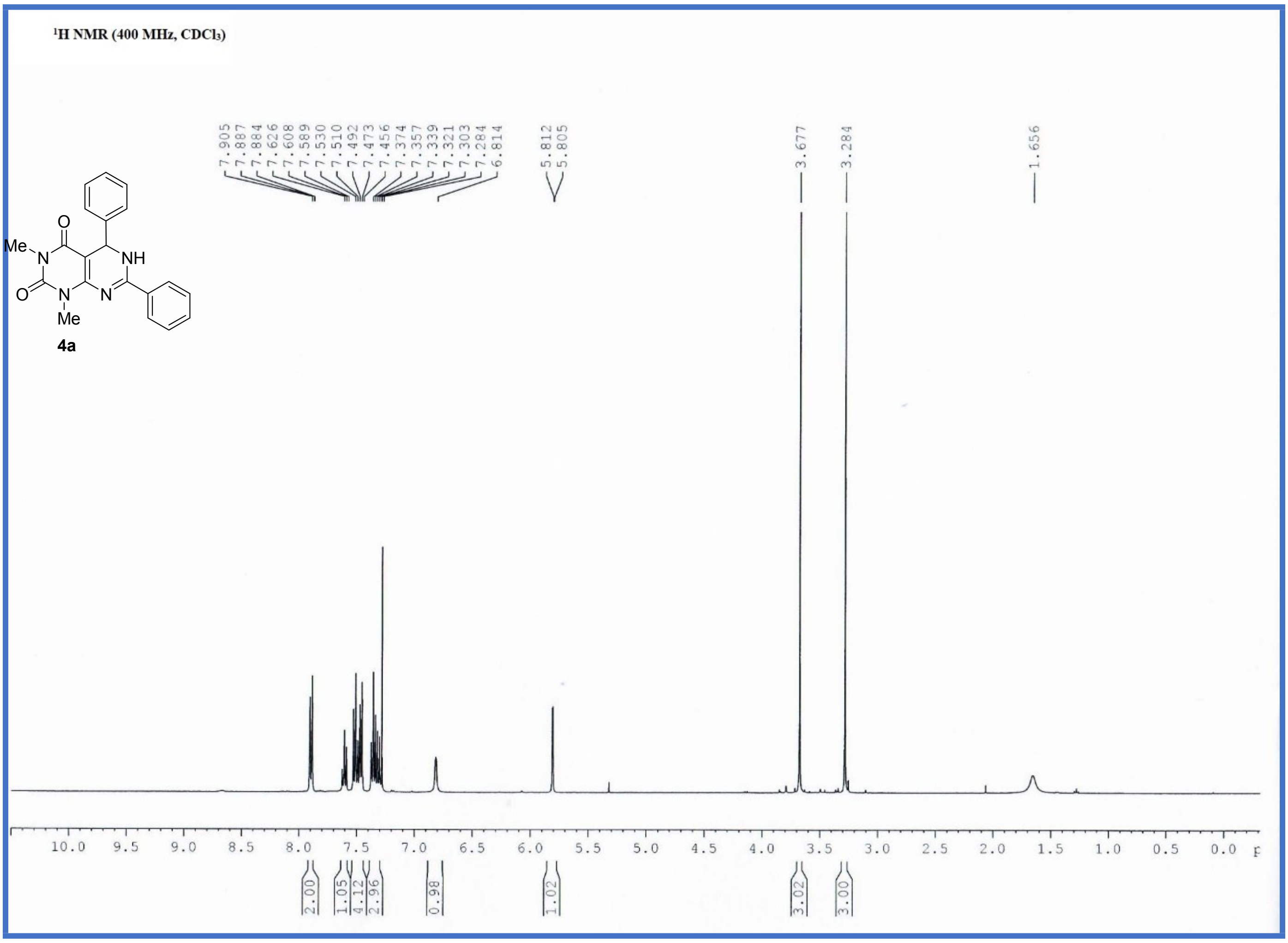

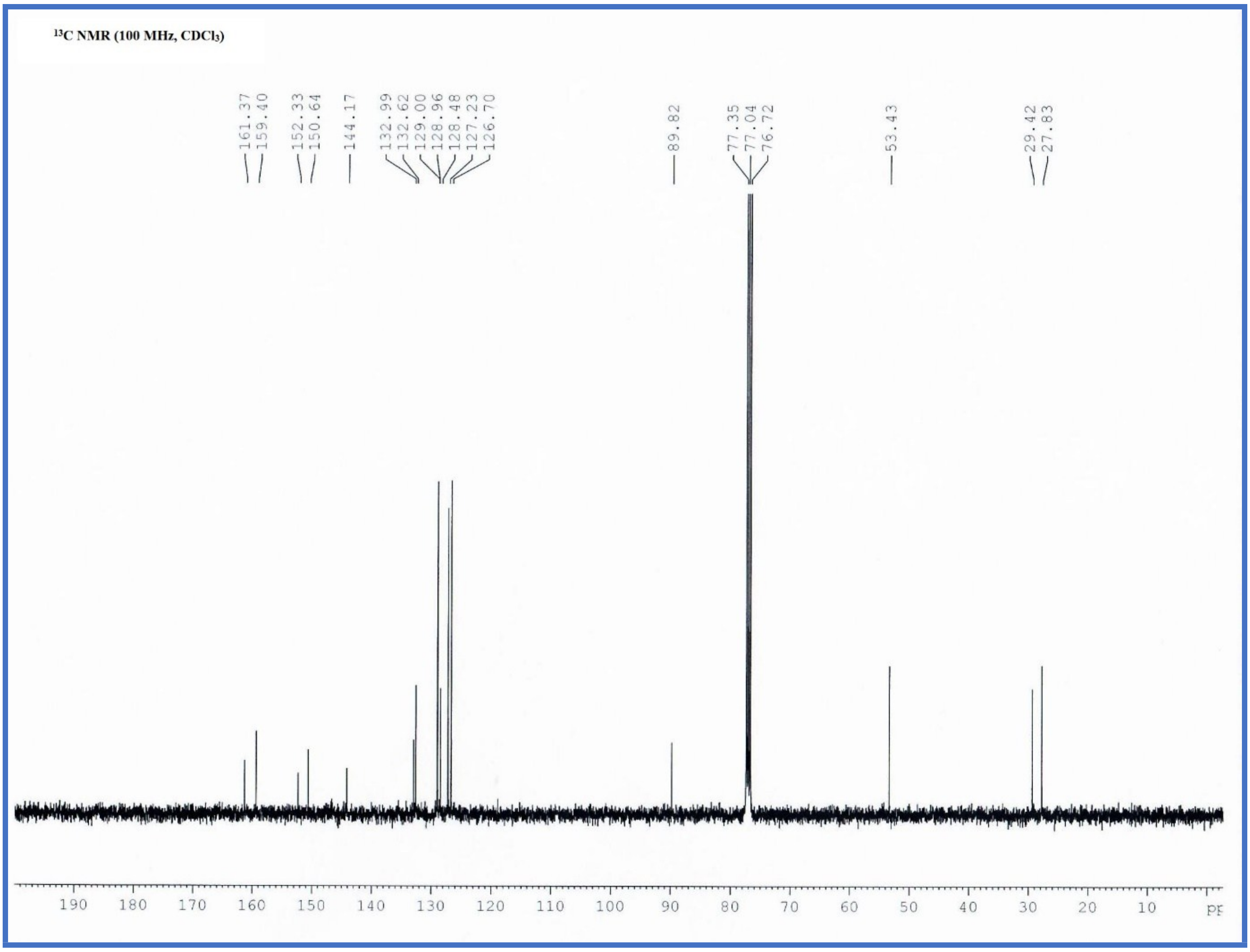
13C NMR (100 MHz, CDCl3)
161.37
159.40
152.33
150.64
144.17
132.99
132.62
129.00
128.96
128.48
127.23
126.70
89.82
77.35
77.04
76.72
53.43
29.42
27.83
190 180 170 160 150 140 130 120 110 100 90 80 70 60 50 40 30 20 10
